# The emerging role of human transmembrane RGD-based counter-receptors of integrins in health and disease

**DOI:** 10.1186/s11658-025-00787-7

**Published:** 2025-10-02

**Authors:** Carlos Cabañas, Elisa Rossi, Ruben A. Bartolomé, Kai Doberstein, Peter Altevogt, J. Ignacio Casal, Carmelo Bernabeu

**Affiliations:** 1 Cell–Cell Communication and Inflammation Unit, Centro de Biología Molecular “Severo Ochoa” (CSIC-UAM), 28049 Madrid, Spain; 2 INSERM, Optimisation Thérapeutique en Neuropharmacologie OTEN U1144, Université Paris-Cité, 75006 Paris, France; 3 Department of Biomolecular Medicine, Centro de Investigaciones Biológicas “Margarita Salas”, Consejo Superior de Investigaciones Científicas (CSIC), 28040 Madrid, Spain; 4 Department of Obstetrics and Gynecology, Medical Faculty Mannheim of the Heidelberg University, 68167 Mannheim, Germany; 5 Mannheim Institute for Innate Immunoscience, Medical Faculty Mannheim of the Heidelberg University, 68167 Mannheim, Germany; 6 Skin Cancer Unit, German Cancer Research Center (DKFZ) and Department of Dermatology, Venereology and Allergology, University Medical Center Mannheim, Heidelberg, Germany

**Keywords:** Endoglin, L1CAM, ADAM15, Cadherin-17, Cadherin-5, Cadherin-6, Integrins, Cell–cell adhesion, Membrane counter-receptors, RGD motif

## Abstract

Most of the canonical Arg-Gly-Asp (RGD)-containing integrin ligands are extracellular matrix proteins, such as fibronectin, vitronectin and fibrinogen, which regulate cell-ECM adhesion processes. However, during the last years, several reports have demonstrated the existence of non-canonical RGD-containing integrin ligands that are cell surface transmembrane proteins. At variance with the canonical extracellular matrix integrin ligands, the RGD-containing cell surface integrin ligands are involved in cell–cell adhesion processes and function as “integrin counter-receptors”. We propose in this review grouping these transmembrane proteins, which include endoglin, cadherin-5, cadherin-6, cadherin-17, ADAM15, and L1CAM, under the newly coined acronym RGD-ICRs (RGD-containing Integrin Counter-Receptors). We present and discuss the structure of RGD-ICRs, their RGD-based interactions with integrins, the specific signaling pathways triggered in different cell types, as well as their pathophysiological involvement. It can be postulated that RGD-ICRs constitute an emerging group of non-canonical RGD-based integrin counter-receptors. In spite of being encoded by different and independent genes and involved in different pathophysiological processes, all of them appear to have undergone a strong evolutionary convergence in order to acquire the same functional capacity to bind integrins via the RGD motif. Importantly, these RGD-ICRs are also emerging as novel biomarkers and therapeutic targets, with promising clinical potential in a wide array of pathologies.

**Graphical Abstract:**

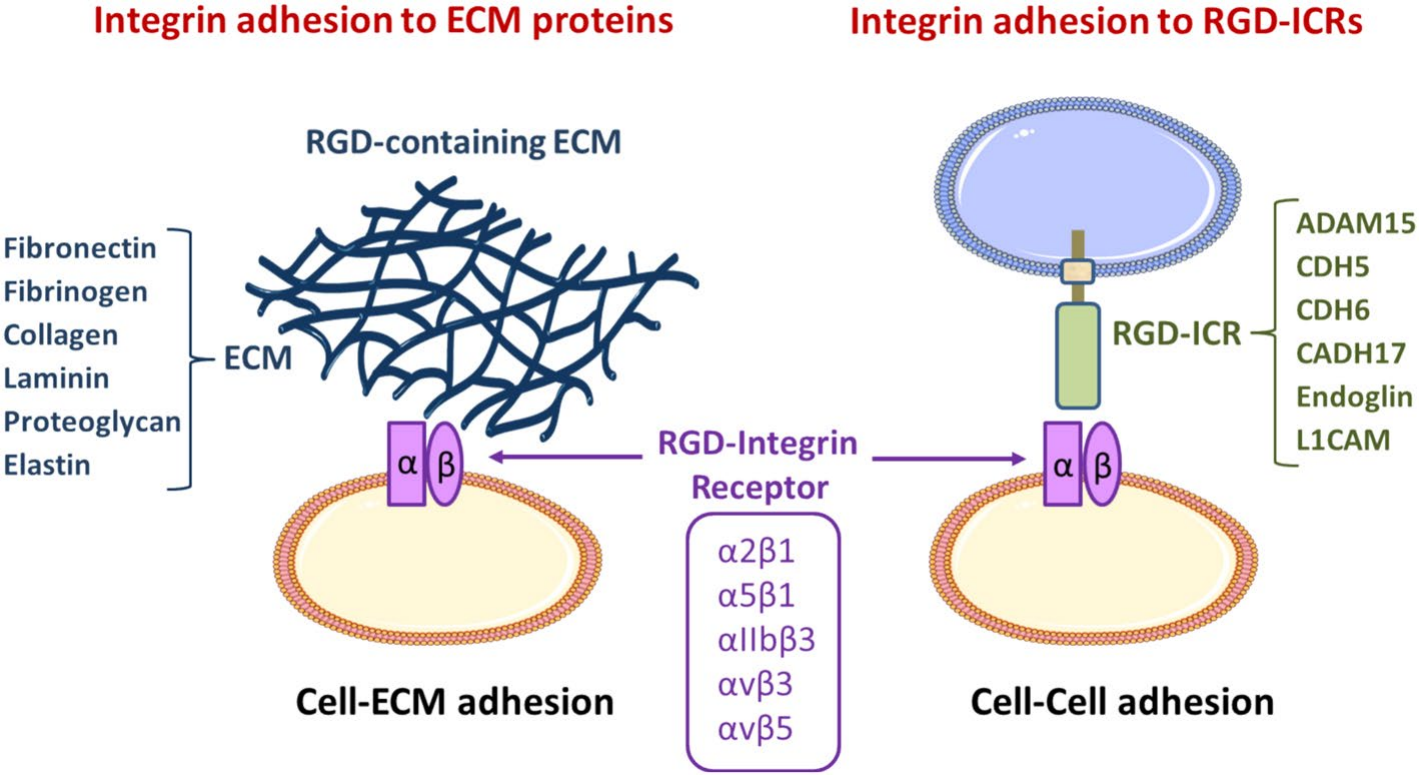

**Supplementary Information:**

The online version contains supplementary material available at 10.1186/s11658-025-00787-7.

## Introduction

Integrins are an evolutionary conserved family of surface receptors that mediate crucial intercellular (cell–cell), cell–extracellular matrix (ECM) and cell–pathogen adhesion phenomena, through interactions with their specific ligands. Despite their strong involvement in cell adhesion processes, integrins should not be regarded merely as adhesion receptors, because they also work as important cell signaling receptors. In this regard, integrins transmit signals in a bidirectional manner that involves inside-out as well as outside-in signal transduction mechanisms (reviewed in [1–5]). Considering this dual nature of integrins, as adhesion and signaling molecules, it is not surprising that these receptors are crucially involved in many physiological processes, including embryo development, acquisition and maintenance of tissues and organ architecture, blood clotting, wound healing, angiogenesis, cell migration, leukocyte recirculation, and immune responses. Furthermore, the deregulation of integrin expression and/or function have strong implications also in many pathological phenomena, including tumor cell invasion and metastasis, atherosclerosis and thrombosis, myocardial infarction, brain stroke, ischemia–reperfusion syndrome, immunodeficiencies (leukocyte adhesion deficiencies, LADs) and autoimmune inflammatory disorders (asthma, arthritis, inflammatory bowel disease [IBD], multiple sclerosis, psoriasis, and so on).

Structurally, integrins are heterodimeric transmembrane proteins formed by the noncovalent association of an α and a β polypeptide subunit. The pairing of 14 different α subunits with 8 different β subunits gives rise in vertebrates to 24 distinct integrins. In several cases, one particular α subunit is found only in one integrin heterodimer, as it is the case for αL, αM, αX and αD subunits, that can only associate with the β2 subunit to form integrins αLβ2, αMβ2, αXβ2 and αDβ2, respectively. In other cases, however, one α subunit can associate with different β subunits, as in the case of αV, which is found in integrins αVβ1, αVβ3, αVβ5, αVβ6 and αVβ8. Likewise, several β subunits can only associate with a particular α subunit (for instance, the subunit β4 is only found in integrin α6β4), while other β subunits can associate with up to 12 different α subunits, as in the case of the β1 subunit (which can be found in integrins α1β1, α2β1, α3β1, α4β1, α5β1, α6β1, α7β1, α8β1, α9β1, α10β1, α11β1, and αVβ1). Therefore, there exists a high degree of promiscuity in the associations between α and β integrin subunits.

**Fig. 1:**
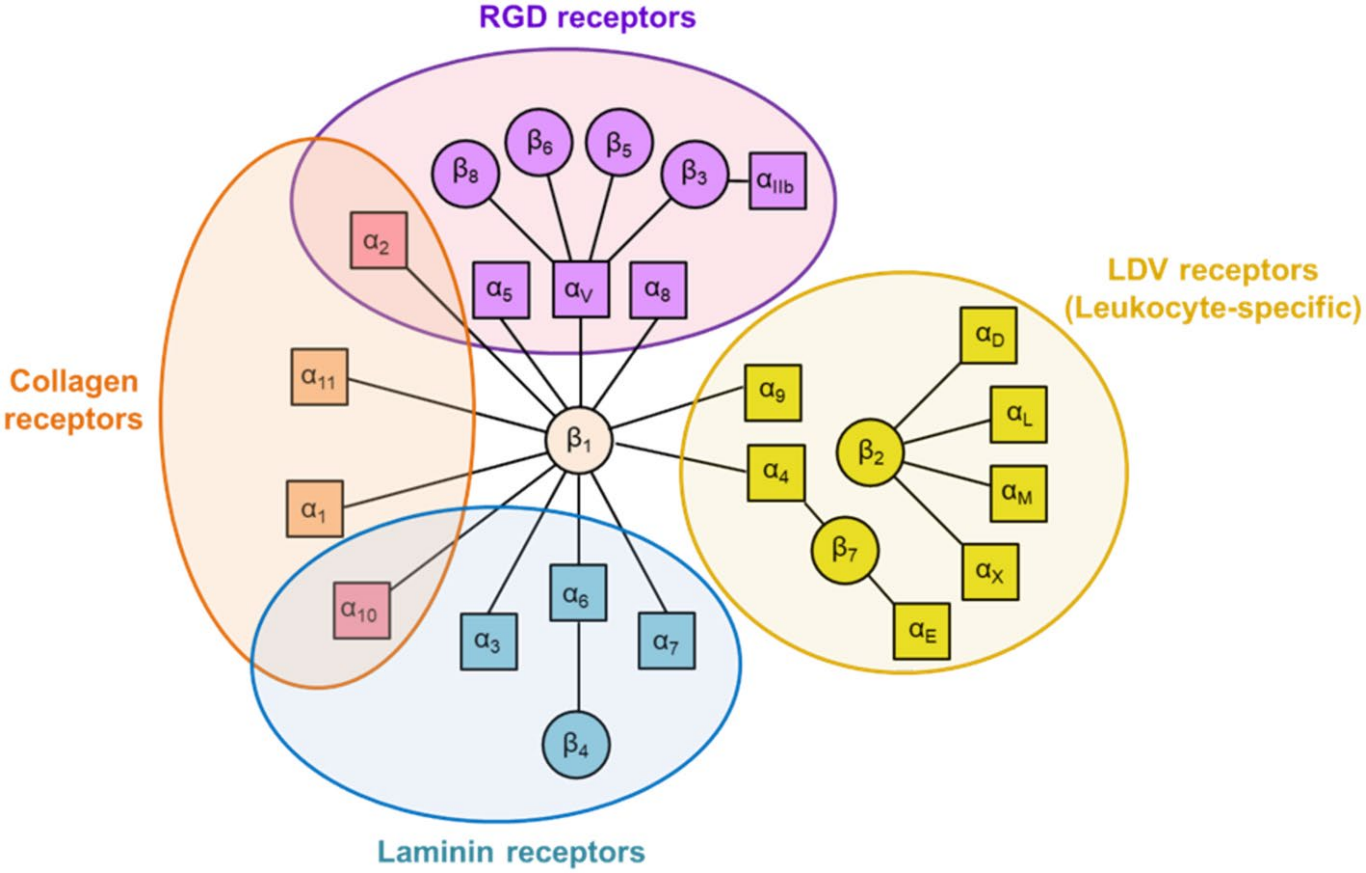
Classification of the integrin receptor family into four large groups according to their ligand specificity, recognition motifs, and restricted expression on leukocytes. All integrin heterodimers are formed by the noncovalent association of distinct α and β subunits. Historically, the integrins were initially grouped into different subfamilies according to their β-chains (β1-, β2-, β3-subfamilies), but now, these categorizations are less in use. RGD, Arg–Gly–Asp; LVD, Leu–Asp–Val

Integrins have been categorized into different subgroups on the basis of their subunit composition as well as on other criteria, such as their ligand-binding specificity or their restricted expression in selected cell types. When the first integrins were discovered in the 1980 s, they were initially classified into three subfamilies (termed β1-, β2-, and β3-integrins), according to the three β subunits identified at that time, which were shared by different α subunits [6]. Integrins within the β1-subfamily were also termed “very late activation” (VLA) antigens because the expression of the first identified members of this subfamily (i.e., α1β1 = VLA-1 and α2β1 = VLA-2) was found to be highly upregulated on T lymphocytes after a long period (several days to weeks) of in vitro activation with antigen or mitogens [6–8]. Likewise, other integrins were grouped into the β2-subfamily, which comprises the αLβ2 (leukocyte function-associated antigen-1, LFA-1), αMβ2 (macrophage antigen-1, Mac-1), αXβ2 (gp150/95 antigen), and αDβ2 members. Interestingly, the expression of the members of the β2-integrin subfamily is restricted to specific subtypes of leukocytes, as these molecules serve fundamental functions in the immune system [9–11]. However, as more α and β integrin subunits were subsequently identified, it became clear that this simple classification of integrins into β1-, β2-, and β3-subfamilies was no longer appropriate. Thus, integrins have been further categorized according to other criteria, such as: (i) the type of ligand proteins they recognize (e.g., collagen-receptor or laminin-receptor integrins); (ii) the specific sequences they recognize on their ligands (e.g., Arg–Gly–Asp [RGD]- or Leu–Asp–Val [LDV]-recognizing integrins); and (iii) their restricted cell expression patterns (e.g., leukocyte-specific or platelet-specific integrins) [12, 13] (Fig. 1).

Integrin binding typically occurs through the recognition of specific short peptide sequences (motifs) on their ligands. Interestingly, these integrin-recognized motifs within ligands almost invariably contain an oxygenated acidic residue (E or D) [14, 15]. Intensive research effort conducted over the last 40 years has clearly established that all integrins require the presence of divalent cations in the extracellular medium to bind their ligands [2, 14–18]. The molecular basis for this absolute requirement is that a divalent cation (Mg^2+^under physiological conditions) acts as a bridge between the integrin’s metal ion-dependent adhesion site (MIDAS) and an acidic residue (either E or D) present in specific sequences on the ligand molecules [2, 14, 15]. The MIDAS site is a discontinuous motif formed by the side chains of five oxygenated coordinating residues (DxSxS/T/D) contributed by three different polypeptidic loops of the integrin protein, which collectively, together with a sixth oxygenated residue (E or D) contributed by the ligand, bind the Mg^2+^ cation. One MIDAS site is always present in the I-domain of all integrin β subunits (termed the β-I domain) and another MIDAS site is also present in a subset of integrin α subunits that contain an additional inserted I-domain (termed the α-I domain). These MIDAS sites are a signature of all integrin I domains [2, 14, 15].

An in-depth coverage of the multiple ligands, expression and functional activation of integrins is out of the scope of this review, but readers are referred to excellent reviews covering these different aspects of integrin biology [2, 14, 15, 19–22].

The two best characterized specific sequences recognized by numerous integrins on their ligands are the motifs Arg–Gly–Asp (RGD) (recognized by integrins α2β1, α5β1, α8β1, αIIbβ3, αVβ3, αVβ5, αVβ6 and αVβ8) and Leu–Asp–Val (LDV) (recognized by integrins α4β1, α4β7, α9β1, αEβ7, αLβ2, αMβ2 and αXβ2) [13, 14, 19, 23–27].

Many canonical RGD-containing integrin ligands are extracellular proteins, such as fibronectin, vitronectin, and fibrinogen, which typically mediate crucial cell–ECM adhesion phenomena. However, several noncanonical RGD-containing integrin ligands are cell surface transmembrane proteins (also termed “integrin counter-receptors”) that are rather involved in cell–cell adhesion processes. Therefore, upon integrin recognition and binding of these RGD-containing counter-receptors, specific signaling pathways can be triggered in cells from both integrin and ligand counterparts, leading to important phenotypic and functional changes in the interacting cells. In this review, we will present and discuss the RGD-based interactions of integrins with a selection of transmembrane counter-receptors, including endoglin, cadherin-5, −6, and −17, ADAM15, and L1CAM, which are of utmost relevance in different physiological and pathological settings. We further propose grouping together these transmembrane proteins under the newly coined acronym RGD-containing integrin counter-receptors (RGD-ICRs).

## Endoglin (CD105)

### Protein structure

**Fig. 2:**
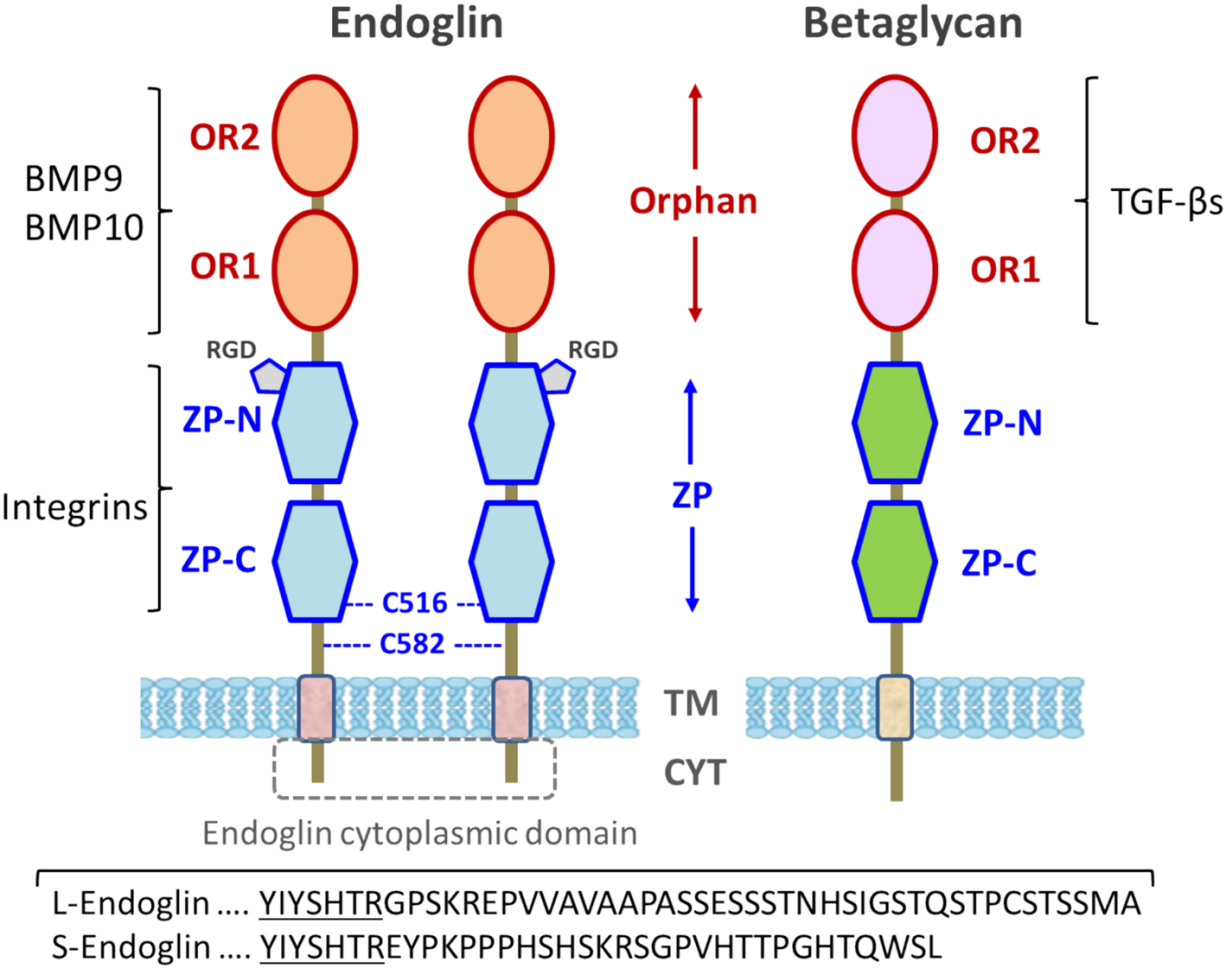
Structural representation of endoglin and betaglycan domains. Endoglin and betaglycan are type I membrane proteins characterized by a large extracellular domain, which includes a juxtamembrane zona pellucida (ZP) domain and an N-terminal orphan region (OR). Endoglin is a disulfide-linked dimer, while betaglycan is a monomer. Representative ligands (TGF-βs, BMPs and integrins) that bind to specific domains, the cytoplasmic (CYT) and transmembrane (TM) regions, and cysteines involved in endoglin disulfide bonds (C516 and C582) are indicated. Bottom: the cytoplasmic amino acid sequences shared by L-endoglin and S-endoglin isoforms are underlined. The scheme is not to scale. Part of this figure was created with BioRender.com

**Fig. 3:**
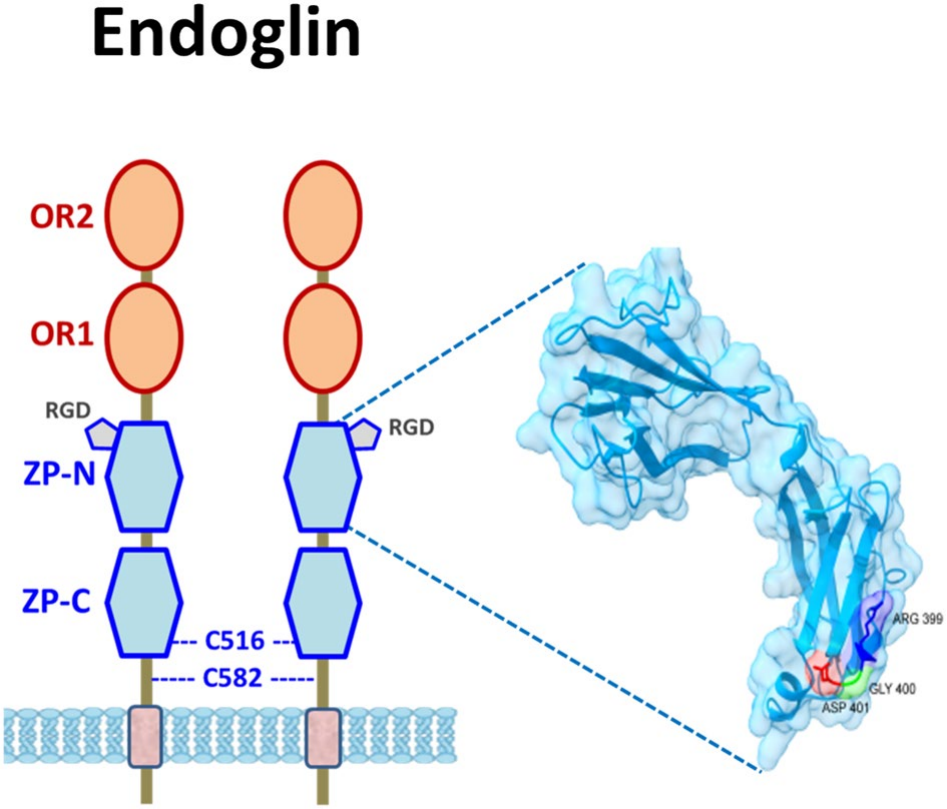
Schematic representation of endoglin structure. The orphan regions (OR1 and OR2), the zona pellucida domains (ZP-N and ZP-C), and the cysteines involved in disulfide bonds (C516 and C582), are indicated. The three-dimensional (3D) structure model on the right represents a fragment of the ZP-N subdomain encompassing the RGD motif. Part of this figure was created with BioRender.com

Human endoglin is a type I integral membrane protein characterized by a large extracellular domain comprising 561 amino acids, a single hydrophobic transmembrane segment, and a short cytoplasmic tail [28]**.** Two different alternatively spliced isoforms have been described as long (L) and short (S) endoglin [28–31] (Fig. 2). Human S-endoglin and L-endoglin proteins differ in the length and composition of their cytoplasmic tails, which contain 14 and 47 amino acids, respectively; notably, only seven amino acid residues are unique to S-endoglin. However, L-endoglin is the most abundantly expressed isoform in all tissues, and the functional studies discussed in this review will pertain to this isoform, named just as endoglin from now on. Endoglin is produced as a glycosylated homodimer, linked by disulfide bonds, with a molecular weight of 180-kDa. Based on its primary structure, endoglin possesses five potential *N*-linked glycosylation sites within its NH_2_-terminal domain, along with an *O*-linked glycan region enriched in serine and threonine residues [28]. Since the extracellular region of endoglin comprises the major portion (almost 90%) of the protein, it has been targeted for many structural and functional studies. This region includes two distinct domains: (i) a juxtamembrane zona pellucida (ZP) domain spanning approximately 260 amino acids, containing eight conserved cysteine residues, and divided into two subdomains, ZP-C at the C-terminus and ZP-N at the N-terminus, and (ii) an orphan region (OR) at the N-terminus, which does not share significant similarity with other known protein families and is subdivided in two orphan regions (OR1 and OR2) [32, 33]. These two domains show different functional activities from each other. The orphan regions OR1 and OR2 play a role in binding and signaling pathways of transforming growth factor β (TGF-β)/bone morphogenetic protein (BMP) family members such as BMP-9 and BMP-10 [33–35]. Conversely, the ZP domain contributes to cell adhesion by interacting with integrins of the RGD subfamily, such as α5β1 and αIIbβ3, which recognize the RGD motif situated within the ZP-N subdomain [36, 37]. Endoglin shares the highest homology with the proteoglycan betaglycan, including the same overall structure encompassing OR and ZP domains and the greatest similarity in their transmembrane (73%) and cytoplasmic (61%) regions (Fig. 2) [38, 39]. Endoglin and betaglycan also share other characteristics. Thus, membrane-bound endoglin and betaglycan can undergo proteolytic shedding, resulting in circulating forms. In addition, endoglin and betaglycan are auxiliary receptors for TGF-β/BMP familiy members, and, accordingly, both proteins have been clustered as TGF-β type III receptors. However, endoglin and betaglycan differ in: (i) their cellular distribution, endoglin being predominantly expressed in endothelial cells versus the ubiquitous presence of betagalycan; (ii) their ligand specificity, with endoglin and betaglycan preferentially mediating BMP or TGF-β signaling, respectively; (iii) their oligomeric nature, endoglin being a dimer, while betaglycan is a monomer; and (iv) the presence in endoglin of an RGD integrin recognition motif, which is absent in betaglycan (Fig. 3) [37–39]. In addition to humans (*Homo sapiens*), the RGD motif of endoglin is conserved among other primates, including orangutan (*Pongo abelii*), rhesus macaque (*Macaca mulatta*), and gorilla (*Gorilla gorilla*), whereas the RGD-related sequence Thr–Asp–Asp (TDD) is present in mouse (*Mus musculus*) and pig (*Sus scrofa*) endoglin instead [32, 40].

The transmembrane domain of endoglin contains a characteristic stretch of ~25 uncharged, mainly hydrophobic amino acids connecting the large extracellular region with the short cytoplasmic domain. Moreover, endoglin’s cytoplasmic domain undergoes constitutive phosphorylation at serines and threonines [41] and participates in organizing the actin cytoskeleton [42, 43] and TGF-β/BMP signaling [30, 44]. Interestingly, most of the cell surface-exposed region of endoglin may be shed as a circulating form, named soluble endoglin (sEng), following proteolytic cleavage by the metalloproteinases (MMP) MMP-14 and MMP-12 or thrombin [45–47]**.**

### Tissue distribution and functional activities

Endoglin is well conserved among vertebrates and has been described in human, mouse, rat, chicken, and zebrafish species, among others [32, 48, 49]**.** In humans, endoglin is predominantly expressed on proliferating endothelial cells and syncytiotrophoblasts and to a lesser extent in other cell types such as mesenchymal stem cells, macrophages, epithelial cells, and fibroblasts [37, 50]. Endoglin functions as a component within the TGF-β/BMP signaling cascade. As a coreceptor, endoglin associates with TGF-β type I and II receptors and the ligand to form a protein complex. Through its association with type I receptors ALK1 and ALK5, endoglin regulates ligand binding and signal transduction in endothelial cells [44, 51–53]. Several studies have demonstrated that endoglin and ALK1 cooperate in the same signaling route. Thus, members of the TGF-β superfamily, namely BMP-9 and BMP-10, exhibit strong binding affinity for endoglin, triggering the phosphorylation by ALK1 of Smad1/5/8. In turn, this phosphorylation mediates the increase of the transcriptional regulator inhibitor of DNA binding 1 (Id1). By contrast, endoglin counteracts ALK5-dependent TGF-β signaling and the subsequent phosphorylation of Smad3 leading to the upregulation of plasminogen activator inhibitor 1 (PAI-1). Overall, endoglin critically influences the shift between ALK1 and ALK5 signaling dynamics in endothelial cells. Maintaining this balance is critical during vascular remodeling, endothelial proliferation, vascular quiescence, vascular tone, and endothelial-driven angiogenesis [54, 55]. Endoglin plays a vital role in angiogenesis and regulates the vascular tone via upregulation of endothelial nitric oxide synthase (eNOS) and downregulation of cyclooxygenase-2 expression and activity [52, 56–61]. Of note, S-endoglin is upregulated during endothelial cell senescence and acts in opposition to L-endoglin. S-endoglin can associate with both type I receptors in endothelial cells but displays a significantly higher affinity for ALK5 than for ALK1. As a result, S-endoglin suppresses cell proliferation and enhances the expression of the ALK5-responsive gene PAI-1, while downregulating the ALK1 target gene ID1 [51, 53].

While most endoglin studies so far have focused on endothelial cells, there is an emerging interest for investigating the endoglin function in additional cell types such as keratinocytes, mesenchymal stem cells, fibroblasts, hepatic stellate cells, macrophages, and cells from the hair follicle [50, 62–66].

### Role in RGD-dependent integrin-mediated cell adhesion

Multiple experimental findings indicate that endoglin plays a role in integrin function. Thus, the capacity of endothelial endoglin to act as a counter-receptor for integrins present in circulatory cells has been demonstrated during the last decade. The upregulated endoglin expression at inflammatory sites and areas of leukocyte infiltration has prompted studies exploring endoglin’s involvement in leukocyte trafficking. Following in vivo treatment with the inflammatory agents lipopolysaccharide (LPS) or carrageenan, leukocyte transendothelial migration to the peritoneum or lungs was significantly reduced in endoglin heterozygous (*Eng*^+*/–*^*)* mice compared with wild-type (*Eng*^+*/*+^) animals [40]. In addition, the transmigration of human leukocytes through monolayers of endoglin- and mock-transfected cells was markedly increased by endoglin. Interestingly, leukocyte transmigration was also enhanced across transwells coated with the RGD-containing ectodomain of endoglin, while this increased motility was reduced by soluble endoglin. Upon stimulation with the inflammatory chemokine CXCL12, an activator of integrins, leukocytes exhibited strong adhesion to plates coated with endoglin and to endothelial cells expressing endoglin. These endoglin-dependent adhesions were suppressed by inhibitory antibodies against integrin α5β1, RGD peptides, or AMD3100, an inhibitor of chemokine receptor signaling [40]. These findings show that endothelial endoglin engages with leukocyte integrin α5β1 through its RGD motif and that this adhesion is promoted by the inflammatory chemokine CXCL12, highlighting a regulatory function of endoglin in transendothelial leukocyte migration.

Interactions between human endothelial endoglin and platelet integrins have also been reported [67]. A fine and complex interplay between activated endothelium and platelets takes place in the course of thromboinflammatory responses, at vascular injury sites, and in the regulation of vascular hemostasis. Because endoglin participates in both inflammatory processes and integrin-mediated cell adhesion and migration through its RGD motif, the involvement of endoglin in mediating interactions between endothelial cells and platelets via integrin recognition was investigated. The ectodomain of endoglin was found to promote selective platelet adhesion in static environments and strengthen the retention of adherent platelets under flow conditions. Additionally, platelets adhered to confluent endothelial cells through a process mediated by endoglin. The above results prompted additional investigation on αIIbβ3 integrin, the primary RGD-dependent adhesion receptor on platelets. Of note, Chinese hamster ovary (CHO) cells, engineered to express human αIIbβ3 integrin, were able to specifically bind to endoglin-expressing adherent cells. However, platelets from patients with Glanzmann’s thrombasthenia, which lack the αIIbβ3 integrin [68], exhibited impaired endoglin-mediated adhesion to endothelial cells. Since platelets play a central role in hemostasis, the potential involvement of endoglin in this biological process was investigated. Indeed, bleeding time was significantly extended and prothrombin time remained unchanged in *Eng*^+/−^ mice compared with wild-type controls. Together, these findings indicate that endothelial endoglin contributes to αIIbβ3 integrin-mediated platelet adhesion to the endothelium, shedding light on key cellular mechanisms involved in hemostasis and thromboinflammation. Furthermore, the circulating form of human endoglin (sEng), encompassing an RGD motif, has also been shown to bind integrin αIIbβ3 [37]. Thus, supplementing human whole blood with sEng under flow conditions led to the formation of smaller thrombi. sEng also impaired platelet aggregation and thrombus retraction by disrupting fibrinogen binding, while having no effect on platelet activation. Surface plasmon resonance (SPR) binding assays confirmed a specific interaction between αIIbβ3 and sEng, while molecular modeling highlighted a favorable alignment of their structures, particularly through the endoglin RGD motif, suggesting a robust αIIbβ3/sEng complex. Interestingly, sEng-overexpressing mice (*hsEng⁺*) exhibited prolonged bleeding time and a higher frequency of rebleeding events compared with wild-type controls. By contrast, prothrombin time (PT) values did not differ between the two genotypes. In addition, after FeCl_3_-induced injury of the carotid artery, *hsEng⁺* mice exhibited a higher number of released emboli and a delayed occlusion compared with control animals. These data indicate that sEng disrupts thrombus formation and stability, likely compromising the binding of platelet αIIbβ3 to fibrinogen, and implying a regulatory role in primary hemostasis. Additional experimental evidences suggest a role for sEng in integrin-mediated processes: (i) the activity of membrane-bound endoglin by competing for its interaction with integrins like α5β1 and αIIbβ3 [36, 40, 67]; (ii) vessel stability by disrupting integrin-dependent interactions between endothelial and perivascular cells [36]; and (iii) interactions mediated by endoglin between platelets and endothelium [67].

The active role of endoglin regulating mural cell adhesion in the circulatory system has also been analyzed [36]. Blood vessels are surrounded and supported by vascular mural cells, including vascular smooth muscle cells or pericytes, whose dynamic relationship with endothelial cells play a pivotal role in vascular biology. The adhesion between vascular endothelial cells and mural cells was shown to be stimulated by integrin activators and suppressed by (i) silencing of β1-integrin or endoglin or (ii) treatment with sEng, anti-integrin α5β1 antibodies, or RGD peptides. Furthermore, by analyzing different endoglin mutants, the RGD motif was mapped as a key element involved in this cellular adhesion process, whereas *Eng*^+/−^ mice revealed a pericyte-dependent increase in vascular permeability compared with control animals. The adhesive capacity of endoglin was also investigated in podocytes, which are kidney glomerular cells that encase capillaries and contribute to an effective filtration barrier. Podocytes bind to the glomerular basement membrane, namely via α3β1 integrin, a receptor for laminin, along with the collagen receptor α2β1 integrin and the vitronectin receptor αvβ3, members of the RGD subfamily of integrins. Of note, overexpression of sEng in a transgenic mouse model for preeclampsia caused increased podocyturia, a parameter of renal disease progression. This observation suggests that by interacting with integrins, sEng contributes to the podocyte detachment from glomerular capillaries in this animal model. Overall, the above data point out to an essential function of endoglin in integrin-dependent mural cell adhesion [36].

Aside from the contribution of endoglin to integrin-mediated cell adhesion, described above, endoglin has also been reported to participate in an integrin-mediated crosstalk with the TGF-β/BMP signaling cascade [69]. Thus, α5β1 integrin and its ligand fibronectin promote the formation of an endoglin/ALK1 endothelial cell surface complex and the subsequent increase of Smad1/5/8 signaling. Conversely, TGF-β1 activates α5β1 integrin and triggers the downstream signaling to focal adhesion kinase (FAK) through an endoglin-dependent mechanism. Moreover, α5β1 integrin associates with endoglin at the cell surface, and the internalization of the resulting complex regulates α5β1 integrin activation and signaling [69]. This conclusion is in agreement with data obtained in the myeloid context, where endoglin is upregulated during the differentiation from nonadherent monocytes to adherent macrophages [70]. Differential gene expression analyses of human monocytic cells expressing endoglin revealed a marked alteration in biological processes associated with “cellular movement,” such as cell adhesion and transmigration, processes primarily governed by adhesion molecules such as α1, αL, αM and β2 integrins, which were also significantly regulated in the endoglin-expressing cells [71].

The interplay between endoglin and integrins has also been postulated to be involved in bacterial infections, more specifically in the shedding of infected epithelial cells, also called exfoliation, which acts as a natural defense to inhibit bacterial colonization [72, 73]. Thus, the engagement of carcinoembryonic antigen-related cell adhesion molecules (CEA-CAMs) by various human pathogens promotes endoglin expression. Notably, endoglin silencing suppressed cell adhesion induced by infection, whereas ectopic expression of endoglin triggered cell adhesion to ECM and prevented infection-induced cell detachment. While the endoglin-driven increase in cell adhesion required integrin β1, endoglin expression did not increase cellular integrin levels but markedly enhanced the capacity of the cells to bind ECM, indicating a role for endoglin in regulating integrin activity [73]. The same authors also reported that in carcinoembryonic antigen (CEA) transgenic mice, but not in wild-type controls, CEA-binding bacteria established colonization in the urogenital tract by preventing the exfoliation of mucosal cells. Interestingly, binding of bacteria to CEA stimulated the expression of endoglin, which altered the focal adhesion composition and activated β1 integrins. These findings suggest that endoglin may serve as a promising therapeutic target in the treatment of bacterial infections [72].

### Pathophysiological involvement and implications

Since the early 1990 s, accumulated information supports the involvement of both membrane-bound endoglin and soluble endoglin in a wide spectrum of pathophysiological processes, including angiogenesis, endothelial dysfunction, fibrosis, inflammation, vascular pathology, hemostasis, hypertension, and cancer. In addition, the impact of endoglin on the diagnostics, etiology, or prognosis in various diseases such as hereditary hemorrhagic telangiectasia, preeclampsia, cancer, hyperglycemia, atherosclerosis, hypercholesterolemia, kidney and liver fibrosis, septic syndrome, nonalcoholic steatohepatitis (NASH), and systemic sclerosis has been reported [37, 38, 62, 74–78]. Several relevant examples are described in more detail below.

### Hereditary hemorrhagic telangiectasia

Endoglin is implicated in several angiogenesis-related conditions, including the rare vascular disease hereditary hemorrhagic telangiectasia (HHT), a heterogeneous autosomal dominant disorder with prevalence estimated at 1 in 5000. Loss-of-function mutations in the endoglin gene (*ENG*) lead to HHT type 1, which, together with the HHT type 2 (resulting from mutations in *ACVRL1/ALK1*), account for more than 90% of all patients with HHT diagnosed using the well-established clinical criteria of Curaçao [79–81]. Additional pathogenic variants in *SMAD4* and *GDF2* explain a reduced number (~2%) of HHT cases. *ENG*, *ACVRL1*, *SMAD4*, and *GDF2* encode components of the BMP/TGF-β signal transduction pathway in endothelial cells. Of note, endothelial cells are the primary cellular targets in HHT, where endoglin is abundantly present. HHT is hallmarked by arteriovenous malformations (AVMs) in the lung, liver, and brain, along with mucocutaneous telangiectases resulting in epistaxis and gastrointestinal (GI) hemorrhage. Recurrent epistaxis and/or GI bleeding are characteristic of HHT, leading to anemia secondary to iron deficiency. Affected patients may need long-term iron therapy via oral or intravenous routes, and blood transfusions in more critical cases. So far, no drug has received the Food and Drug Administration (FDA) or European Medicines Agency (EMA) authorization for treating HHT associated manifestations, but ongoing investigations are actively engaged to find novel therapeutic targets within signaling pathways where endoglin is involved [37, 80, 82]. In this context, several preclinical in vitro and in vivo disease models support the role of integrin-dependent cell adhesion of endoglin in HHT pathophysiology [36, 37, 40, 67]. Thus, endothelial endoglin binds to integrins present in vascular mural cells, contributing to vascular development, stabilization, and permeability. Of note, these biological processes are impaired in HHT vascular lesions, namely in mucocutaneous telangiectasias, which show vessel fragility, leading to hemorrhages [36]. Upon inflammation, the expression of endothelial endoglin is markedly upregulated and is linked to the infiltration of inflammatory leukocytes. Alongside inflammation, different cytokines are released, including the chemokine CXCL12, leading to the activation of leukocyte α5β1 integrin, which then binds to endothelial endoglin. This engagement allows the extravasation and migration of leukocytes to the inflammation-affected area, contributing to vascular remodeling, a process that is impaired in HHT [36, 40]. Endothelial endoglin can also bind to αIIbβ3 integrin in platelets from healthy subjects, but this binding is markedly reduced when using αIIbβ3-deficient platelets from patients with Glanzmann’s thrombasthenia [67]. Furthermore, the role of endoglin in platelet-dependent hemostasis parameters were analyzed. Thus, the bleeding time was significantly prolonged whereas prothrombin time remained unchanged in endoglin-haplodeficient (*Eng*^+/-^) mice (HHT1 model) compared with control wild type animals. Because hemostasis is critical during the nose and GI bleeding of patients with HHT, these results support the implication of αIIbβ3 integrin-mediated adhesion of endoglin to platelets in endothelial-dependent hemostasis [67, 83]. Interestingly, sEng, which contains the integrin-interacting region, has also demonstrated a role in the binding to platelet integrins. In fact, sEng disrupts thrombus formation and stabilization through its interaction with platelet αIIbβ3, indicating a role in the regulation of primary hemostasis and in thromboinflammatory events [37]. Due to the high frequency of infections seen in HHT [84], the role of endoglin in macrophages has been analyzed. The absence of endoglin in macrophages leads to a compromised immune response in mice, which may underlie a deficient innate immunity in HHT [64]. In fact, antibiotic prophylaxis is strongly advised prior to dental or invasive procedures in patients with HHT, particularly when pulmonary AVMs are confirmed or suspected [85].

### Preeclampsia

Soluble endoglin (sEng) can be shed from the cell surface transmembrane endoglin form upon the proteolytic activity of thrombin, MMP14 or MMP12 [45–47]. Numerous studies have shown that sEng is involved in preeclampsia, a multisystemic disorder of women characterized by the onset of hypertension or proteinuria developing beyond the 20th week of gestation in previously normotensive individuals. Globally, preeclampsia occurs in 2% to 10% of all pregnancies. Without proper treatment, preeclampsia may result in severe or even life-threatening complications for both the mother and the baby, such as seizures (eclampsia), liver dysfunction, or pulmonary edema [86, 87]. Increased levels of sEng in plasma is considered as a potential early biomarker for identifying and predicting the course of this disease. Indeed, concentration of sEng is increased in the serum of women with preeclampsia, correlates with the severity of the condition, and drops postpartum [88]. Of note, results with several animal models of preeclampsia support a pathogenic role of sEng. Thus, the administration of recombinant adenoviral human sEng to pregnant rats can lead to severe preeclampsia, encompassing hemolysis, elevated liver enzymes, and low platelet count (HELLP) syndrome and fetal growth restriction [88]. Furthermore, sEng-expressing transgenic mice exhibited elevated blood pressure, reduced pup size, proteinuria, podocyturia, and kidney damage, a phenotype that resembles preeclampsia [36, 89]. Additionally, wild-type pregnant mice carrying transgenic fetuses expressing sEng exhibit elevated plasma sEng levels, following a temporal pattern comparable to that seen in human preeclampsia, and show placental abnormalities resembling those resulting from inadequate spiral artery remodeling seen in preeclampsia [90]. Ex vivo and in vitro experiments, performed in human placental explants and a human trophoblast cell line, showed that sEng disrupts trophoblast invasion and pseudovasculogenesis, through which cytotrophoblasts transition from an epithelial to an endothelial-like state; both processes are associated with spiral artery remodeling [90]. Several animal models of preeclampsia have demonstrated a correlation between elevated sEng levels and hypertension, a hallmark of preeclampsia [88–90]. This finding has prompted the study of the molecular mechanisms involved in the pathogenic role of sEng [91]. Proteomic and transcriptomic analyses in human endothelial cells revealed that sEng induces the expression of BMP4. Moreover, transgenic mice expressing high plasma levels of human sEng showed enhanced circulating levels of BMP4 and upregulated BMP4 transcripts in the lungs, stomach, and duodenum compared with control animals. Following the mating of female wild-type mice with male mice producing elevated plasma levels of human sEng, hypertension developed 18 days postcoitus, coinciding with a rise in plasma BMP4 levels. Notably, noggin, a BMP4 inhibitor, abolished the rise in arterial pressure in mice expressing high levels of circulating human sEng. Furthermore, a positive correlation between sEng and BMP4 levels in circulation was observed in pregnant women, regardless of preeclampsia status. These findings are consistent with the hypothesis that BMP4 is a downstream mediator of sEng in the physiopathology of preeclampsia [91]. Podocyturia, which precedes proteinuria, is detected in individuals with preeclampsia [92]. In the kidney, podocytes regulate glomerular protein filtration, while remaining anchored to the glomerular basement membrane (GBM) through various β1 integrins. Remarkably, the glomerular podocyte count in transgenic mice overexpressing human sEng was was significantly lower than in wild-type mice, whereas podocytes and sEng were observed in the urine of mice overexpressing human sEng but not in that of wild-type (WT) mice. Accordingly, it was postulated that sEng, by targeting β1 integrins, can detach podocytes from the GBM, contributing to podocyturia [36].

### Cancer

Endoglin is expressed not only by certain tumor cells but also by various cell types within the tumor microenvironment, including endothelial cells of the tumor vasculature, cancer-associated fibroblasts (CAFs), tumor-associated macrophages (TAMs), and tumor-associated lymphocytes (TALs). This tumor-associated expression pattern has prompted a wide range of investigative efforts to study endoglin as a prognostic, imaging, and potential therapeutic target in cancer [38, 50, 78, 93, 94]. Thus, increased levels of endoglin in some cancer cells has been linked to tumor progression, malignant phenotype and metastasis; among others, in breast carcinoma, melanoma, or Ewing sarcoma [38, 66, 95, 96]. As endoglin is abundantly expressed by tumor-associated vascular endothelium, monoclonal antibodies to endoglin have been used to treat cancer and to visualize tumor angiogenesis using imaging techniques [75, 94, 97, 98]. Moreover, the expression of endoglin in CAFs, TAMs, and TALs has also been targeted in preclinical studies and clinical trials, while the existing body of research is still small and occasionally conflicting [50].

## Cadherin-5, −6, and −17

### Protein structure

**Fig. 4:**
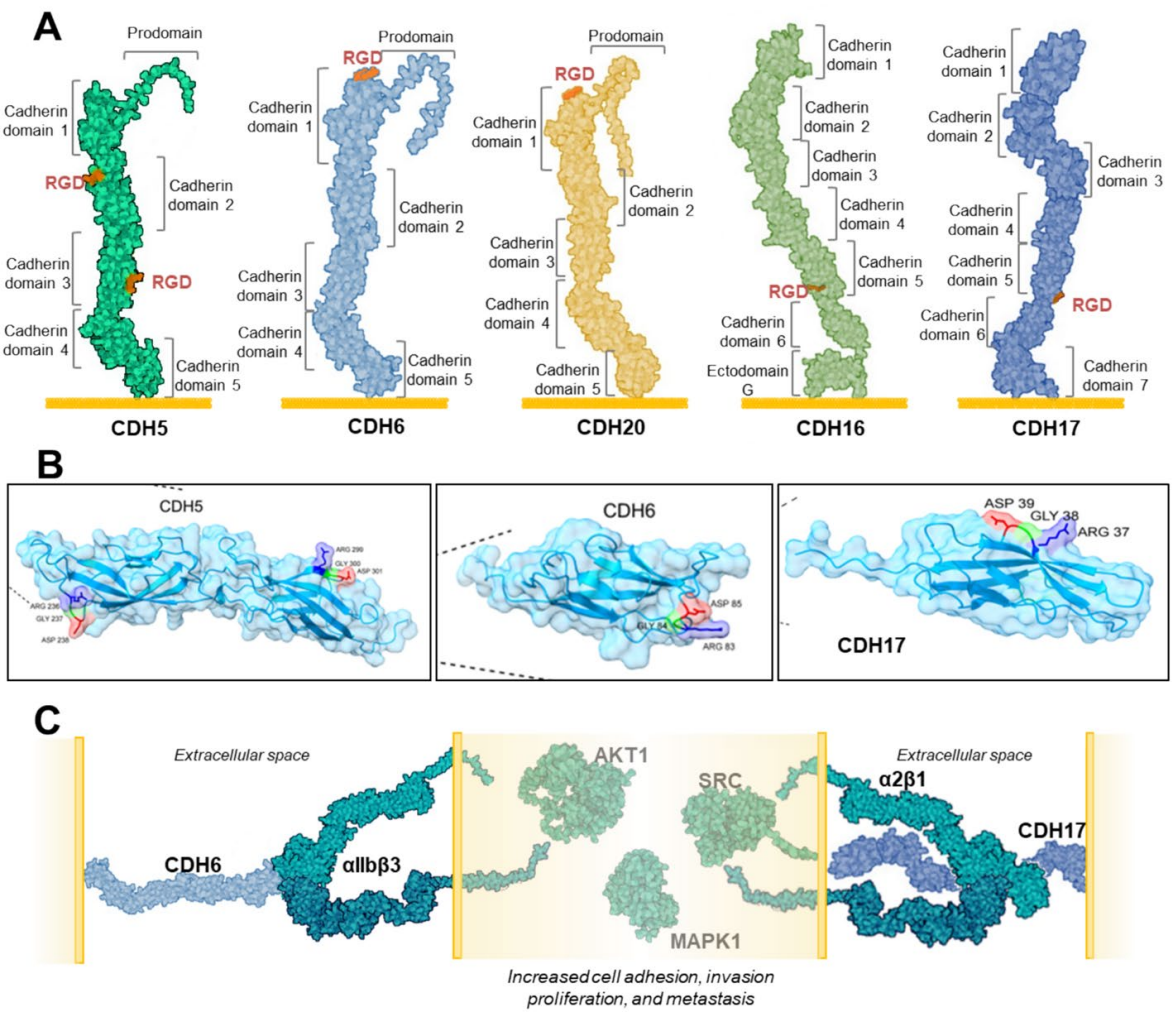
Structure and function of RGD cadherins. **A** Structural representation of the structure of the five RGD motif-containing cadherins. **B** Models show the 3D structure of the domains containing the RGD motifs: cadherin domains (CD) 2 and 3 in CDH5, CD1 in CDH6, and CD6 in CDH17. **C** The interaction of RGD-cadherins with integrins activates signaling pathway components, resulting in enhanced cell migration, invasion, and proliferation. Created with BioRender.com

The cadherin family in humans comprises 29 members, all sharing multiple extracellular cadherin (EC) domains. These domains facilitate homotypic adhesion in a calcium ion-dependent manner, pivotal for cell–cell adhesion during vertebrate embryo morphogenesis and development [99] and adult epithelial tissue maintenance [100]. Among these, five cadherins (CDH5, CDH6, CDH20, CDH16, and CDH17) feature RGD motifs within their ectodomains and can be collectively termed RGD cadherins. The first three belong to the type II cadherin subfamily, while CDH16 and CDH17 are part of the 7D subfamily [101]. Type II cadherins exhibit the classical cadherin structure: five EC (extracellular) domains followed by a transmembrane region and a relatively long cytoplasmic segment (152–182 amino acids) containing binding motifs for catenins (β-catenin and p120-catenin) that connect to the actin cytoskeleton via α-catenin. Conversely, 7D cadherins possess seven EC domains, stemming from a duplication event during early vertebrate evolution. Additionally, they feature a short cytoplasmic tail (21–23 amino acids) devoid of catenin binding motifs. The RGD motifs are located within various EC domains of these cadherins: EC6 of CDH17, EC5 of CDH16, EC1 of both CDH6 and CDH20, and both EC2 and EC3 of CDH5 (Fig. 4).

Consequently, the RGD-surrounding sequences differ, except for the closely related CDH6 and CDH20. However, the sequences preceding the RGD motif of CDH17 and both RGD motifs of CDH5 are characterized by a polar residue (Ser or Gln) at positions −2 or −3 and Leu at position −1, whereas CDH6 and CDH20 exhibit the sequences DQD and DMD, respectively, at such positions. Despite these differences, peptides harboring the RGD motif and flanking amino acid regions from these cadherins are recognized by integrins, except for the RGD motif of CDH16, which possesses a distinct preceding sequence (RAI) with a positively charged amino acid. The evolutionary origins of these motifs also vary [102]. The RGD motif in CDH6 is present in all vertebrates, although it changes to KGD in most Sauropsida (reptiles and birds). The RGD in CDH20 is only found in Amniota (reptiles, birds, and mammals). The RGD in EC2 of CDH5 arose independently in birds and mammals, while the RGD in EC3 is exclusive to primates. Also restricted to primates is the RGD in CDH16. Finally, the RGD motif in CDH17 is present in most mammals (but absent in rodents and several carnivores). Here, we will focus on CDH5, CDH6, and CDH17 owing to their integrin-binding capacity [102], as the CDH16 RGD motif appears nonfunctional in terms of integrin binding [103], and the potential activity of the CDH20 RGD motif remains entirely unexplored.

### Tissue distribution and functional activities

CDH5, also referred to as vascular endothelial cadherin (VE-cadherin) and CD144, predominantly resides within the adherens junctions of endothelial cells found in blood and lymphatic vessels. As a vital constituent of cell–cell connections, CDH5 assumes a pivotal role in maintaining endothelial cohesion and permeability regulation [104]. Its indispensability in vascular development is highlighted by the fact that knockout (KO) mice fail to survive embryogenesis [105]. Specifically, CDH5 facilitates the stabilization of nascent vessels, safeguarding them against disintegration [106]. Moreover, CDH5 expression extends to syncytiotrophoblasts, the cells forming the placental barrier between maternal and fetal bloodstreams, where it performs a role similar to that in endothelial cells. Furthermore, CDH5 is present in adipocytes and late spermatids, areas where its functionality remains largely unexplored.

CDH6, also termed kidney cadherin (K-cadherin), primarily appears in the fetal kidney, orchestrating the epithelial differentiation within this organ [107]. However, in the mature kidney, CDH6 is substituted by CDH16 (kidney-Specific (KSp)-cadherin), showing a modest expression in proximal tubular cells. While exhibiting faint expression levels, CDH6 is detectable in selected excitatory neurons and glial cells in the brain, smooth muscle cells across various tissues, and cholangiocytes within the bile duct. The functional roles of CDH6 in these cell types remain largely unexplored. By contrast, the functional significance of CDH6 in platelets has been uncovered. CDH6 surface expression increases in thrombin-activated platelets, serving as an RGD ligand for αIIbβ3 integrin and thereby facilitating platelet aggregation [108].

The expression pattern of CDH17, also termed liver intestine cadherin (LI-cadherin) or human intestinal peptide-associated transporter (HPT-1), is predominantly confined to colorectal enterocytes, Paneth cells, and goblet cells (see [26]] for a review). Additionally, minimal levels of this cadherin are observable in the epithelial cells lining the esophagus, stomach, and pancreatic ducts. Situated on the basolateral surface of large intestinal cells, CDH17-mediated cell–cell adhesion contributes, in conjunction with E-cadherin (CDH1), to the maintenance of epithelial integrity [109]. Remarkably, CDH17 knockout mice exhibited viability without histological alterations in the intestinal mucosa but increased the permeability of the epithelium [110], in stark contrast to intestine-specific E-cadherin knockout mice, which manifest severe defects in intestinal morphogenesis and homeostasis [111]. Furthermore, CDH17 acts as a proton-dependent peptide transporter, facilitating water absorption, and regulates intestinal permeability [112]. Moreover, CDH17 is expressed in proB cells within the bone marrow and immature B cells within the spleen, contributing to the developmental trajectory of B lymphocytes during these stages [113].

### Role in RGD-dependent cell adhesion

The RGD motifs within CDH5 and CDH17 share similar specificity, likely due to the similitude in their flanking sequences. Despite αV integrin being considered the primary RGD receptor in many cells, cadherin RGD motifs do not act as ligands for this integrin. Instead, cadherin RGD motifs bind to α2β1 integrin, typically identified as a collagen receptor but not recognized as an RGD receptor [24, 114]. Moreover, the collagen motif (GFOGER) and the RGD motif are recognized in different locations within the integrin alpha chains. Whereas the collagen motif binds to the α-I domain [115], the RGD motif binds to the β-propeller [116], suggesting that α2β1 integrin utilizes distinct regions to bind both ligands. Therefore, the conventional classification of integrins as (i) RGD receptors, (ii) LDV receptors, (iii) collagen receptors, and (iv) laminin receptors [1] appears overly restrictive, as many integrins exhibit multiple binding capacities; for instance, α10β1 binds to both collagens and laminins [117] and α2β1 may work as collagen and RGD receptor [26]. The interaction of α2β1 integrin with CDH17 or CDH5 promotes a high-affinity integrin conformation, as silencing of these cadherins reduces β1 integrin levels in its high-affinity state. Conversely, exposure to peptides containing cadherin RGD motifs increases the high-affinity conformation of β1 integrin [24, 114]. Subsequently, the increase in active α2β1 integrin enhances the binding to collagen. The cadherin–integrin interaction triggers the activation of various signaling mediators downstream of the integrin, such as Src, FAK, AKT, and MAP kinases [25]. Immunoprecipitation assays of CDH5 suggest that the cadherin remains attached to the integrin during activation of these signaling pathways, as these mediators are also present in the CDH5 immunoprecipitates [114]. CDH6 RGD predominantly binds to αIIbβ3 integrin, a canonical RGD receptor [108]. However, in the absence of αIIbβ3 integrin, CDH6 also binds to α2β1 integrin [118]. The interaction of CDH6 with these receptors promotes the high-affinity conformation of the integrins and activates signaling pathways. Remarkably, there is an integrin crosstalk in ovarian and renal cancer cells expressing both αIIbβ3 and α2β1 integrins, where CDH6-induced activation of αIIbβ3 subsequently provokes an inside-out activation of α2β1 [118]. This was the first report of integrin crosstalk in solid cancer cells. Owing to the limited expression of αIIbβ3 integrin, the potential interaction with the CDH17 RGD motif has only recently been investigated. CDH17 can bind to both αIIbβ3 and α2β1 integrins without a clear preference, suggesting similar affinities for both receptors [118]. The potential binding of CDH5 to αIIbβ3 integrin seems plausible but remains unexplored. Additionally, CDH17 binds to the desmosomal cadherin desmocollin-1, through a different motif, promoting a more invasive phenotype in colorectal cancer cells that have undergone the epithelial–mesenchymal transition [119]. Shedding of CDH17 ectodomain has been detected in serum of patients with colorectal cancer, but the potential role of this soluble form in cadherin–cadherin or cadherin–integrin interactions has not been characterized [102].

### Pathophysiological involvement and implications

The majority of investigations on cadherin RGD motifs have been conducted in cancer cell lines. Hence, we initially delineate the RGD activities of cadherins in pathological contexts. CDH17 is highly expressed in a significant proportion of colorectal tumors and other gastrointestinal malignancies, including gastric [120], pancreatic [121], liver [122], neuroendocrine [123], and carcinoid tumors. Additionally, CDH17 is detected in some mucinous ovarian tumors, cervical adenocarcinomas, biliopancreatic adenocarcinomas, and other neuroendocrine neoplasms [124]. CDH5 is linked to vasculogenic mimicry (the capacity of cancer cells to form new blood vessel-like channels) in uveal melanoma [125, 126], glioblastoma stem-like cells [127], and other cancers [128]. CDH5 exhibits a broader overexpression profile in cancer, being commonly observed in metastatic breast cancer [129], lung cancer [130], melanomas [131], gastric cancer [132], and pancreatic neuroendocrine tumors [133]. CDH6 is predominantly detected in renal and thyroid cancers, with additional expression in gastric, pancreatic, and ovarian tumors [134–136]. The widespread expression of RGD cadherins in various cancers implies that their presence confers some advantage to tumor cells, fostering cancer progression. Indeed, RGD cadherins facilitate the activation of α2β1 or αIIbβ3 integrins in cancer cells [114, 118]. This activation or enhancement in affinity augments cellular adhesive capabilities and triggers signaling pathways, culminating in increased cell proliferation and migration [24, 118]. Furthermore, CDH5 expression facilitates cancer cell transmigration through endothelial barriers and promotes vasculogenic mimicry [114], pivotal events for cancer cell progression and metastatic colonization of distant organs.

The expression of CDH17 typically diminishes in the initial stages of colorectal cancer compared with normal mucosa and increases in advanced cancer stages, particularly in liver metastasis [137]. Likewise, CDH6 shows increased expression in metastatic renal cancer relative to primary tumors, which correlates with more αIIbβ3 expression [118]. High expression of CDH5 acts as unfavorable prognostic indicator in melanoma and breast cancer, while CDH6 expression is associated with poor outcome in renal and ovarian cancer [114, 118]. Moreover, high CDH17 expression inversely correlates with survival in patients with colon cancer [24]. These cadherin-mediated effects mainly involve the RGD motifs, as evidenced by the therapeutic efficacy of anti-RGD cadherin monoclonal antibodies targeting these sites, which protect mice from experimental metastasis development in liver and lungs. The efficacy was demonstrated using either colorectal cancer cells expressing CDH17 or CDH5-expressing melanoma cells, owing to the high homology of the RGD flanking sequences [103]. In spite of the evident variations in the RGD motif surrounding sequences (Supplementary Fig. 3D), anti-RGD cadherin monoclonal antibodies were also effective in blocking CDH6 interaction with integrins [103]. These antibodies also hinder signaling pathway activation and integrin affinity enhancement. Conversely, surface expression of RGD cadherins on circulating cancer cells, particularly CDH6, may facilitate platelet recruitment via αIIbβ3 integrin. Platelets play a key role in the hematogenous dissemination of cancer cells, fostering immune evasion and cellular arrest in distant organs [138].

As mentioned before, the potential physiological roles of RGD motifs in cadherins have been scarcely studied, except for the interaction of αIIbβ3 integrin with the CDH6 RGD motif in platelets. Activation of platelets provokes the exposition of CDH6 on the cell membrane to enable the interaction with αIIbβ3 integrin, thus contributing to platelet aggregation, a key event against bleeding from blood vessel injuries. This biological process may explain the ancient evolutionary origin of the RGD motif in CDH6. In addition, the CDH6/αIIbβ3 integrin association can also contribute to thrombus formation in several pathologies [108].

Overall, RGD cadherins are expressed in tissues with high regeneration rates and subjected to abrasion or pressure, as CDH17 in colon, CDH5 in endothelium, or CDH6 in the proximal tubules of kidneys. In these tissues, RGD cadherins can promote stronger adhesion to basal membrane and increased proliferation/survival through the activation of integrins. Thus, CDH5 promotes endothelial cell survival, proliferation, and migration and, in coordination with integrins, participates in vascular network construction during angiogenesis [139]. Furthermore, CDH17 is expressed in hepatocytes located near necrotic zones in liver [140]. Such CDH17-expressing hepatocytes show increased proliferation, required to repopulate the injured region. Of note, whereas the α2β1 integrin has a widespread expression, the αIIbβ3 integrin expression is limited to a select number of cell types, which also express CDH6 (as oligodendrocyte progenitors) or CDH5 (as late spermatids and lymphatic endothelial cells), or to several cancer types. This coordinated expression suggests that αIIbβ3 integrin associates with cadherin RGD motifs, in addition to other counter-receptors (i.e., endoglin), to fulfill its physiological role. In summary, the RGD motifs in cadherins, through their interplay with integrins, contribute to various aspects of animal physiology, such as epithelium cohesion and endurance, tissue regeneration, and platelet aggregation. Nonetheless, these motifs are also critically involved in cancer progression and metastatic spread, rendering them attractive targets for developing novel therapeutic strategies across various cancer types..

## ADAM15

### Protein structure

**Fig. 5:**
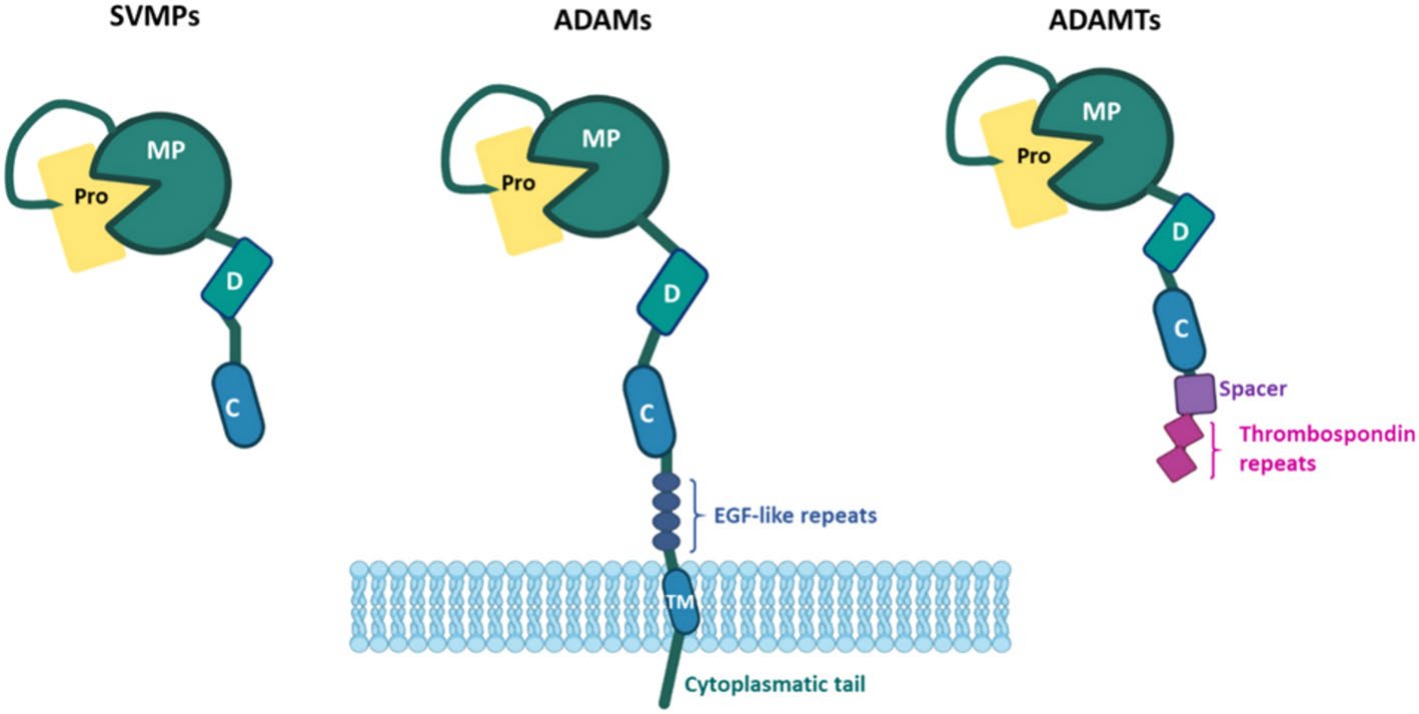
General structure of a disintegrin and metalloprotease (ADAMs) (central), snake venom metalloproteases (SVMPs) (left) and a disintegrin and metalloprotease with thrombospondin motif (ADAMTSs) (right). Pro, prodomain; MP, metalloprotease/catalytic domain; D, disintegrin (-like) domain; C, cysteine-rich domain; TM, transmembrane domain. Created with BioRender.com

ADAMs constitute a family of type-I transmembrane proteins with a multidomain modular structure that typically comprises a large extracellular region encompassing (from N- to C-terminus) the prodomain and catalytic- (also termed metalloproteinase domain), disintegrin-, cysteine-rich- and EGF-like-repeats domains, followed by single transmembrane and intracytoplasmic domains (Fig. 5) (reviewed in [141–145]). The name ADAM (a disintegrin and metalloproteinase) denotes the defining presence of the disintegrin and metalloproteinase domains in these proteins, although these two domains are also present in other closely related metalloproteinase families, such as the ADAMs containing thrombospondin motifs (ADAMTSs) and snake venom metalloproteinases (SVMPs). In fact, ADAMs, ADAMTSs, and SVMPs collectively form the adamalysin subfamily within the zinc-based metzincin subgroup of proteases, which also includes the structurally related MMPs (reviewed in [141, 144, 145]).

The human genome contains 22 different ADAMs, of which only 12 possess the highly conserved catalytic Zn-binding motif (**H**EXG**H**XXGXX**H**D) and the ensuing characteristic methionine-turn of metzincin proteases in the active site of the metalloproteinase domain, indicating that only these 12 members (ADAM-8, −9, −10, −12, −15, −17, −19, −20, −21, −28, −30, and −33) are actually proteolytically active. By contrast, the other ten ADAMs members lack either the conserved Zn-binding motif or the methionine-turn required for proteolytic activity in their catalytic domains [reviewed in [141, 146]].

ADAMs are synthesized as inactive proproteins (zymogens) that contain the prodomain, then translocate to the endoplasmic reticulum and further mature in the Golgi compartment. The prodomain plays important functions during the maturation of ADAMs in the late Golgi. By functioning as an intramolecular chaperone, the prodomain assists in proper protein folding and disulphide bonding formation, while also maintaining ADAMs in an inactive state during maturation through a “cysteine-switch” mechanism that inhibits its catalytic activity [142, 147–149]. The ADAM maturation process further involves extensive complex glycosylation and removal at a later state of the prodomain from the ADAMs zymogens. Prodomain removal is carried out either by furin (or by furin-like proprotein convertases) (in ADAM-9, −10, −12, and −17) or through autocatalytical removal (in ADAM-8 and −28) in the trans-Golgi network, which finally renders the catalytically active mature metalloproteinases [142, 147, 150, 151].

**Fig. 6:**
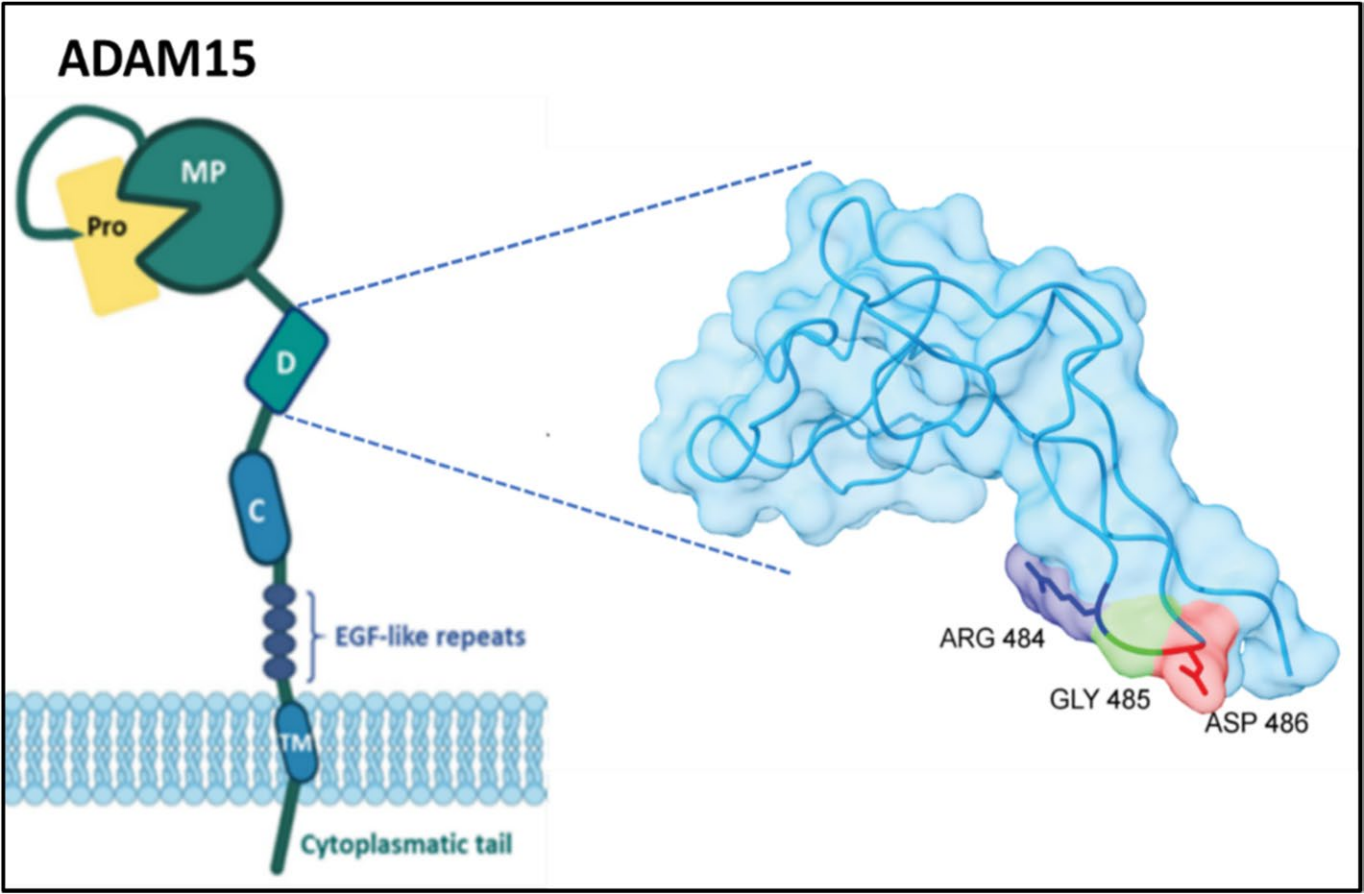
Schematic representation of ADAM15 structure. The different domains of ADAM15 are indicated: prodomain (Pro), metalloprotease (MP), disintegrin (D), cysteine-rich (C), EGF-like repeats, transmembrane (TM), and cytoplasmic tail. The model on the right shows the 3D structure of the disintegrin domain and the location of the RGD motif. Created with BioRender.com

The disintegrin domains present in ADAMs are structurally related to the snake venom disintegrins (SVDs), a family of small proteins that competitively inhibit integrin-mediated adhesion of platelets to their ligand fibrinogen, thus causing hemorrhagic effects. Integrin inhibition by SVDs is due to the presence of an RGD (or the related KGD) sequence at the end of a characteristic extended loop in these small proteins, termed the disintegrin loop. However, the analogous disintegrin domains in ADAMs (and also in ADAMTSs and SVMPs) should more correctly be called “disintegrin like” because, with the only exception of human ADAM15, they lack the defining RGD (or KGD) sequence in their disintegrin loops [141, 152–154]. The initial identification some thirty years ago of these “disintegrin-like” domains in ADAMs led to the hypothesis that these domains were recognized and bound by integrins, in a similar way to the disintegrin domains of SVDs [155]. Since then, many studies have demonstrated that the “disintegrin-like” domains of ADAMs are indeed specifically recognized and bound by integrins and a consensus sequence (CRXXXXXCDXXEXC) **XCD** has been identified in most of their disintegrin loops, except in ADAM-10 and ADAM-17 members [141, 153, 154]. ADAM10 and ADAM17 are actually the two best characterized members of the ADAMs family, and numerous excellent reviews covering different aspects of the biology of these two molecules have been published [145, 156–158]. ADAM10 and ADAM17 are responsible for the cleavage and release of the extracellular portion from numerous cell surface proteins (“ectodomain shedding”) and play an essential role in development and in many other crucial physiological and pathological processes [141, 145, 159–161]. Structurally, ADAM10 and ADAM17 are also atypical ADAMs members as the cysteine-rich and EGF-like-repeats domains present in the rest of ADAMs are substituted by a distinct “membrane proximal domain,” involved in substrate recognition and shedding (reviewed in [156]). As ADAM15 is the only member that actually contains the RGD motif in its disintegrin loop, in this review, we will only consider this member as a transmembrane RGD-based integrin counter-receptor and leave out the rest of ADAMs (Fig. 6).

### Tissue distribution and functional activities

In contrast to many ADAMs (including ADAM-2, −7, −18, −20, −21, −29, and −30) that are expressed primarily in the testis, where they play different roles in spermatogenesis and sperm function (reviewed in [141]), ADAM15 is abundantly expressed in vascular cells, including smooth muscle and endothelial cells, and has been involved in vascular pathologies, including atherosclerosis and pathological neovascularization [162–164]. ADAM15 is expressed also in intestinal epithelial cells, where it plays a major role in intestinal inflammation, epithelial wound healing and mucosal remodeling in inflammatory bowel disease [165–167]. Of note, ADAM15 expression is frequently dysregulated (either overexpressed or downmodulated) in many types of cancer relative to the respective benign tissue counterparts [153, 168]. In this regard, the involvement of ADAM15 in cancer progression and metastasis processes has been reported for colorectal, breast, bladder and prostate adenocarcinomas [169, 170].

The human ADAM15 gene spans 11,367-bp and contains 23 exons and 22 introns. The exons 19–21 are used alternatively in different human tissues [153, 171]. Altered ADAM15 expression has been associated with various human diseases, including cancers, cardiovascular disease, atherosclerosis, and arthritis [153, 168, 172–175]. In cancer, ADAM15 has been implicated in tumor growth, angiogenesis and metastasis [164, 168, 176]. It has been shown that, in addition to transcriptional regulation of ADAM15 expression, alternative splicing of ADAM15 transcripts is also dysregulated in human cancer cells [177]. In this regard, multiple ADAM15 isoforms containing different Src homology 3 (SH3) domain binding sites in their intracytoplasmic tails can be generated through alternative mRNA splicing in different tissues [178]. This alternative use of ADAM15 exons profoundly influences selection of SH3-containing cellular partner proteins, thus providing a versatile mechanism for regulation of cellular functions, which could explain the association found for several (but not all) ADAM15 isoforms with cancer-related processes [178].

### Role in RGD-dependent cell adhesion

As mentioned above, human ADAM15 is the only member of the ADAMs family that possesses an RGD integrin-binding sequence in its disintegrin-like loop, and, for this reason, ADAM15 is also named metargidin (metalloprotease-RGD-disintegrin protein) [153, 179]. While this RGD motif is involved in the binding of ADAM15 to integrins αvβ3 and α5β1 integrins, ADAM15 has been shown to bind to integrin α9β3 in an RGD-independent manner [180]. Several functional activities attributed to ADAM15 are primarily dependent on its interaction with these integrins. In this regard, the interaction of ADAM15 with integrin αvβ3 has been shown to influence non-small-cell lung cancer (NSCLC) proliferation and metastasis through focal adhesion kinase (FAK) activation and signaling [181]. The functional implication of ADAM15 in cell migration has also been shown, but in this case, it seems to be rather dependent on ECM remodeling carried out by its active metalloproteinase catalytic domain through the cleavage of type IV collagen and gelatin [182]. As discussed below, the ADAM15-dependent regulation of cell migration also has important repercussions in cancer development and progression. ADAM15 itself is also involved in cell signaling through different motifs present in its intracytoplasmic tail, including: (i) proline-rich sequences that offer sites for interaction with Src-homology SH3 domain-containing signaling proteins; (ii) tyrosine phosphorylation sites that act as ligands for SH2 domain-containing signaling proteins; and (iii) serine and threonine phosphorylation sites. In this regard, it has been shown that several Src-family tyrosine kinases (including Lck, Hck, Fyn, Abl, and Src) as well as the Grb2 adaptor can associate specifically in a phosphorylation-dependent manner with the cytoplasmic tail of ADAM15 in hematopoietic cells, supporting a role for ADAM15 in cell signaling [183].

### Pathophysiological involvement and implications

ADAM15 is abundantly expressed in vascular cells, including endothelial and smooth muscle cells (SMCs), and is implicated in vascular pathologies, including atherosclerosis and pathological angiogenesis [162–164]. ADAM15, together with its binding partners, integrins α5β1 and αVβ3 [184, 185], have been found to be upregulated in atherosclerotic arterial areas in comparison with normal samples [162], which points to their collective involvement in atherosclerosis. It has been proposed that ADAM15 might act as a regulator of integrin-ECM interactions and that its increased expression in atherosclerotic lesions could occur in response to the upregulated expression of its α5β1 and αVβ3 integrin partners [reviewed in [166]]. ADAM15 has been shown to stimulate the migration of vascular endothelial cells and smooth muscle cells (SMCs) through different mechanisms, including its RGD-dependent association with integrins α5β1 and αVβ3 and its ability to cleave ECM proteins (such as gelatin and type IV collagen) through its metalloprotease activity. The ADAM15-stimulated migration of endothelial cells and SMCs may contribute to angiogenesis, to tissue remodeling, and to plaque formation in atherosclerosis [166].

Platelets are involved in different stages of atherosclerosis by contributing to the early recruitment of inflammatory leukocytes, acting as a bridge between endothelial cells and leukocytes and, at a later stage, by provoking thrombosis following the rupture of atherosclerotic plaques [186, 187]. The RGD sequence in the disintegrin domain of ADAM15 on endothelial cells can be recognized by platelet αIIbβ3 integrin, and these ADAM15/αIIbβ3 interactions have been shown to mediate the adhesion of platelets to endothelium [188]. Furthermore, it has been shown that the binding of platelet αIIbβ3 integrin to endothelial ADAM15 results in activation and recruitment of additional platelets, which leads to thrombus formation, underscoring the role of ADAM15 in atherosclerosis [166].

The involvement of ADAM15 in physiological and pathological angiogenesis has been investigated by Horiuchi et al. in mice with targeted deletion of ADAM15 (*Adam15*^−/−^ mice) [164]. Although the expression of ADAM15 in vascular cells and endocardium is significant in wild-type mice, no major developmental or pathological alterations were evident in *Adam15*^−/−^ mice, indicating that ADAM15 is not required for physiological angiogenesis development or adult vascular homeostasis. By contrast, the involvement of ADAM15 in pathological neovascularization was demonstrated in two experimental models of angiogenesis. In a mouse model for retinopathy induced by changes in oxygen concentrations, *Adam15*^−/−^ mice displayed very reduced neovascularization compared with wild-type controls. In addition, in a model of melanoma angiogenesis, the tumors formed by implanted melanoma cells were significantly smaller in *Adam15*^−/−^ mice than in wild-type controls. These authors concluded that ADAM15 could be a novel target for the design of inhibitors of pathological neovascularization.

In addition to the roles played by ADAM15 in atherosclerosis and pathological angiogenesis, accumulating evidence indicates that ADAM15 might be involved in other inflammatory disorders, such as rheumatoid arthritis (RA) and inflammatory bowel disease (IBD) (reviewed in [166]). Although most reports indicate a role for ADAM15 in these pathologies through its metalloproteinase catalytic activity and ECM degradation, additional roles of ADAM15 as an adhesion molecule involved in the modulation of cell–cell and/or cell–ECM interactions cannot be ruled out. In this regard, synovial lining cells in RA express both ADAM15 [172, 189] and integrins α5β1 and αVβ3 [190, 191], which indicates a potential role for ADAM15 as a regulator of cell adhesion through these integrins in RA. Likewise, ADAM15 is expressed by different cell types both in the normal and inflamed intestine (intestinal epithelial cells [IECs], pericryptic myofibroblasts, SMCs, and polymorphonuclear and mononuclear cells). Interestingly, ADAM15 frequently colocalizes with integrins α5β1 and αVβ3 and collagen IV on these cells. This coexpression pattern has potential consequences in the regulation of IECs and pericryptic myofibroblast migration and differentiation, as well as of homotypic and heteotypic cell–cell adhesion phenomena with implication in the pathogenesis of IBD [166, 167].

As indicated above, ADAM15 expression is frequently dysregulated in many types of cancer in comparison with the respective normal tissues [168], and a functional involvement of ADAM15 in cancer progression and metastasis has been proposed for colorectal, breast, bladder, and prostate adenocarcinomas [143, 169, 170]. ADAM15 may exert a dual function in metastasis by promoting the detachment of cells from ECM with its disintegrin domain and by degrading the ECM with its catalytic domain. In most cases however, ADAM15 involvement in cancer progression seems to depend fundamentally on its metalloproteinase activity rather than on its RGD-based adhesive properties.

Maretzky et al. reported that a splice variant of ADAM15 (named ADAM15B) that contains an inserted cytoplasmic Src-binding site is associated with unfavorable outcomes in breast cancer [192]. These authors showed that ADAM15B displays a significantly enhanced metalloprotease activity responsible for the shedding of ectodomains from transmembrane ADAM15 transmembrane substrates, including the shedding of fibroblast growth factor receptor 2 (FGFR2), which is involved in the development of breast cancer. The enhanced shedding of FGFR2 by ADAM15B was abolished in *Src*^−/−^ cells and by the use of Src inhibitors, demonstrating the specific involvement of Src tyrosine kinase. By mutating each of the four tyrosine residues in the cytoplasmic domain of ADAM15B, they found that phosphorylation of the third tyrosine (Y-375) was crucial for activation of ADAM15B by Src. These authors concluded that the enhanced cell surface shedding of FGFR2 carried out by the increased catalytic activity of ADAM15B was involved in the progression of breast cancer and suggested that inhibitors of ADAM15 or of the ADAM15B/Src interaction might be effective to treat patients with breast cancer with dysregulated ADAM15.

The group of Mark L. Day found that ADAM15 transcript and protein levels were significantly increased in breast and prostate cancer tissue specimens and that expression of this molecule correlated with disease progression and metastasis according to clinical parameters of predictive outcome for both prostate and breast cancers, evidenced by Gleason sum and angioinvasion, respectively [168]. This group postulated that ADAM15 can promote prostate cancer progression through different mechanisms: (i) by disrupting cellular attachments to neighboring cells; (ii) by influencing cell signaling in an autocrine or paracrine fashion through the shedding of membrane-bound growth factors; and (iii) by disrupting cellular interactions with the extracellular matrix and basement membrane [169].

Lorenzatti Hiles et al. found that ADAM15 expression is increased in invasive and metastatic bladder tumors, while low grade and noninvasive bladder cancer showed negative or low expression of ADAM15 [193]. They also showed that the knockdown of ADAM15 mRNA expression significantly reduced the migration and invasive capacity of bladder tumor cells. Moreover, in a xenograft model of human bladder cancer the growth of ADAM15 knockdown cells was inhibited by 45% compared with wild-type control cells. These authors also carried out the structural modeling of the catalytic domain of ADAM15, and subsequently designed a specific sulfonamide inhibitor of this metalloproteinase that in vitro reduced significantly the viability of bladder cancer cells and was also effective in human bladder cancer xenografts. These results supported the role of ADAM15 in human bladder cancer invasion and pointed to the catalytic domain of ADAM15 as a therapeutic target in this pathology.

Toquet et al. analyzed the expression of ADAM15 as well as promoter methylation and microsatellite instability status in samples of 94 colorectal carcinomas, categorized according to the World Health Organization classification. These authors found a reduced expression of ADAM15 (both at protein and mRNA levels) without promoter methylation in 36% of colorectal carcinomas, which was associated with histologically poorly differentiated carcinomas [170]. Of note, the reduction in ADAM15 expression was also associated with the acquisition of integrin α5β1 and downregulation of integrin α3β1 integrin and E-cadherin by cancer cells, which are hallmarks of the epithelial–mesenchymal transition (EMT) process that characterizes colon cancer progression and poor prognosis.

## L1CAM

### Protein structure

**Fig. 7:**
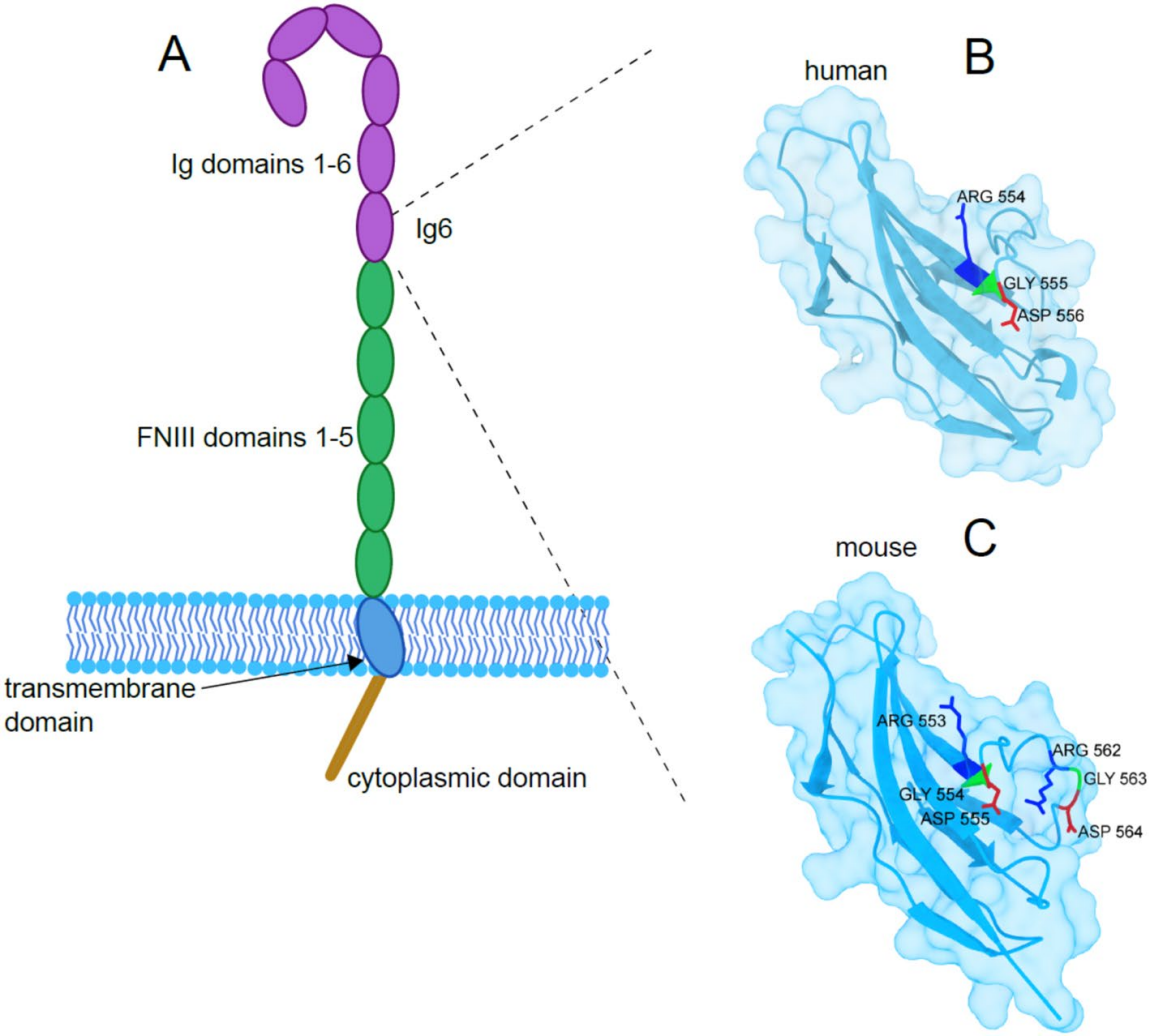
Schematic representation of the L1CAM protein, embedded in the cell membrane (**A**). The protein is composed of: immunoglobulin-like (Ig) domains 1–6, fibronectin type III (FNIII) repeats 1–5, a transmembrane domain, and a cytoplasmatic domain. (**B**) AlphaFold-predicted structure of the human Ig6 domain, highlighting the RGD motif located at amino acid positions 554–556. (**C**) AlphaFold-predicted structure of the mouse Ig6 domain, highlighting two RGD motifs: at positions 553–555 and 562–564

L1CAM (CD171) is a transmembrane glycoprotein of the immunoglobulin superfamily (Ig-SF) with a molecular wight of 200–220 kDa. L1CAM encompasses six Ig-like domains and five fibronectin type III repeats, followed by a single transmembrane region and a highly conserved intracytoplasmic tail (Fig. 7) [194]. L1CAM can bind to itself (L1CAM–L1CAM homophilic interactions) or heterophilically to other neural cell adhesion molecules, integrins, CD24, neurocan, and neuropilin-1 [195, 196]. The cytoplasmic tail of L1CAM can interact with the cytoskeletal proteins ankyrin, actin, spectrin, and ERM (Ezrin, Radixin and Moesin) proteins [197, 198] and various other intracellular proteins [199, 200].

In the sixth Ig-domain, there are RGD-sites (Fig. 7, right) (one in human, two in mice) that support the binding to integrins such as α5β1, αvβ3, αIIbβ3, and αvβ5 [201, 202]. The first Ig domain can bind to the proteoglycan neurocan [203] and the VEGF-R2-coreceptor neuropilin-1 [204, 205]. Importantly, the ectodomain of full length L1CAM is subject to membrane proximal cleavage and is released (“shed”) by metalloproteinases such as ADAM10 and ADAM17 [206, 207], which generates a soluble form of about 200 kDa (sL1CAM) [208, 209]. L1CAM cleavage and release of the soluble molecule promotes cell migration, invasion, and protection from apoptosis of cancer cells in vitro [207, 210–212]. sL1CAM can be detected in serum and malignant ascites of patients with ovarian and endometrial cancer and of other cancer types [210, 213]. After ADAM-mediated cleavage the cytoplasmic portion is L1CAM is further processed by presenilins and β-secretase (BACE) [214, 215] and can enter the nucleus to mediate gene regulation [216, 217]. Additional proteinases that can cleave L1CAM have been identified [218] and a soluble form can also be generated by alternative splicing in endothelial cells [219].

### Tissue distribution and functional activities

L1CAM is predominantly expressed in neural tissue, but low levels of expression are present in certain epithelial and endothelial cells as well as in leukocytes [220]. L1CAM plays an essential role in the development of the nervous system by promoting neuronal migration, axon growth, and synapse formation [221, 222].

Different mutations in the L1CAM gene leading to malfunction of the protein have been observed in certain human neurodevelopmental disorders such as X-linked hydrocephalus, spastic paraplegia, and mental retardation [222]. Overall, several hundred mutations have been identified in the L1CAM gene [223].

L1CAM is also highly relevant in tumor progression. This molecule is highly expressed in a variety of human cancers, including colorectal, pancreatic, endometrial, and ovarian carcinomas, as well as neuroblastomas and melanomas [224–226]. High expression of L1CAM is associated with an increased grade of malignancy, epithelial–mesenchymal transition (EMT), poor patient prognosis, and worse response to chemotherapy (for review, see [224]). Recent work has also suggested that L1CAM upregulation marks metastasis-initiating cells in colorectal cancer [225] and defines a new cancer stem cell population in ovarian cancer [227].

### Role in RGD-dependent cell adhesion

When immobilized as a substrate, L1CAM supports cell adhesion and migration similar to ECM components, and this process can be blocked in the presence of antibodies to integrins [207, 228]. Mutations of the RGD site to RGE in mouse or human L1CAM abolished the capacity to serve as substrate [228]. Likewise, RGD-mutant L1CAM proteins had lost the ability to trigger cell migration on ECM components [207]. These data suggested that the RGD sites in L1CAM support the binding to integrins such as α5β1 or αvβ3. By contrast, when the indicator cells expressed themselves L1CAM, a homophilic binding was observed that was blocked by anti L1CAM antibodies [229]. This suggested that L1CAM could engage in two ways: homophilic L1CAM–L1CAM binding or L1CAM–integrin binding [230, 231]. It is conceivable that both ways of binding trigger distinct signaling pathways and drive various functions [211, 230].

### Pathophysiological involvement and implications

The knockdown of L1CAM in mice recapitulated its importance for neuronal development, as the brain defects of these mice were similar to humans with mutations in the L1CAM gene [232]. Attempts to better understand the role of RGD sites in the sixth Ig domain were also undertaken. [233]. Mice lacking this domain of L1CAM (L1-6D mice) lost homophilic binding (most likely due to altered domain alignment) and RGD-dependent L1CAM–integrin binding [233]. The ultrastructural analysis of sciatic nerves revealed unmyelinated axons that frequently detached at the edge of Schwann cells, and, in addition, naked axons were observed [234]. As previous work had shown that L1CAM on axons interacts with a heterophilic binding partner on Schwann cells, it was proposed that L1CAM on axons binds integrins on Schwann cells, resulting in interactions between axons and Schwann cells that are essential for ensheathment and myelination [234]. αv-integrins on Schwann cells are possibly involved [235].

### General discussion and concluding remarks

Integrin binding typically occurs through recognition of specific short peptide motifs containing an acidic residue (D or E) in the ligands. The two best characterized sequences recognized by numerous integrins on their ligands are the motifs RGD and LDV. However, many other motifs (including KGD, RTD, KQAGDV, DGEA, YGYYGDALR, FYFDLR, and GFOGER) have been identified in diverse integrin ligands as responsible for specific integrin recognition and binding [14, 19, 27]. Therefore, as more new integrin ligands are being discovered and characterized, it seems likely that the RGD- or LDV-based engagements may represent a minor subset of the entire spectrum of integrin–ligand interactions. Many classical integrin ligands are ECM proteins (fibronectin, laminin, collagens, vitronectin, fibrinogen, and so on) that contain RGD or LDV motifs, and mediate crucial cell–ECM adhesion phenomena. By contrast, many other integrin ligands are non-ECM proteins, including transmembrane proteins expressed on the surface of cells, also termed integrin counter-receptors or ICRs. These ICRs mediate pivotal cell–cell adhesion processes in vertebrates, such as leukocyte and cancer cell extravasation, cooperation between immune cells, and stem-cell homing. Noteworthly, the expression of non-ECM integrin ligands is not restricted to the eukaryotic cells of higher metazoans, as these proteins are found also on the surface of pathogenic and nonpathogenic fungal and prokaryotic cells as well as on viruses [22]. This review is focused on presenting and discussing the RGD-based interactions of integrins with a selection of transmembrane counter-receptors that are expressed on human cells and have crucial pathophysiological relevance. These RGD-containing integrin counter-receptors include the proteins endoglin, cadherin-5, −6, and −17, ADAM15, and L1CAM.

According to the UniProt protein database, there are 2352 human proteins that contain at least one RGD motif (7.22% of all human proteins), of which 367 are membrane proteins. We have selected six transmembrane RGD-containing proteins (endoglin, cadherin-5, −6, and −17, ADAM15, and L1CAM) out of those 367, because they are expressed on the cell surface membrane and are well-established counter-receptors for at least one member of the RGD-receptor group of integrins (Fig. 1), according to previous reports in literature. Here, we have grouped these transmembrane proteins under the newly coined acronym RGD-containing integrin counter-receptors (RGD-ICRs). Among the remaining 361 transmembrane RGD-containing proteins, their role as integrin counter-receptors has been poorly studied, but future investigations may serve to identify additional new members of this growing family of membrane-bound integrin ligands.

**Fig. 8:**
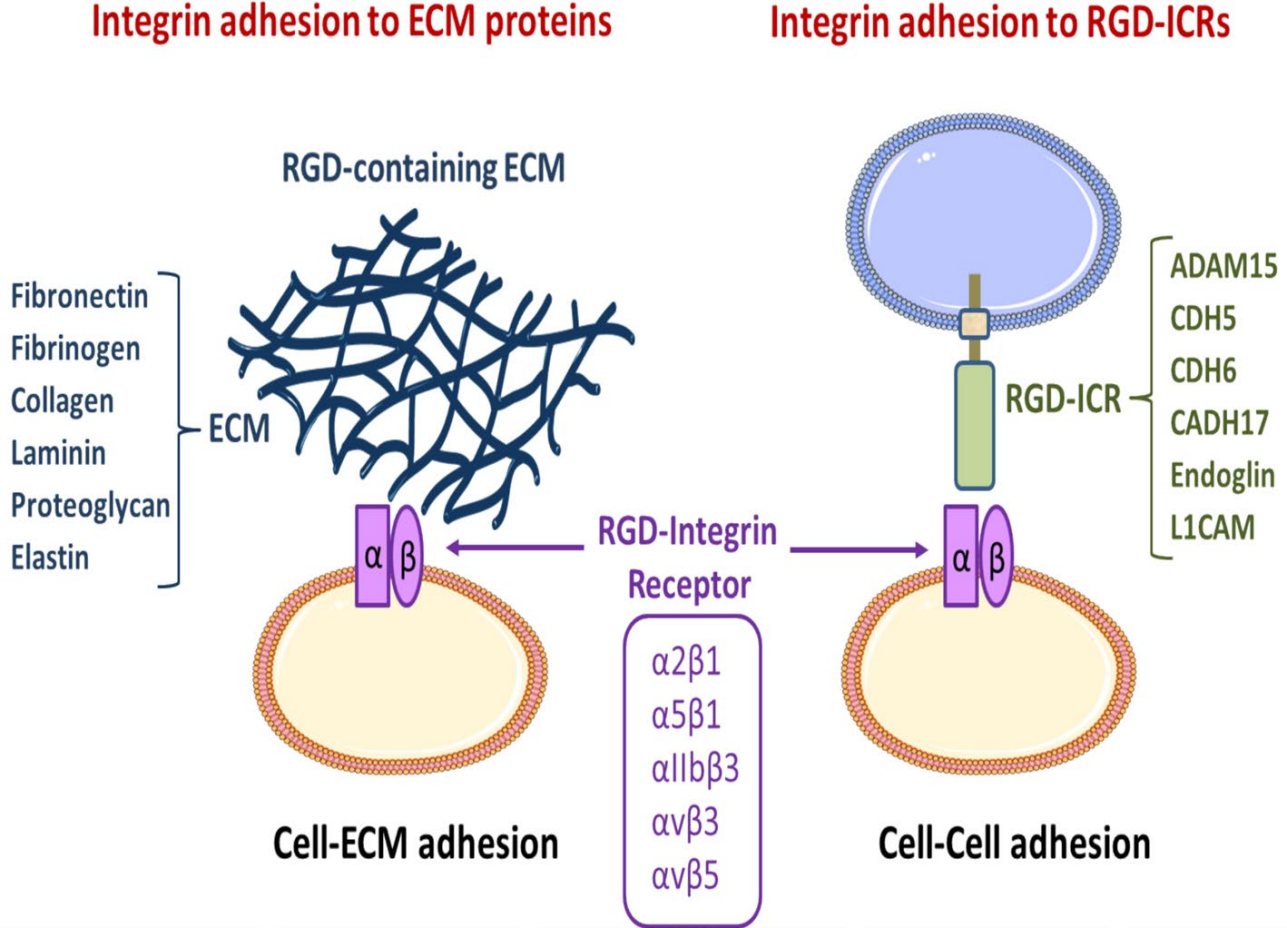
Cell adhesion mediated by RGD integrin receptors. Left, integrins interact with canonical RGD-containing proteins of the extracellular matrix (ECM) to regulate cell adhesion to ECM. Representative components of ECM (fibronectin, fibrinogen, collagen, laminin, proteoglycan, and elastin) are indicated. Right, integrins interact with the noncanonical RGD-containing integrin counter-receptors (RGD-ICRs) to regulate cell–cell adhesion. The RGD integrin receptors (α2β1, α5β1, αIIbβ3, αvβ3, and avβ5 integrins) and RGD-ICRs (ADAM15, CDH5, CDH6, CDH17, Endoglin, and L1CAM) subject of this review are listed. This figure was created using Servier Medical Art (http://smart.servier.com/)

RGD-containing ECM proteins like fibronectin are widely regarded as the canonical ligands of the subset of RGD integrin receptors, which mediate cell–ECM interactions. Conversely, the RGD-ICRs act as cell surface counter-receptors of RGD integrins mediating cell–cell interactions, thus further enriching the complex key role of the integrin family in cell adhesion (Fig. 8).

**Fig. 9:**
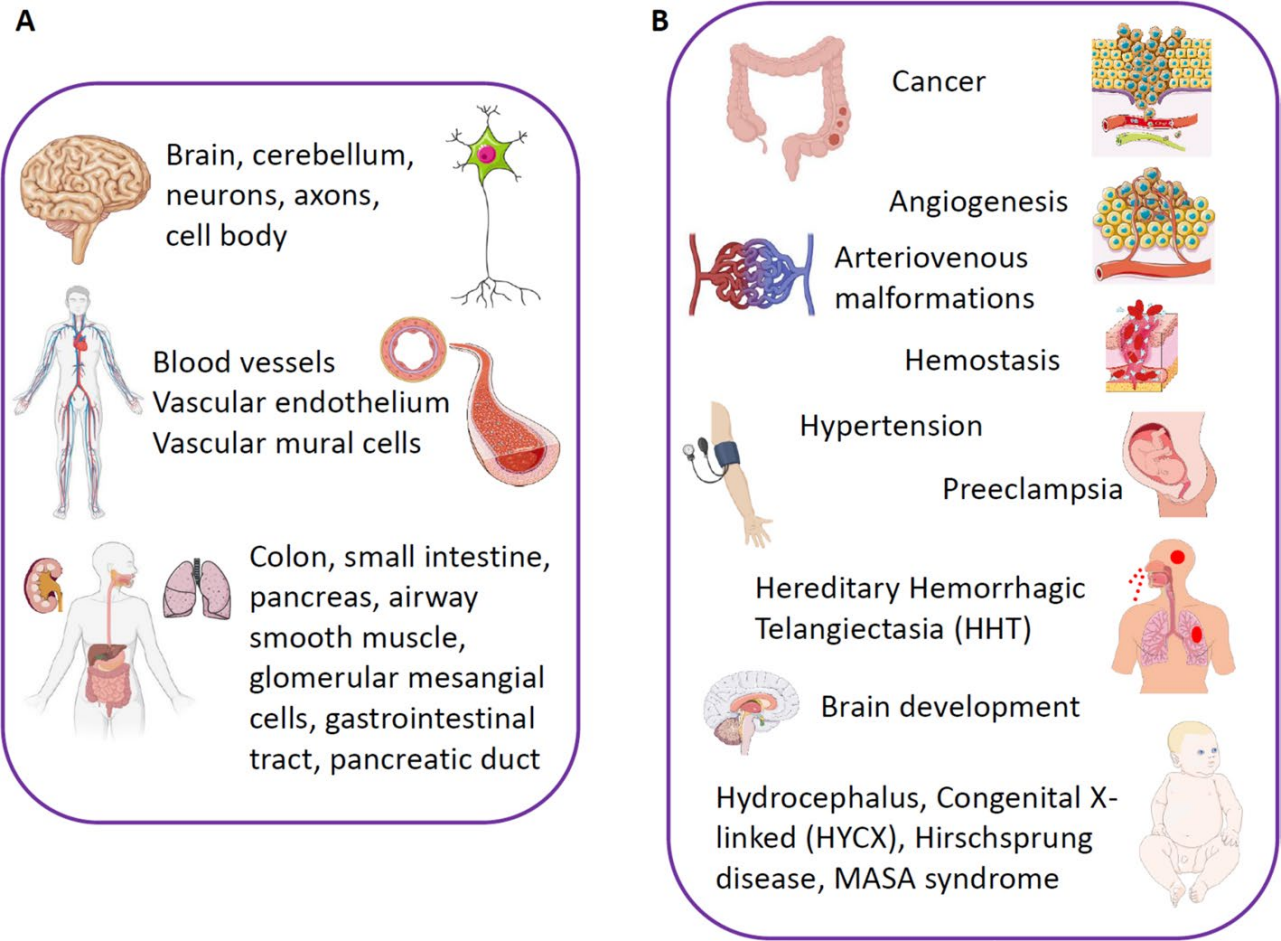
Tissue expression and pathophysiological involvement of RGD-containing Integrin counter-receptors (RGD-ICRs). **A**. Representative tissues and related cells where RGD-ICRs are expressed. **B**. Representative physiological processes and diseases in which RGD-ICRs are involved. This figure was created using Servier Medical Art (http://smart.servier.com/) and BioRender (https://www.biorender.com)

All of the RGD-ICRs subject of this study are single-pass type I transmembrane proteins with a large N-terminal extracellular region encompassing the RGD motif, compared with a much shorter cytoplasmic tail (Table 1). We have analyzed whether the six different RGD-ICRs possess any significant 3D conformational similarities among them in their RGD-containing individual domains and/or with the canonical RGD-containing integrin ligand fibronectin (FN), encompassing the type III-10 domain, which could indicate some degree of selection pressure and/or evolutionary convergence imposed upon binding of the respective integrins, leading to the acquisition of similar functional capacities. These 3D folding analyses have been performed using the AlphaFold3 and the Matchmaker function of Chimera X (version 1.8) softwares and the superimposed structures show structural similarities of the RGD-containing domains between the following pairs: (i) L1CAM and the type III-10 domain of FN; (ii) D3-CDH5 and L1CAM; (iii) D2-CDH5 and FN; (iv) CDH17 and L1CAM; and (v) CDH17 and D3-CDH5 (Supplementary Fig. 1). We have also carried out a pairwise comparison with the amino acid sequences (i.e., the primary structures) surrounding the RGD motifs (approximately 100 residues) of the respective domains in these counter-receptors, which confirmed the similarities observed in the 3D structures at the primary structure level (Supplementary Fig. 3). In contrast, no significant similarities with any other RGD-containing domains in integrin counter-receptors were observed for endoglin or ADAM15. Therefore, these analyses reveal that integrins can recognize and bind counter-receptors through interactions with RGD sites present on domains with significant homology but also on domains with very different primary and 3D structures. Each gene encoding the selected transmembrane RGD-containing proteins (endoglin, cadherin-5, −6, and −17, ADAM15, and L1CAM) maps to a different human chromosome (Table 2). In addition, each of these genes appear to show a distinct tissue and cell expression profile at transcript and protein levels (https://www.proteinatlas.org/ and https://www.uniprot.org/). As illustrated in Fig. 9A, RGD-ICRs are expressed by wide range of tissue and cell types; for example, L1CAM and CADH6 are predominantly expressed in the brain (neurons), CADH5 in the vascular endothelium and brain, and endoglin in blood vessels (vascular endothelium), whereas CDH17 is detected in the gastrointestinal tract. The heterogenous expression of RGD-ICRs is accordingly associated with a different involvement in physiological processes and diseases (Fig. 9B), as described in detail in the main text of the review. Thus, L1CAM is associated with hydrocephalus, congenital X-linked (HYCX), Hirschsprung disease, and MASA (Mental retardation, Aphasia, Shuffling gait and Adducted thumbs) syndrome; endoglin is involved in hereditary hemorrhagic telangiectasia and preeclampsia; and CADH5, CADH6, and CADH17 are linked to cancer progression and metastasis, just to name a few. Thus, the above data suggest that in spite of being different and independent, *ADAM15*, *CDH5*, *CDH6*, *CDH17*, *ENG*, and *L1CAM* genes have undergone a strong evolutionary convergence to acquire the same functional capacity to bind integrins via the RGD motif.

**Table 1:** Protein characteristics of human RGD-ICRs*

Protein (*Homo sapiens*)	UniProt no.	Cluster of differentiation	Transmembrane protein category	Total amino acids	Extracellular amino acids	Transmembrane amino acids	Cytoplasmic amino acids
ADAM15	Q13444		Single pass type I	485	489 (207–696)	20 (697–717)	145 (718–863)
CDH5 (Cadherin 5)	P33151	CD144	Single pass type I	784	551 (48–599)	20 (600–620)	163 (621–784)
CDH6 (Cadherin 6)	P55285		Single pass type I	790	561 (54–615)	20 (616–636)	153 (637–790)
CDH17 (Cadherin 17)	Q12864		Single pass type I	832	764 (23–787)	20 (788–808)	23 (809–832)
Endoglin	P17813	CD105	Single pass type I	658	560 (26–586)	24 (587–611)	46 (612–658)
L1CAM (L1-cell adhesion molecule)	P32004	CD171	Single pass type I	1257	1100 (20–1120)	22 (1121–1143)	113 (1144–1257)

^*^The human RGD-containing integrin counter-receptors (RGD-ICRs) subject of this study, their UniProt reference number (https://www.uniprot.org/), their cluster of differentiation designation (CD) when available, their transmembrane protein category, as well as the number of amino acids are indicated. Numbers in parenthesis (three columns on the right) correspond to the amino acid position within the entire protein sequence

**Table 2:** Chromosomal localization of human genes encoding RGD-ICRs*

Gene (*Homo sapiens*)	Description	Chromosome	NCBI reference
*ADAM15*	ADAM metallopeptidase domain 15	Chromosome 1	NC_000001.11
*CDH5*	Cadherin 5	Chromosome 16	NC_000016.10
*CDH6*	Cadherin 6	Chromosome 5	NC_000005.10
*CDH17*	Cadherin 17	Chromosome 8	NC_000008.11
*ENG*	Endoglin	Chromosome 9	NC_000009.12
*L1CAM*	L1-cell adhesion molecule	Chromosome X	NC_000023.11

^*^The human genes encoding the RGD-containing integrin counter-receptors (RGD-ICRs) subject of this study, their description, chromosomal location, as well as the corresponding National Center for Biotechnology Information (NCBI) reference sequence are indicated

Despite the observed structural differences among the RGD-containing domains, several specific integrins seem to be engaged by different counter-receptors. This is the case of integrin αIIbβ3, whose activation has important implications in platelet function, impinging in crucial physiological and pathological processes. αIIbβ3 is a crucial platelet receptor that is normally in a resting/inactive state in unstimulated platelets. However, signal transduction in platelets (which can be triggered through a number of different platelet surface receptors) induces rapid conformational changes and the activation of integrin αIIbβ3 through the “inside-out” activation mechanism [236]. Once activated, αIIbβ3 binds the RGD sequence on its canonical ligand fibrinogen, thus mediating platelet aggregation. In fact, a large number of antagonists of integrin αIIbβ3, including cyclic peptides containing the RGD sequence (e.g., eptifibatide), small inhibitor drugs (e.g., tirofiban), and blocking monoclonal antibodies (e.g., abciximab) are clinically approved to treat acute coronary syndromes (including unstable angina and non-ST elevation myocardial infarction) that need percutaneous coronary intervention (PCI) [236, 237]. In this regard, endoglin on endothelial cells favors αIIbβ3 integrin-mediated adhesion of platelets to the endothelium and plays a role in hemostasis and thromboinflammatory events [36, 83]. Furthermore, the circulating form of human endoglin (sEng) (which can be released by several proteases) has also been shown to bind αIIbβ3 integrin, which may constitute an additional layer of regulation in the platelet–endothelium adhesion process [37]. Moreover, RGD-containing cadherins seem to play a key role in the activation of αIIbβ3 integrin and in platelet function. CDH6, whose surface expression increases in thrombin-activated platelets, is an RGD ligand for integrin αIIbβ3 and facilitates platelet aggregation [108]. Of note, integrin α2β1 supports integrin αIIbβ3 activation and platelet adhesion to collagen [238] in a way that reminds the crosstalk between both integrins observed in cancer cells [118]. Likewise, CDH17 can also bind αIIbβ3 integrin, and the potential binding of CDH5 to αIIbβ3 integrin is also plausible, though remains unexplored. In this regard, CADH5 and endoglin are both expressed in endothelial cells and physically and functionally associate with each other [239], suggesting their joint involvement in αIIbβ3 integrin-mediated thromboembolic events [37]. The RGD sequence in the disintegrin domain of ADAM15 on endothelial cells can also be recognized by platelet αIIbβ3 and these ADAM15–αIIbβ3 interactions have been shown to mediate the adhesion of platelets to endothelium [188]. Furthermore, it has been shown that the binding of platelet αIIbβ3 integrin to endothelial ADAM15 results in the activation and recruitment of additional platelets, which leads to thrombus formation, underscoring the role of ADAM15 in atherosclerosis [166]. Finally, the RGD-site(s) in the sixth Ig-domain of L1CAM have been shown to support the binding to platelet αIIbβ3 integrin [201], although the physiopathological implications of these interactions have not been explored. Therefore, an emerging concept is that all the RGD-containing counter-receptors considered in this review seem to have the capacity to bind the αIIbβ3 integrin, with the potential to regulate platelet adhesion, aggregation, and function, including their involvement in hemostasis, atherosclerosis, and thrombotic pathogenesis. This capacity indicates a complex regulation of αIIbβ3 that deserves further research to explore how these counter-receptor compete or collaborate in these functions. Another potential application is the repurposing of thrombolytic drugs for application in unrelated diseases (i.e., cancer). Moreover, specific anti-RGD monoclonal antibodies have shown efficacy in the blocking of RGD cadherins binding to either α2β1 [103] or αIIbβ3 integrins [118], paving the way to their potential use in the blocking of other RGD counter-receptors. In this regard, the significant homology between CDH17 and L1CAM RGD motifs would suggest a simultaneous blocking of both activities by the RGD-specific monoclonal antibodies in colorectal cancer. This potential capacity warrants further investigations.

Another interesting feature shared by several members of the selected transmembrane RGD-containing proteins (endoglin, CDH17, CDH6, ADAM15, and L1CAM) is their involvement in different aspects of cancer, including tumor growth, tumor malignancy, tumor vascularization, metastasis, and cancer progression and prognosis. This finding adds novel therapeutic opportunities to the field of RGD motif in the targeting of cancer and other conditions [240, 241]. In this field, several inhibitors to RGD integrins such as those involving αv (β1, β3, β6, and β8), αIIb (β3), α5 (β1), and α8 (β1) subunits are used in preclinical and clinical studies to treat cancer, fibrosis, macular degeneration, diabetic macular oedema, and pathological hemostasis [241]. In addition, RGD peptides and RGD-functionalized drug carriers are promising options for cancer therapy [240]. Interestingly, CDH5, CDH17, and L1CAM share their involvement in several metastatic cancers [102, 103, 114, 242, 243], which could be related to the pairwise sequence alignment found near the RGD motif for the combinations CDH17/L1CAM and CDH17/CDH5 (its second RGD motif). By contrast, the neutralizing antibody TRC105 (Carotuximab^®^, Tracon Pharmaceuticals, San Diego, CA, USA) against the RGD-containing endothelial endoglin has been used as a therapeutic antiangiogenic strategy in preclinical cancer models, as well as in phase I–III clinical trials of patients with cancer [93, 97].

Interestingly, the extracellular portion (ectodomain) of the majority of the RGD-counter-receptors discussed in this review can be released from the cell surface by the action of specific proteases, a process known as ectodomain shedding. The shedding of endoglin, CDH5, CDH17, and L1CAM has been reported in literature, and the proteases responsible include thrombin, MMP14, and MMP12 for the shedding of endoglin; ADAM10 for the shedding of CDH5; and ADAM10 and ADAM17 for the shedding of L1CAM. A similar shedding has been also observed for CDH17, although in this case, the proteases involved have not been characterized yet. Therefore, these RGD-containing integrin counter-receptors can be detected at least in two forms: a cell-surface transmembrane form and a circulating soluble form. Through either additional integrin activation or competition with the cell-associated transmembrane forms, the soluble forms of these molecules can potentially add an additional layer of regulation to crucial functions mediated by integrin receptors and their RGD-containing counter-receptors, such as cell–cell and cell–ECM adhesion phenomena, migration, invasion, and proliferation. Biochemically, the protease-mediated shedding of the RGD-containing counter-receptors endoglin, CDH5, CDH17, and L1CAM and their release in soluble forms is an irreversible process and indicates that the balance between their respective transmembrane and soluble forms may play crucial roles related to their specific involvement in certain physiological and pathological conditions. Accordingly, their proteolytic processing must be subjected to exquisitely regulated mechanisms. Importantly, a correlation has been found between the soluble levels of some of these molecules and certain pathological conditions. For instance, soluble endoglin has been found to contribute to the pathogenesis of preeclampsia [88, 89, 244]. In addition, serum levels of soluble CDH5 or soluble endoglin were significantly increased in patients suffering from severe sepsis or septic shock, several with clinical signs of multiple organ dysfunction syndrome or microvascular leackage, [245–247]. Likewise, in patients with cancer: (i) serum levels of soluble L1CAM are elevated in breast cancer and associate with poor prognosis [248]; (ii) a relationship has been also found between increased serum sL1CAM level and poor clinicopathological features in type 1 endometrial cancers [249]; and (iii) increased serum or plasma levels of sEng correlate with metastasis or poor survival in certain cancers, including colorectal carcinoma, prostate cancer, and myeloid malignancies [38, 93, 97]. Therefore, the circulating soluble levels of these molecules may represent novel useful biomarkers in a number of malignant and nonmalignant pathologies, whose determination in noninvasive liquid biopsies could greatly assist in establishing an earlier diagnosis and a more precise prognosis.

In summary, RGD-containing counter-receptors have emerged as a new family of promising biomarkers and therapeutic targets, with clinical potential in a wide array of different pathologies. Cross-investigations between these molecules should definitively be carried out for further clarification.

## Supplementary Information

Additional file 1.

## Data Availability

Not applicable.

## Abbreviations

ADAM: A disintegrin and metalloproteinase
AVMs: Arteriovenous malformations
BMP: Bone morphogenetic protein
Bp: Base pair
CAFs: Cancer-associated fibroblasts
CDH: Cadherin
CEA-CAMs: Carcinoembryonic antigen-related cell adhesion molecules
ECM: Extracellular matrix
EMT: Epithelial–mesenchymal transition
Eng: Endoglin
FAK: Focal adhesion kinase
FGFR2: Fibroblast growth factor receptor 2
FN: Fibronectin
GBM: Glomerular basement membrane
HELLP: Hemolysis, elevated liver enzymes, low platelets syndrome
HHT: Hereditary hemorrhagic telangiectasia
HYCX: Hydrocephalus, congenital X-linked
IBD: Inflammatory bowel disease
IECs: Intestinal epithelial cells
LADs: Leukocyte adhesion deficiencies
MIDAS: Metal-ion-dependent adhesion site
MMPs: Matrix metalloproteinases
NSCLC: Non-small-cell lung cancer
RA: Rheumatoid arthritis
RGD-ICRs: RGD-containing integrin counter-receptors
sEng: Soluble endoglin
SPR: Surface plasmon resonance
SVDs: Snake venom disintegrins
SVMPs: Snake venom metalloproteinases
TALs: Tumor-associated lymphocytes
TAMs: Tumor-associated macrophages
TGF-β: Transforming growth factor β
VEGF: Vascular endothelial growth factor
VLA: Very late activation antigen integrin
