# Supplementary material for: The emerging role of human transmembrane RGD-based counter-receptors of integrins in health and disease
Source: Cell Mol Biol Lett. 2025 Oct 2;30:110. doi: 10.1186/s11658-025-00787-7 (PMC12492590; doi:10.1186/s11658-025-00787-7)
Supplement: Supplementary file 1 — Additional file 1. [file 11658_2025_787_MOESM1_ESM.docx]

**Supplementary Figure 1**

**
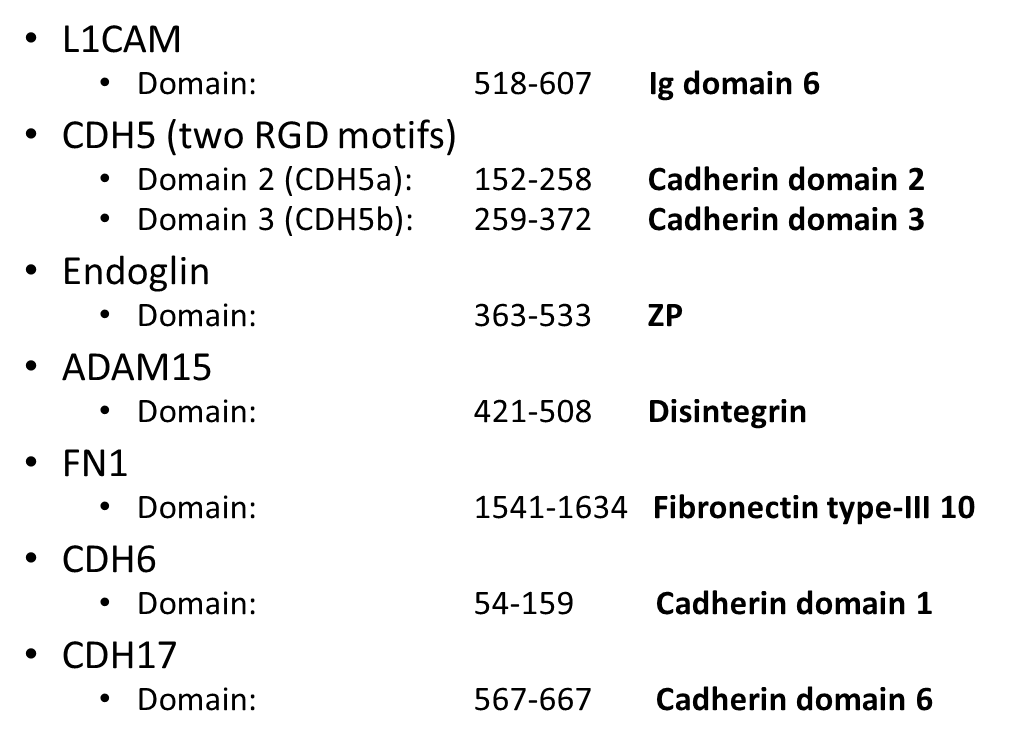
**

**Supplementary Figure 1.** **RGD-containing domains and sequences in integrin counter-receptors.** The specific domain/s within each integrin counter-receptor and in fibronectin 1 (FN1) that contain the RGD motifs are indicated. The numbers represent stretches of approximately 100 residues surrounding the RGD-motifs in each counter-receptor and in FN1, which have been further aligned in supplementary figure 3 to reveal sequence homologies in primary structures.

**Supplementary Figure 2**

**
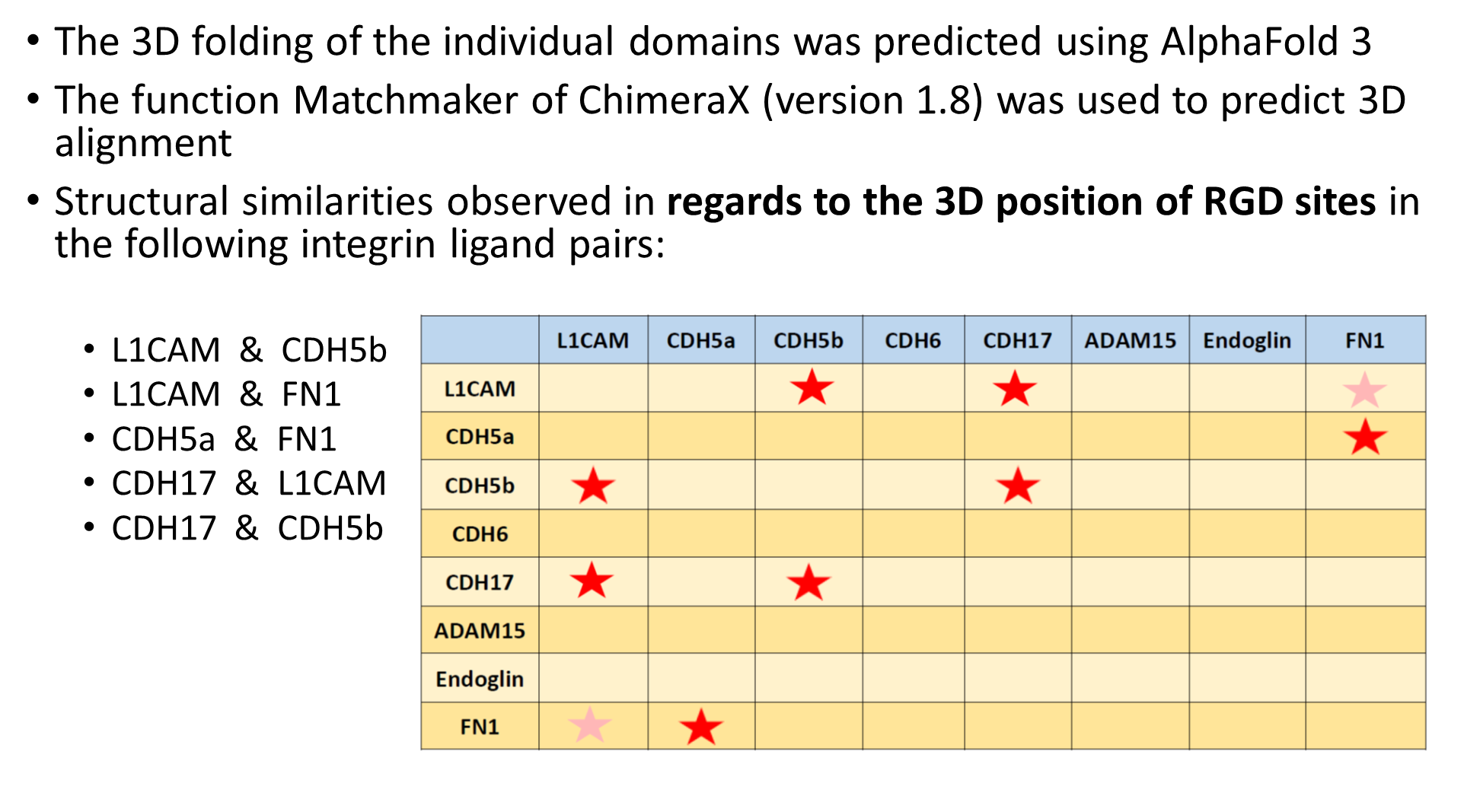
**

**Supplementary Figure 2.** **3D structural similarities among RGD-containing integrin counter-receptors.** The bioinformatic tools employed to predict and align the 3D structures of the RGD-containing integrin counter-receptors and fibronectin-1 (FN1) are indicated. The strong-red stars in the table indicate structural similarities between specific pairs of RGD-containing integrin ligands, based on the spatial position of their respective RGD motifs. The pale-red stars denote a weaker structural similarity between L1CAM and FN1, according to a less coincident position of their respective RGD motifs.

**Supplementary Figure 3**

**
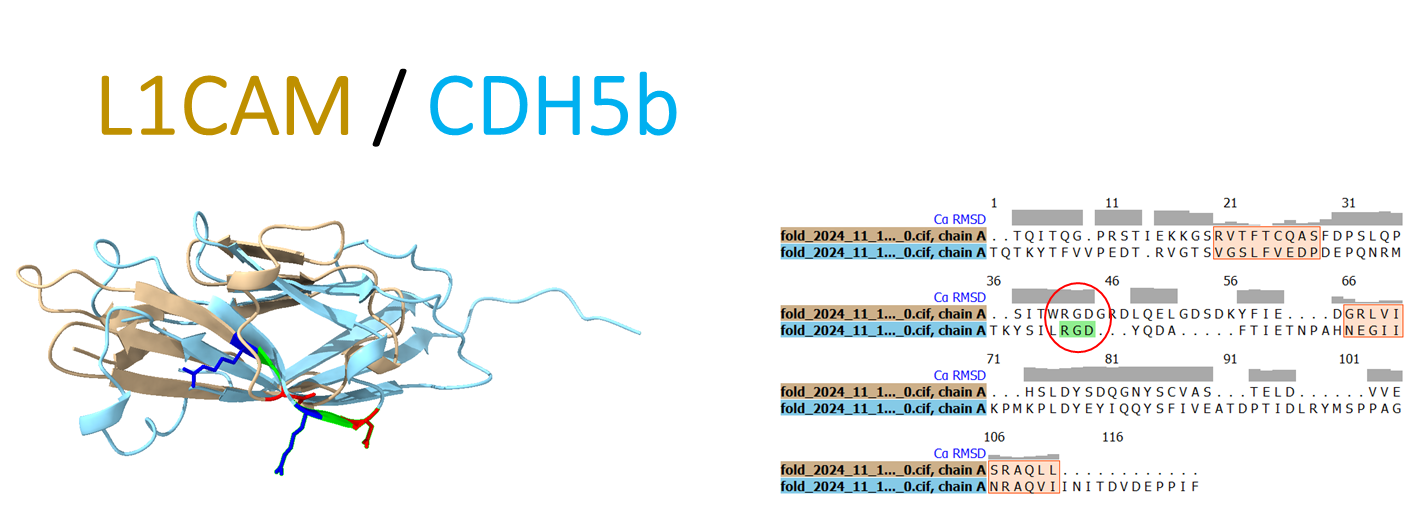

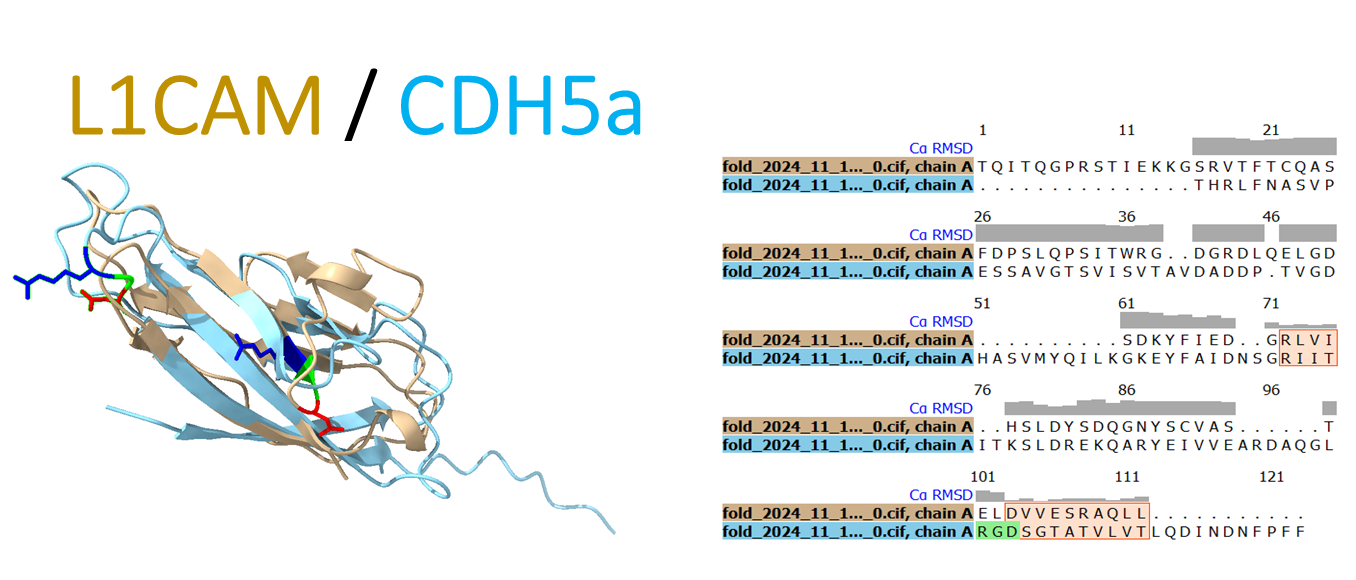

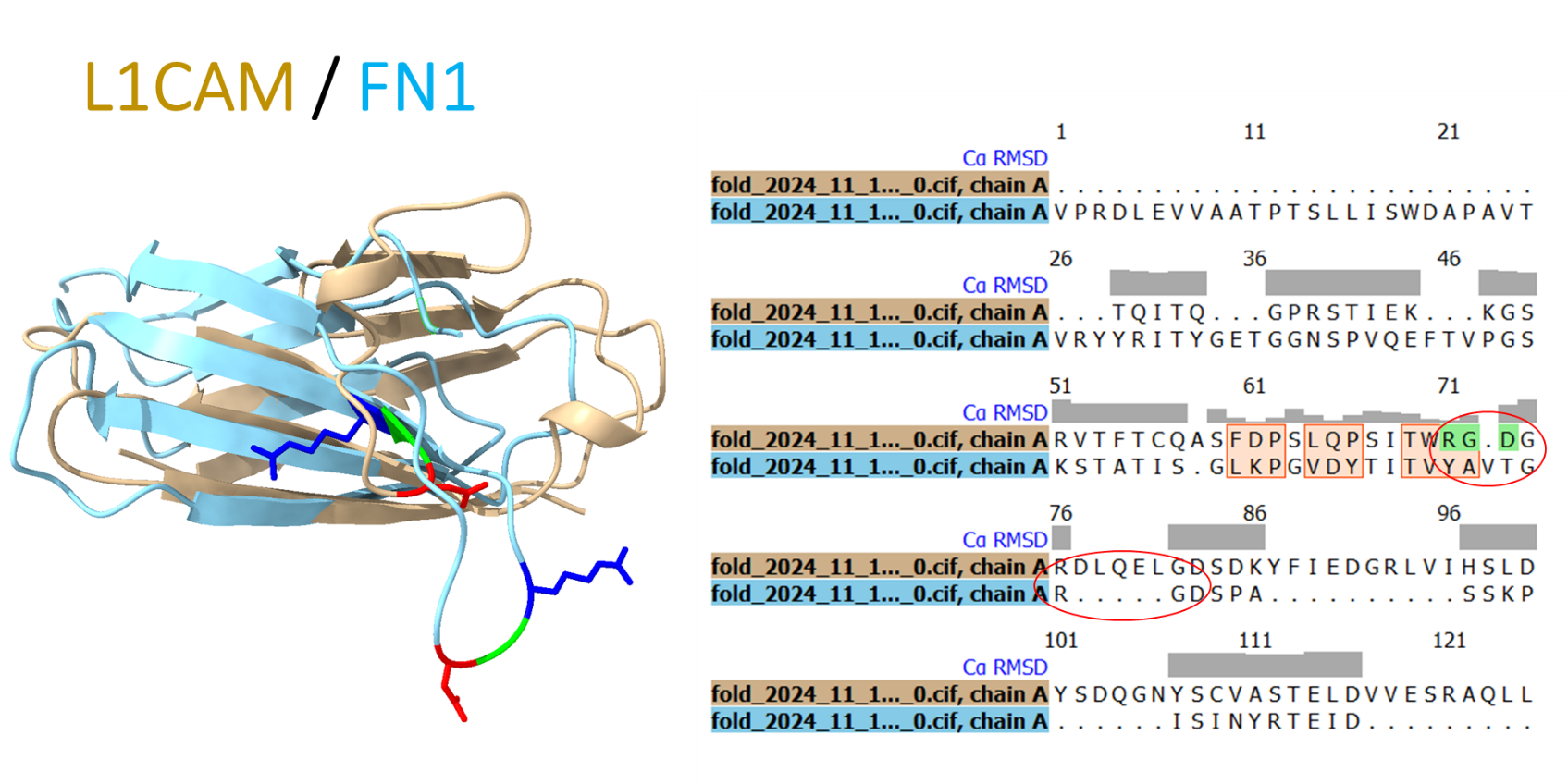
A)**


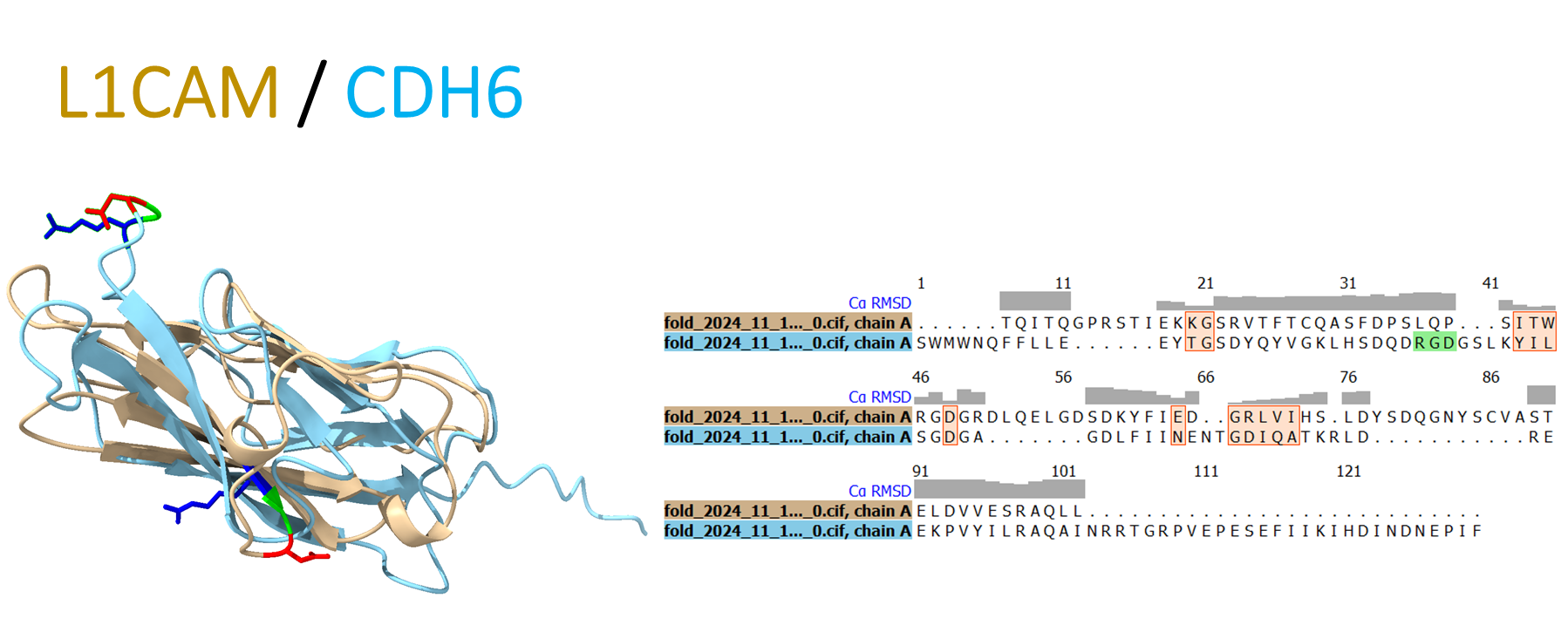

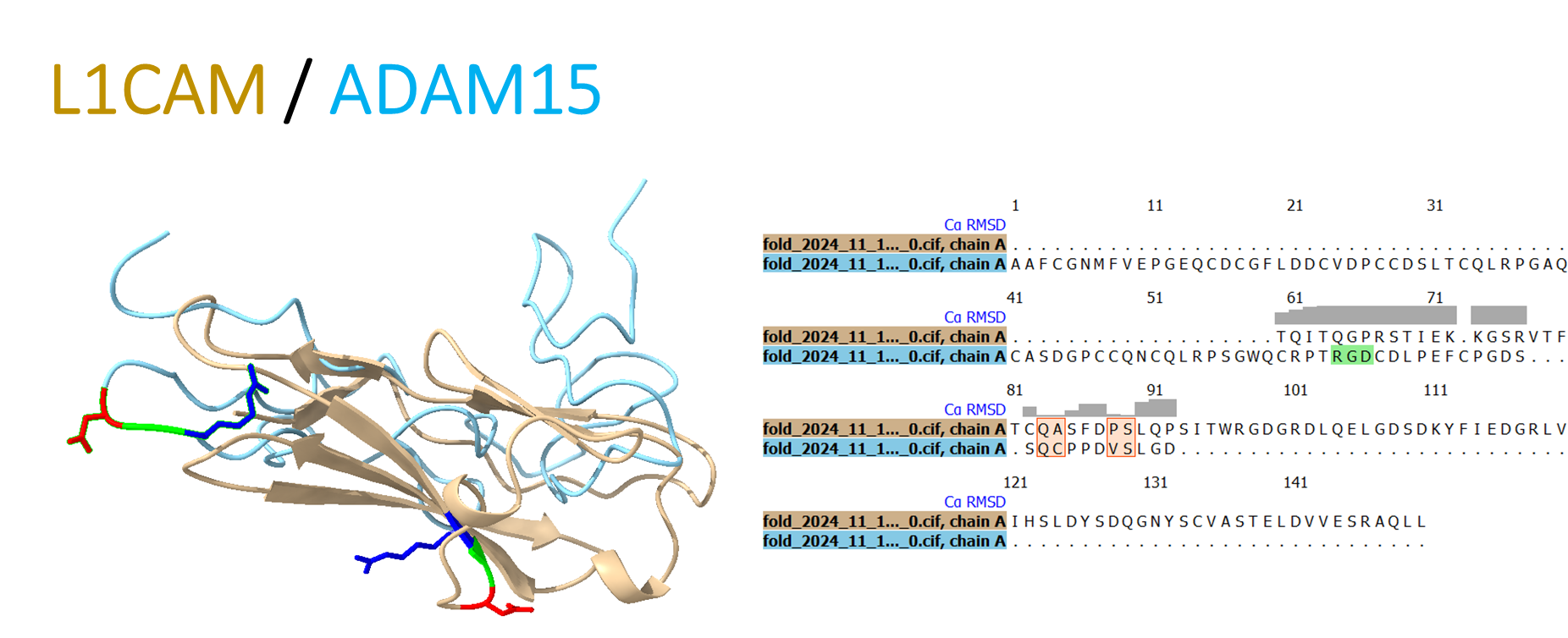

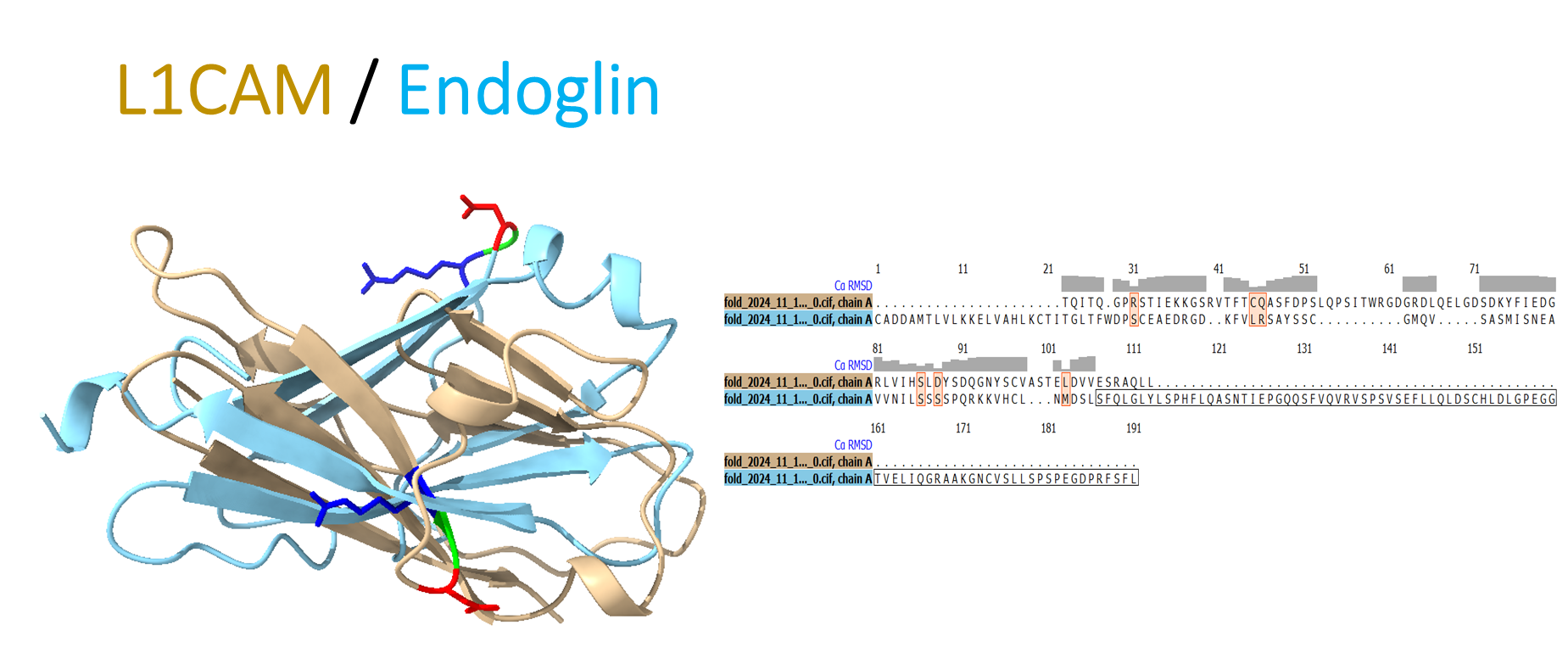


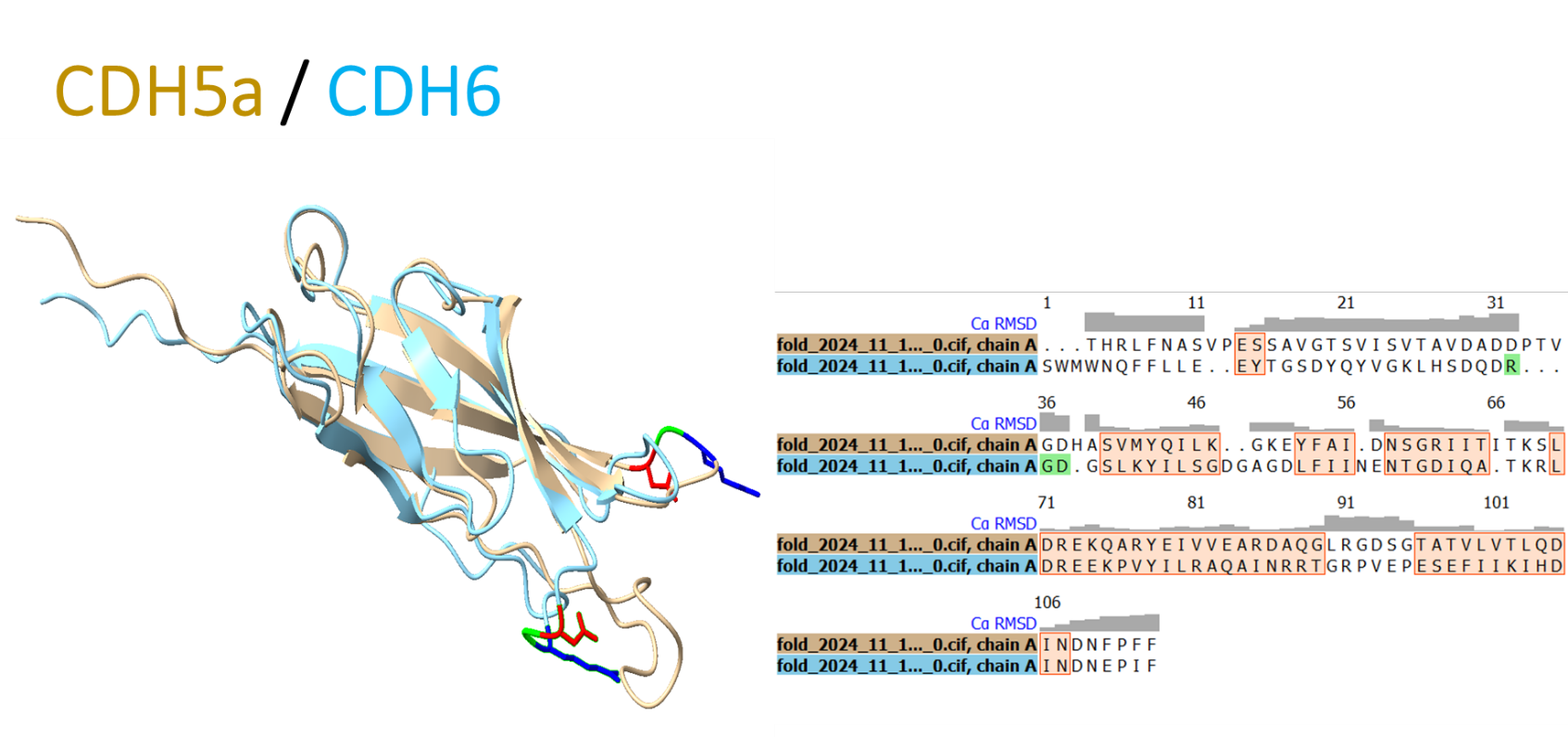

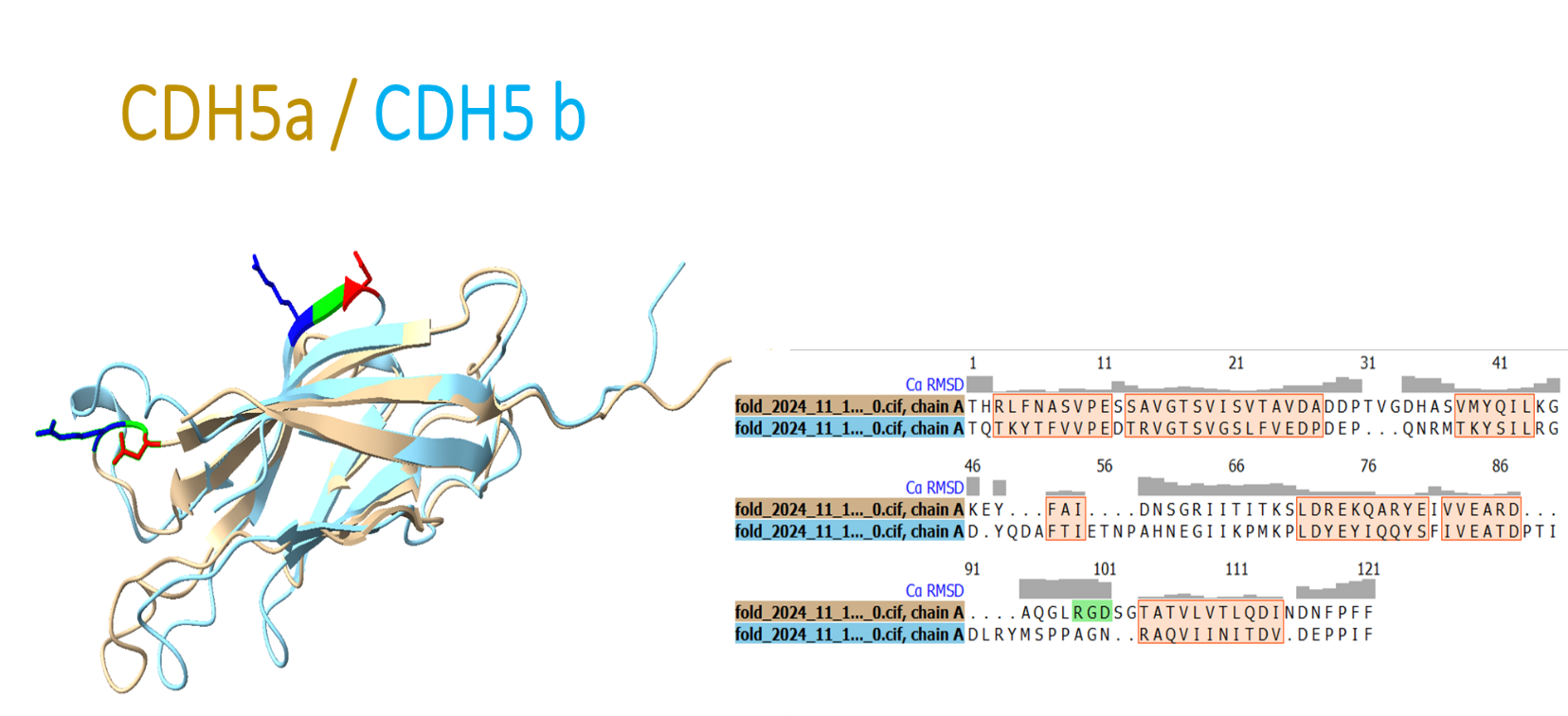

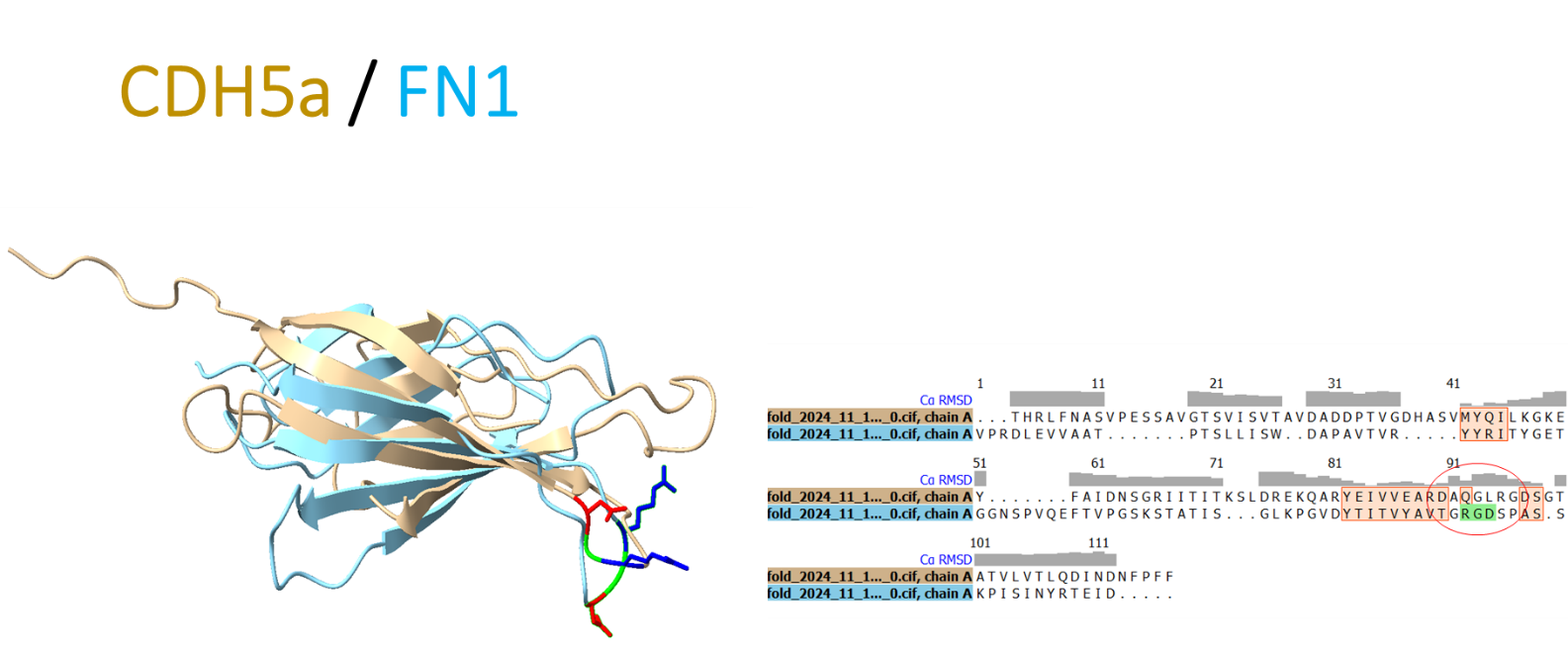
**B)**


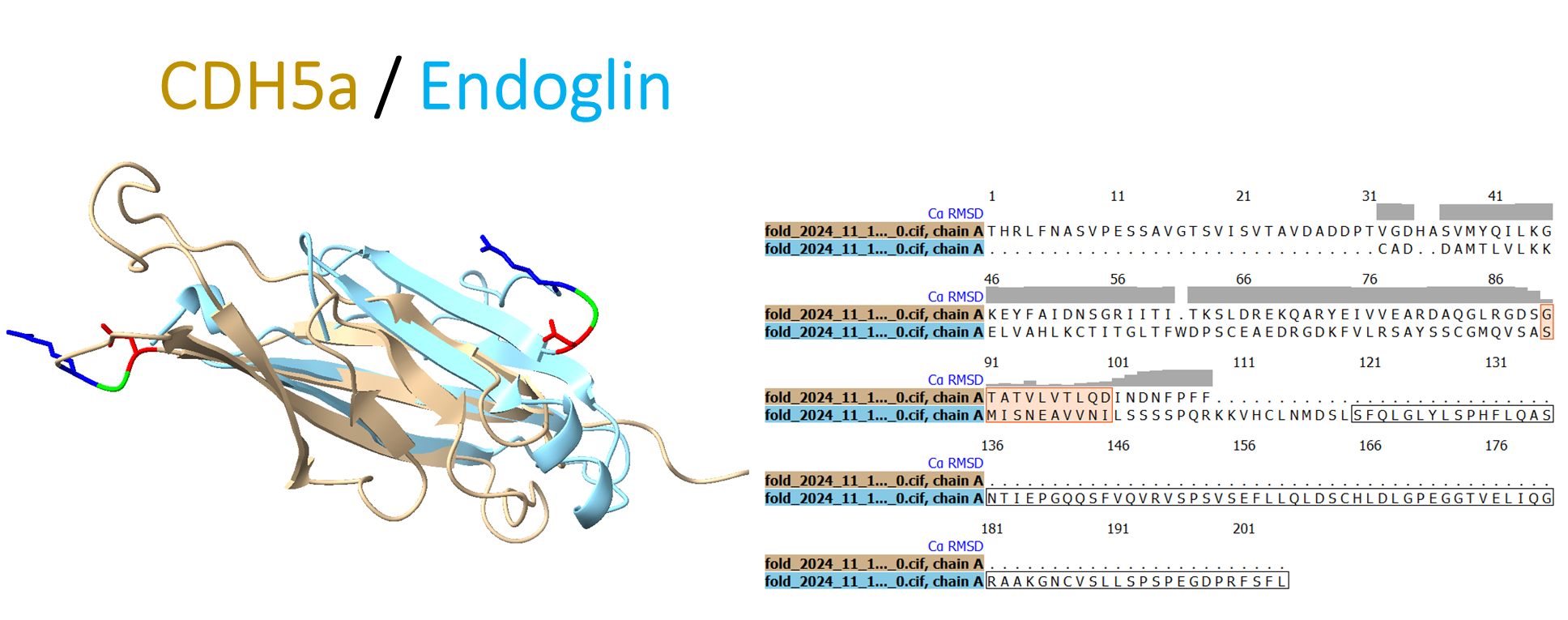

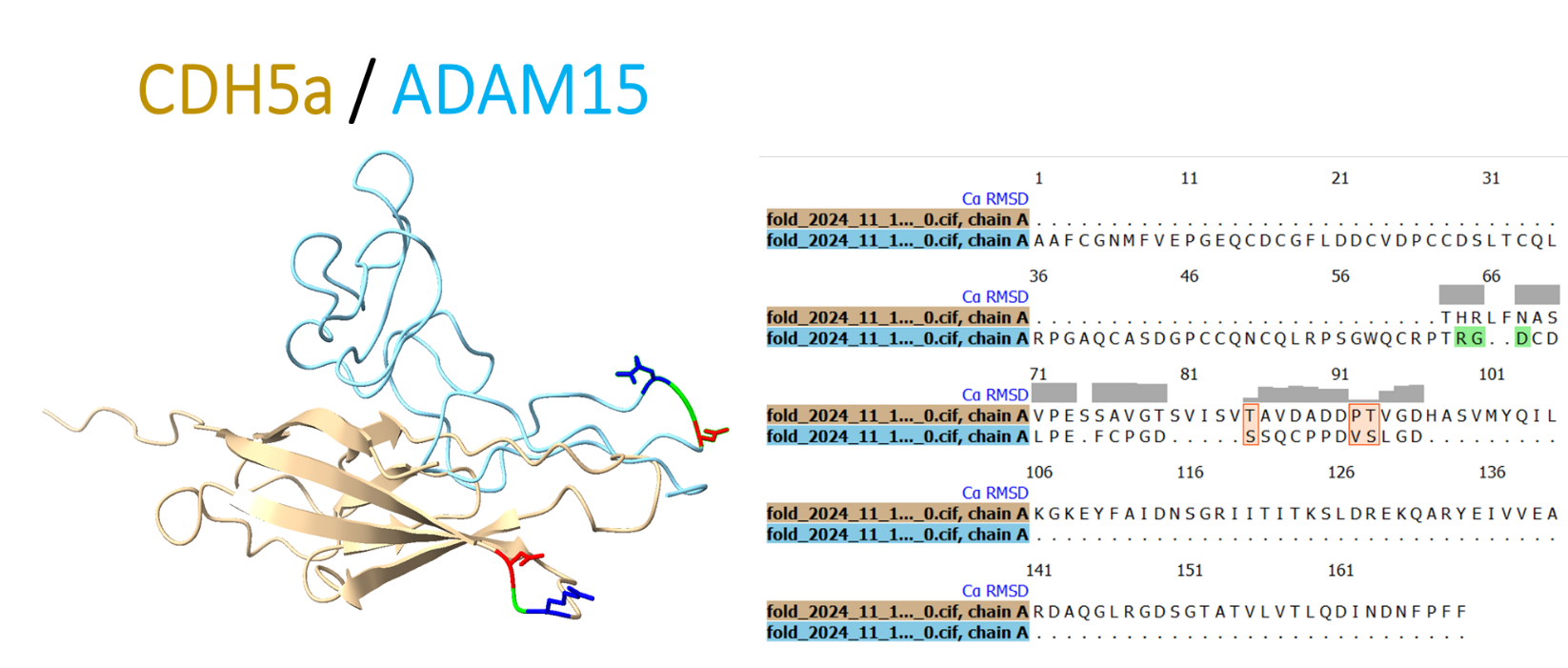


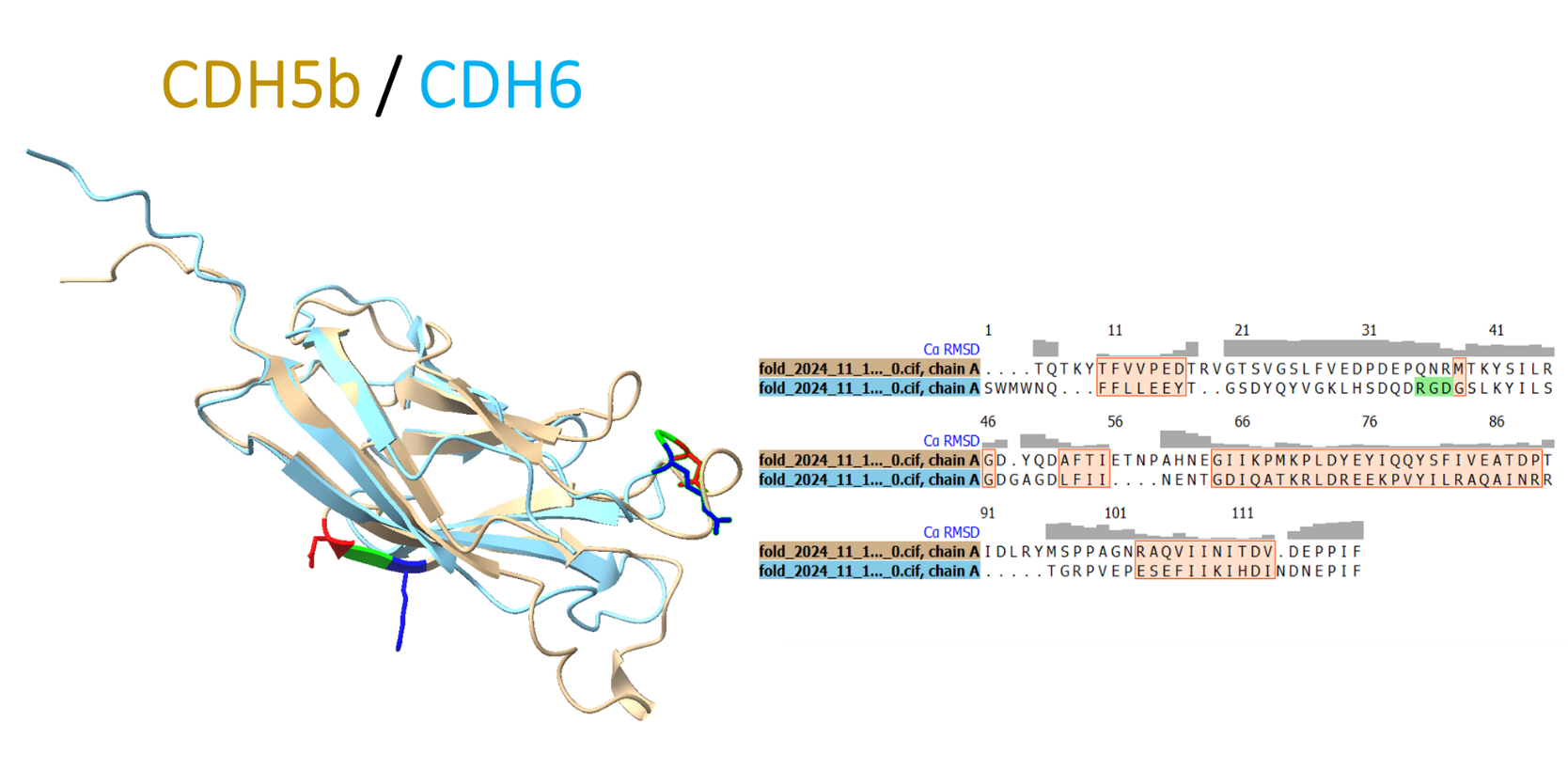

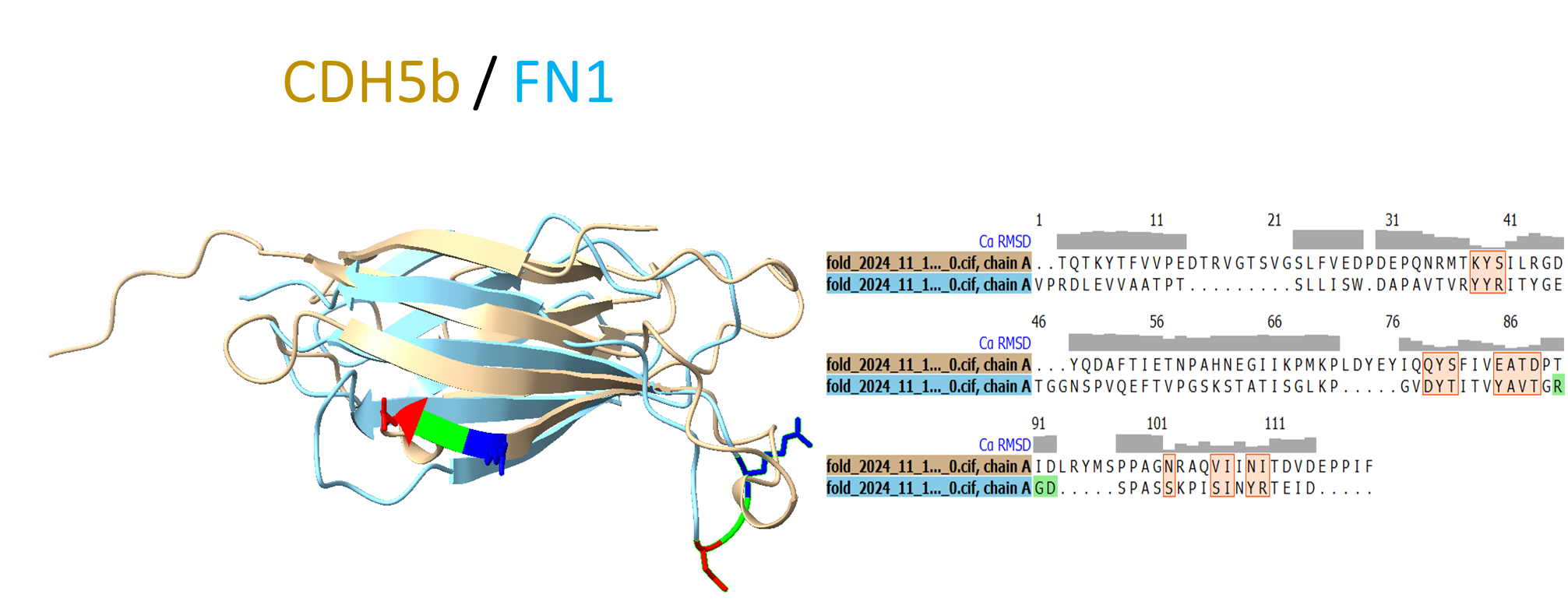
**C)**


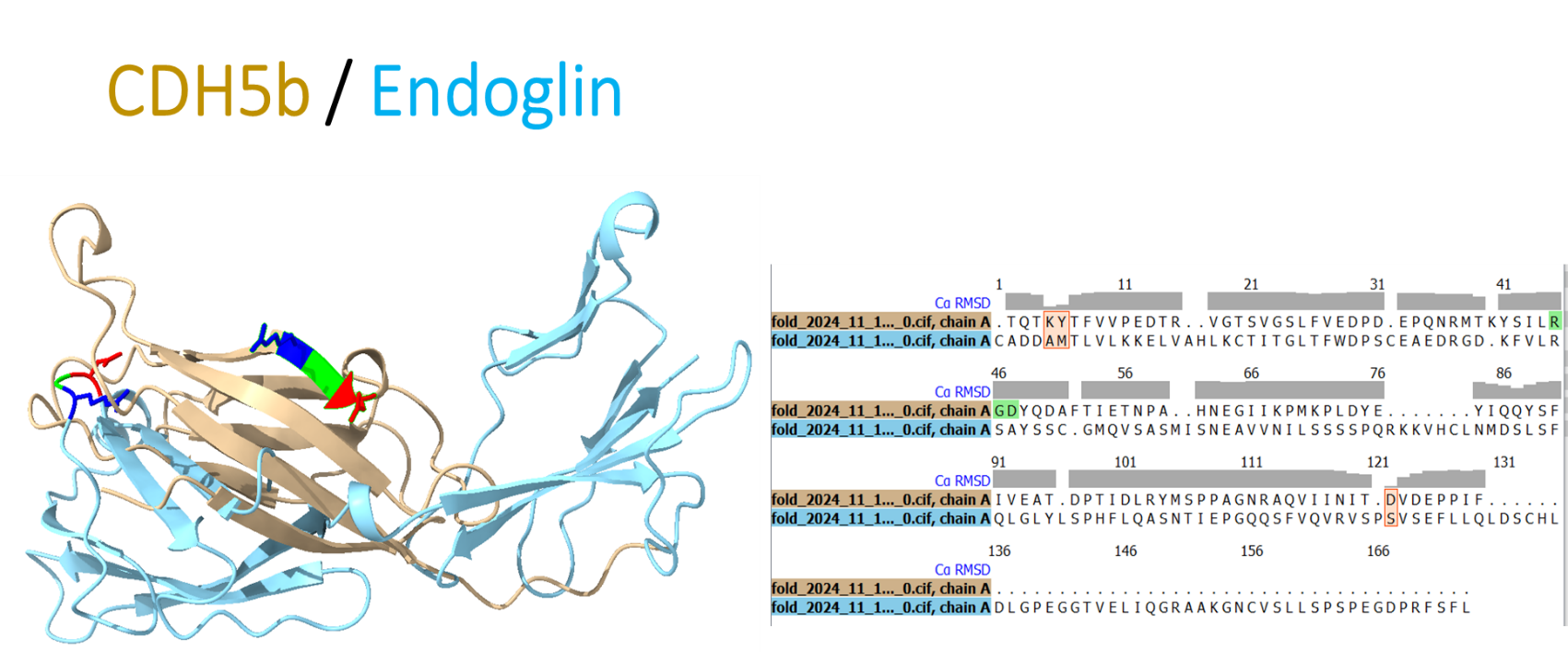


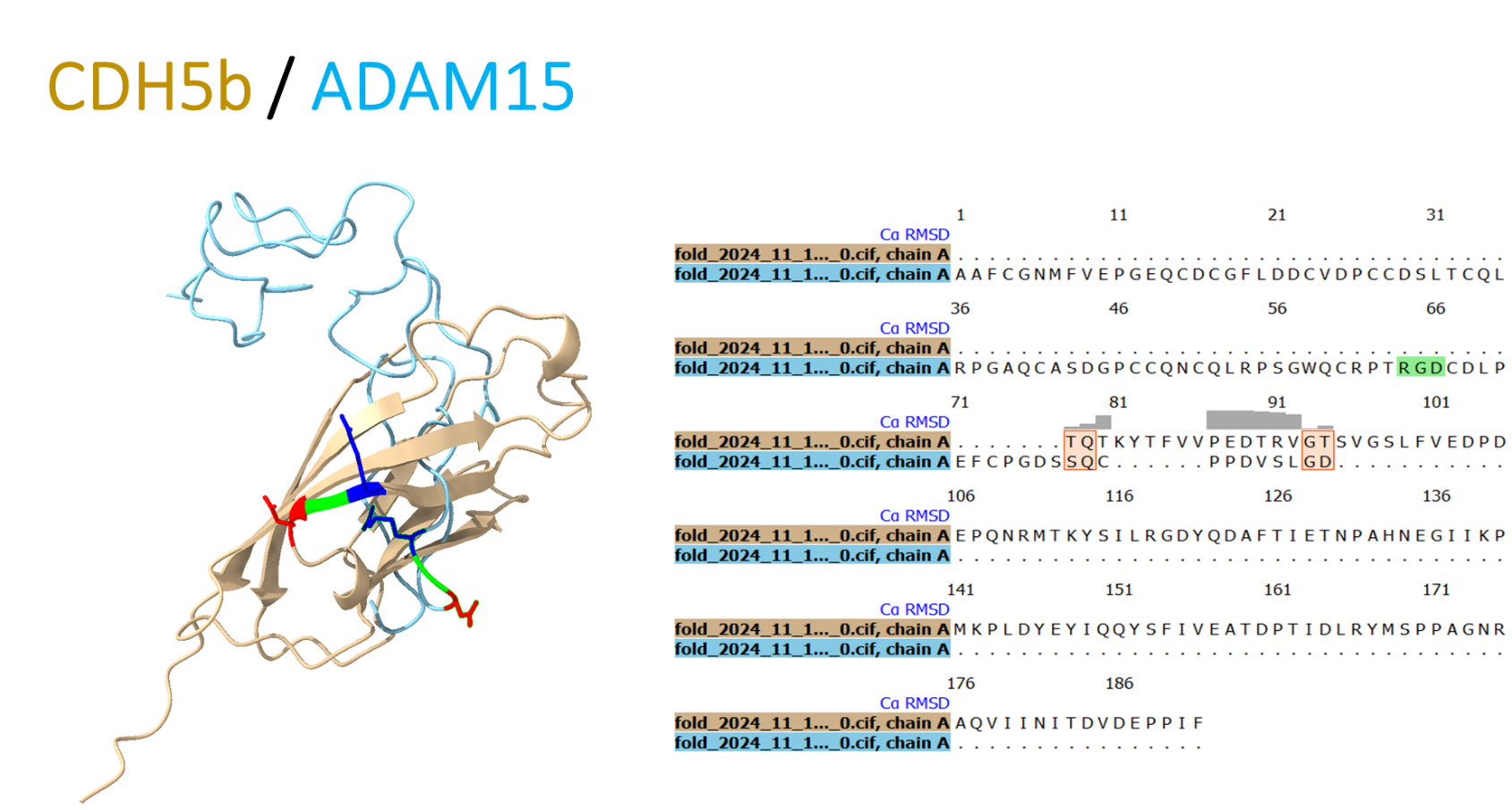


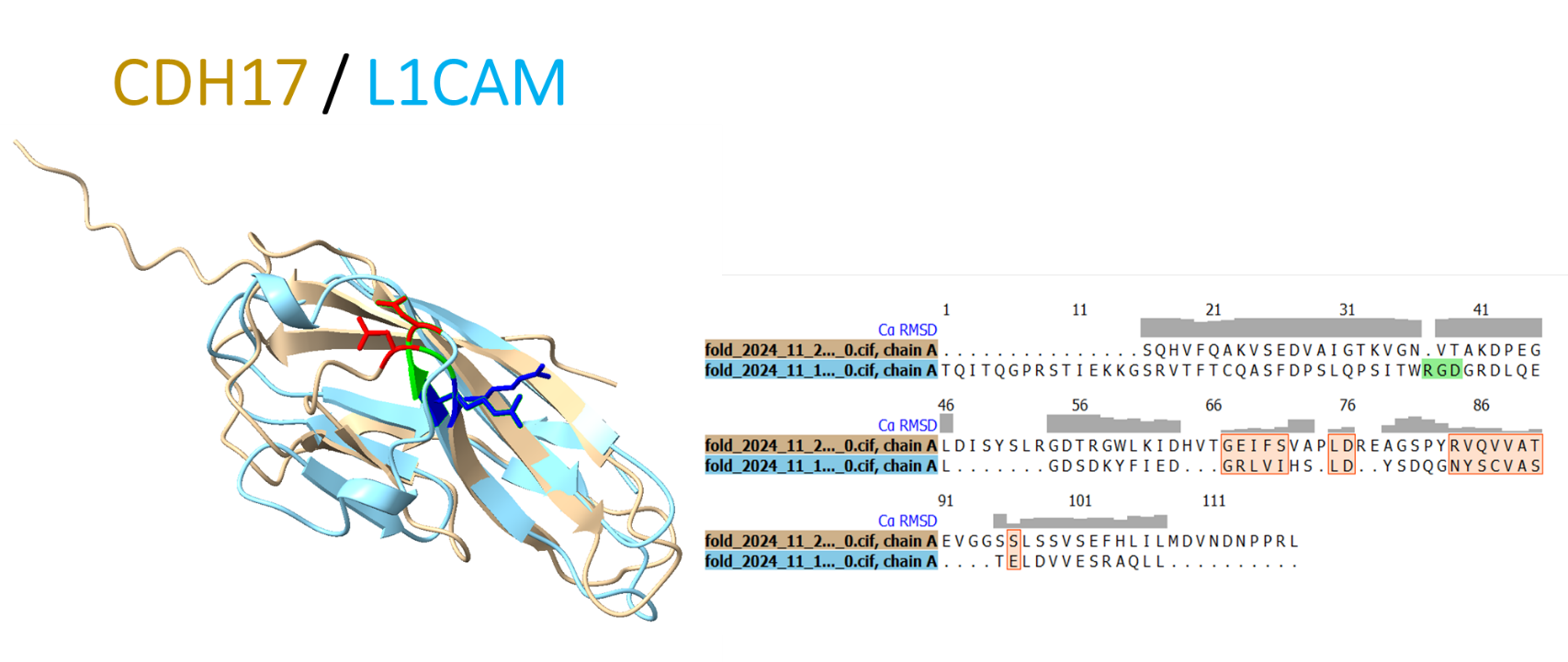

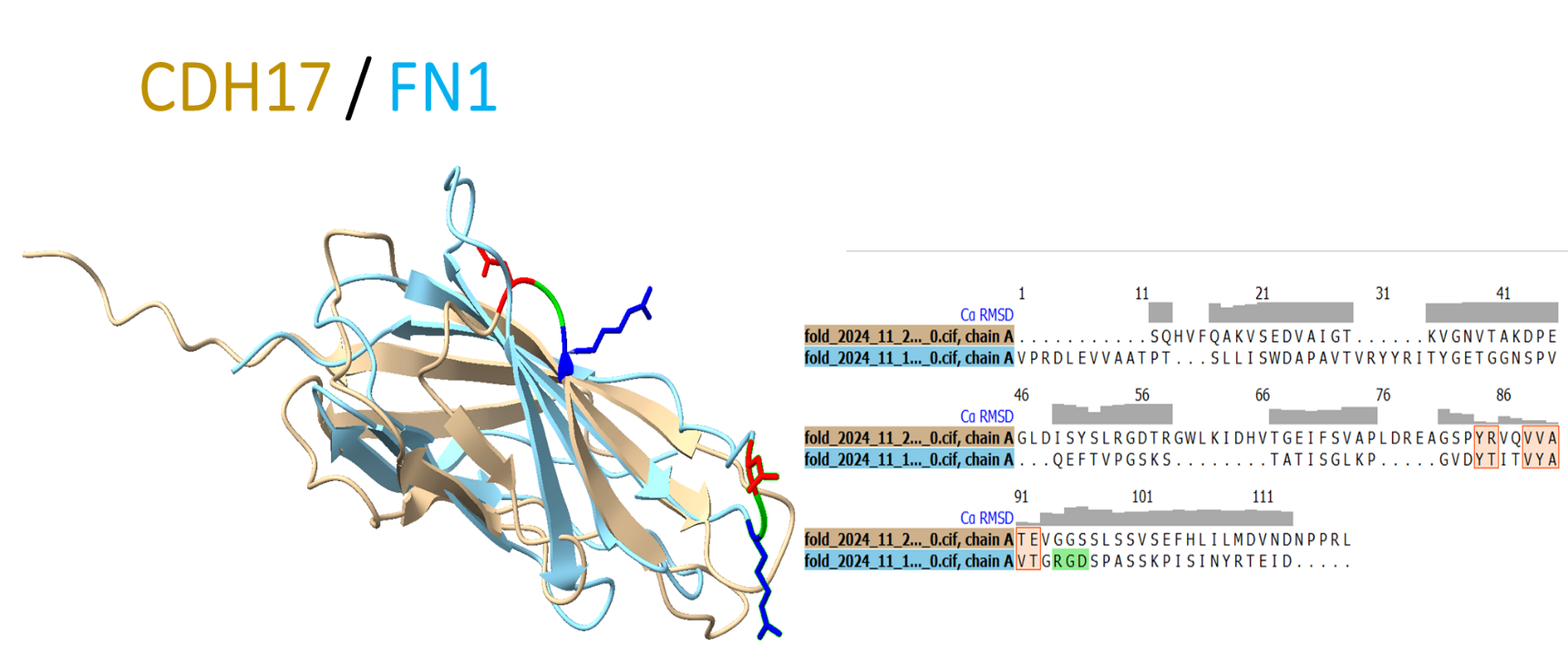
**D)**


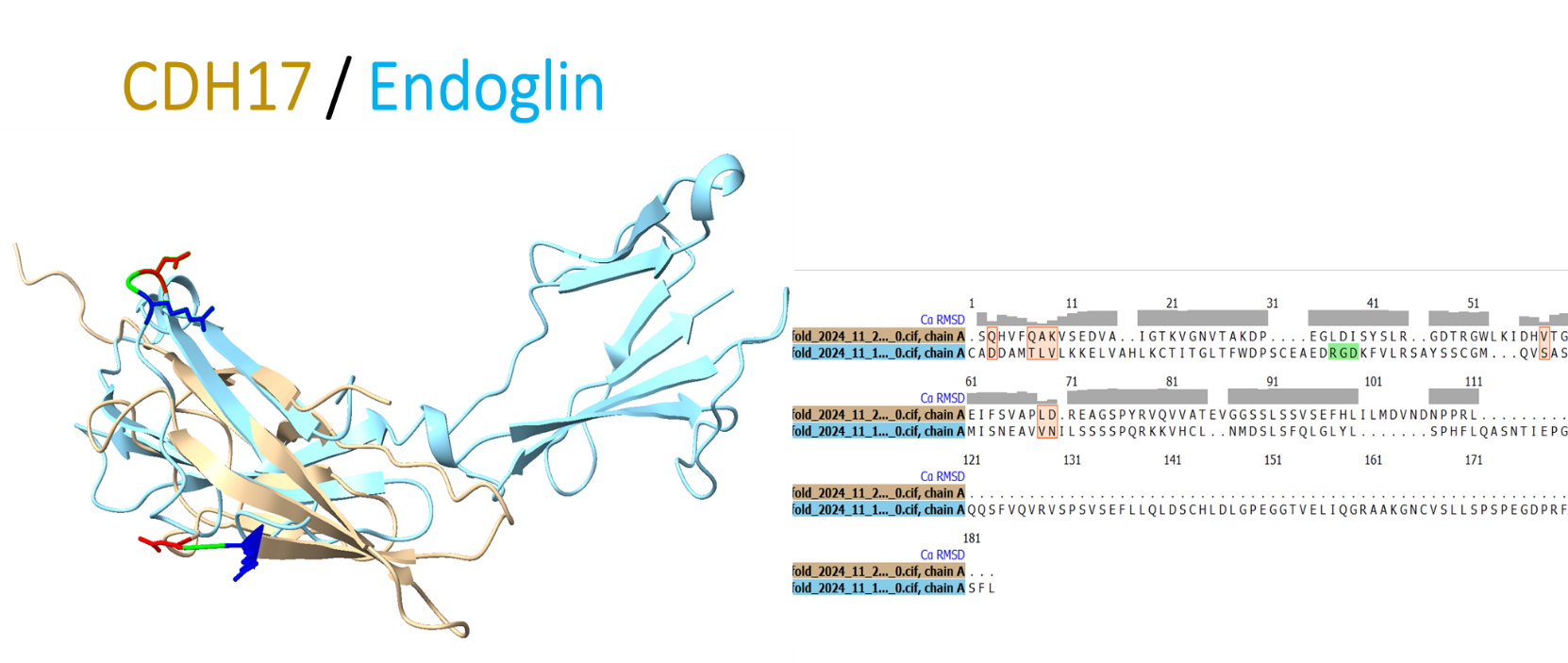


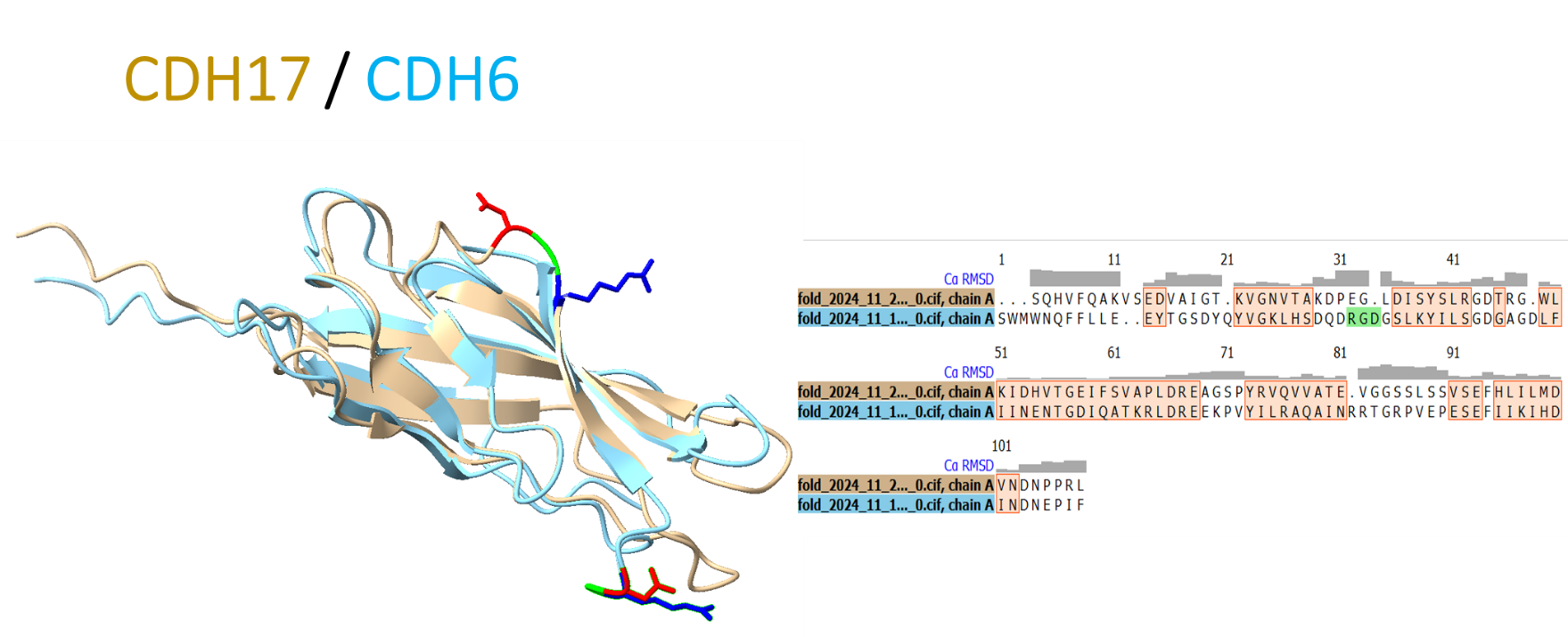

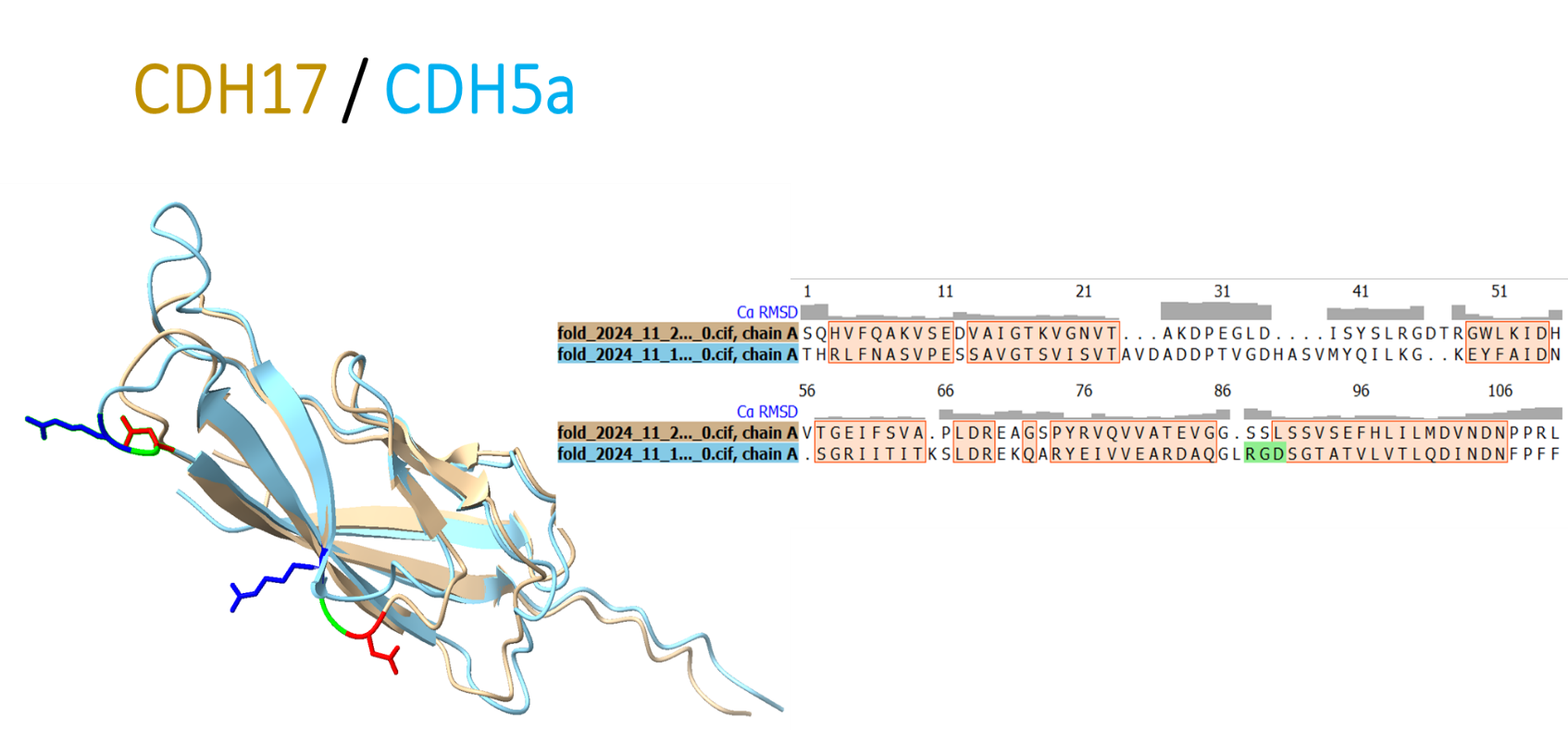

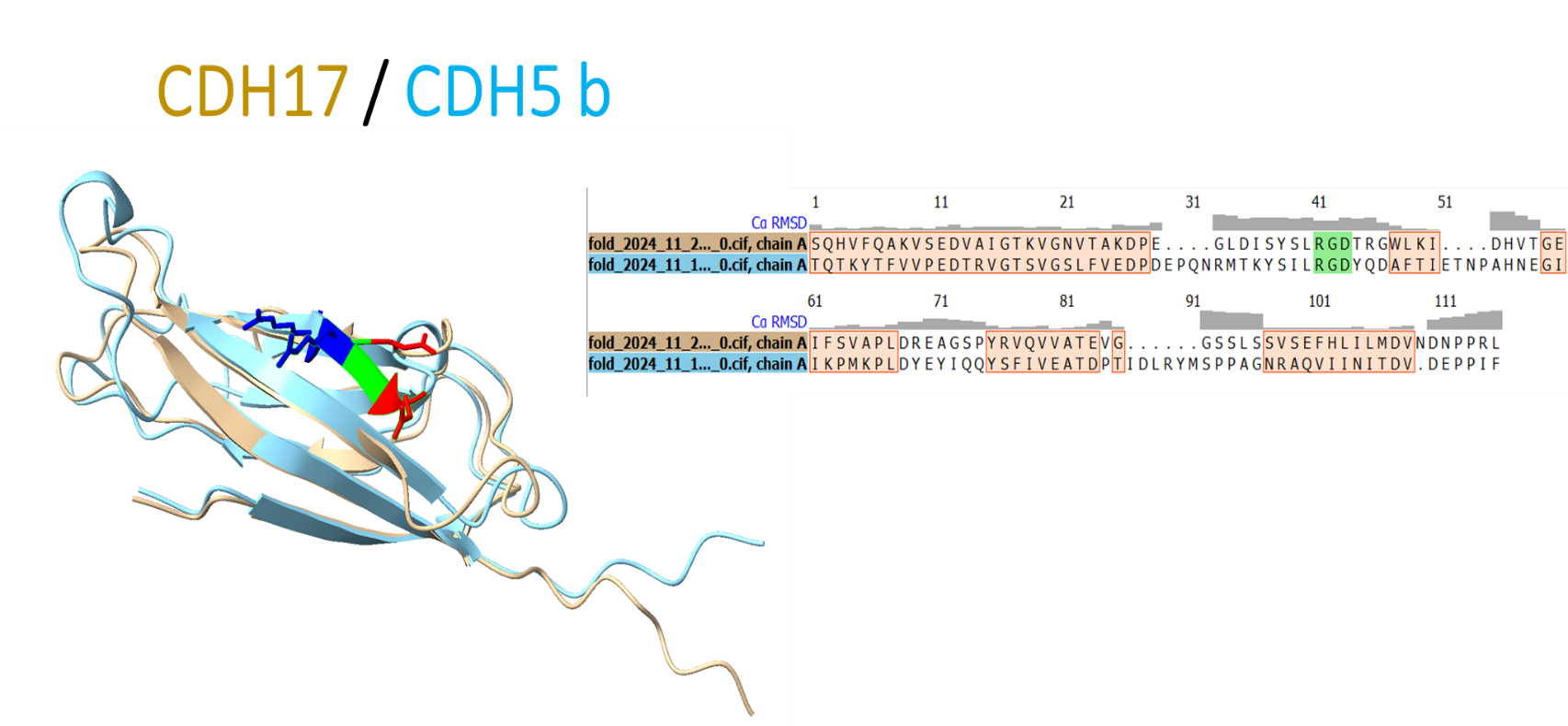


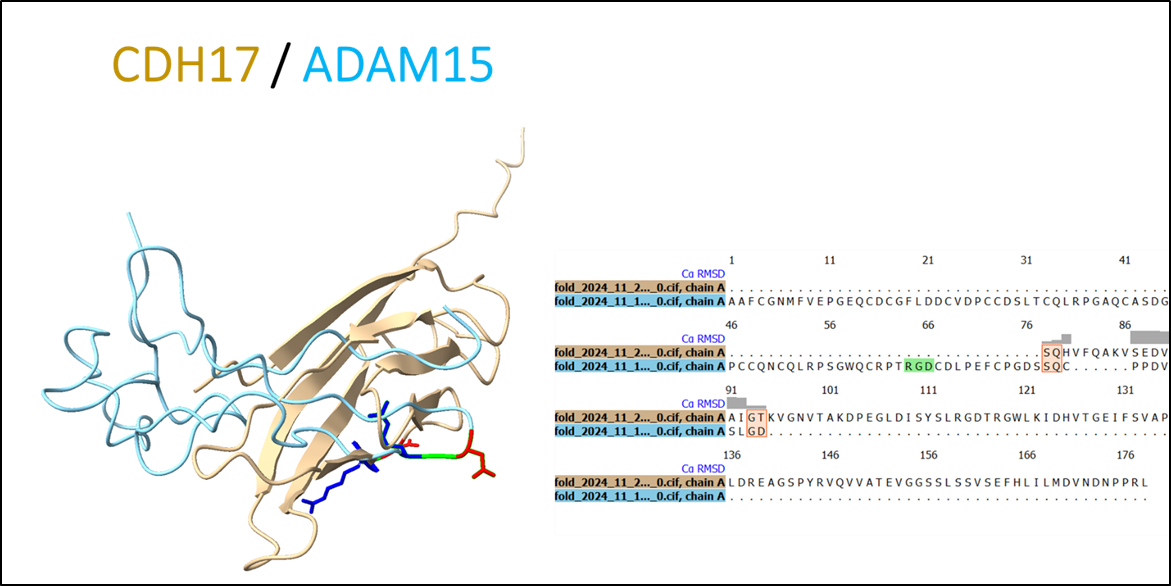


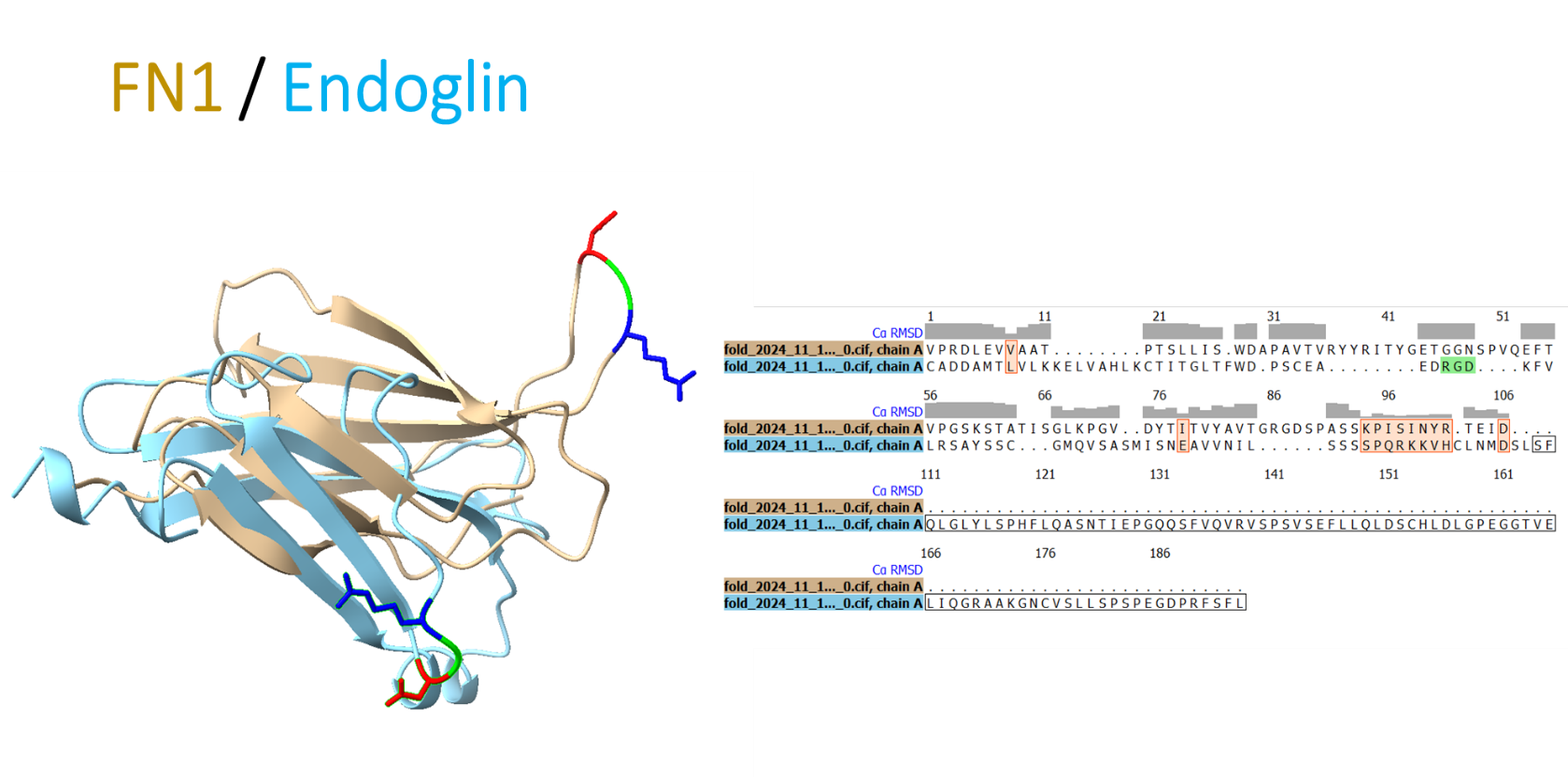
**E)**


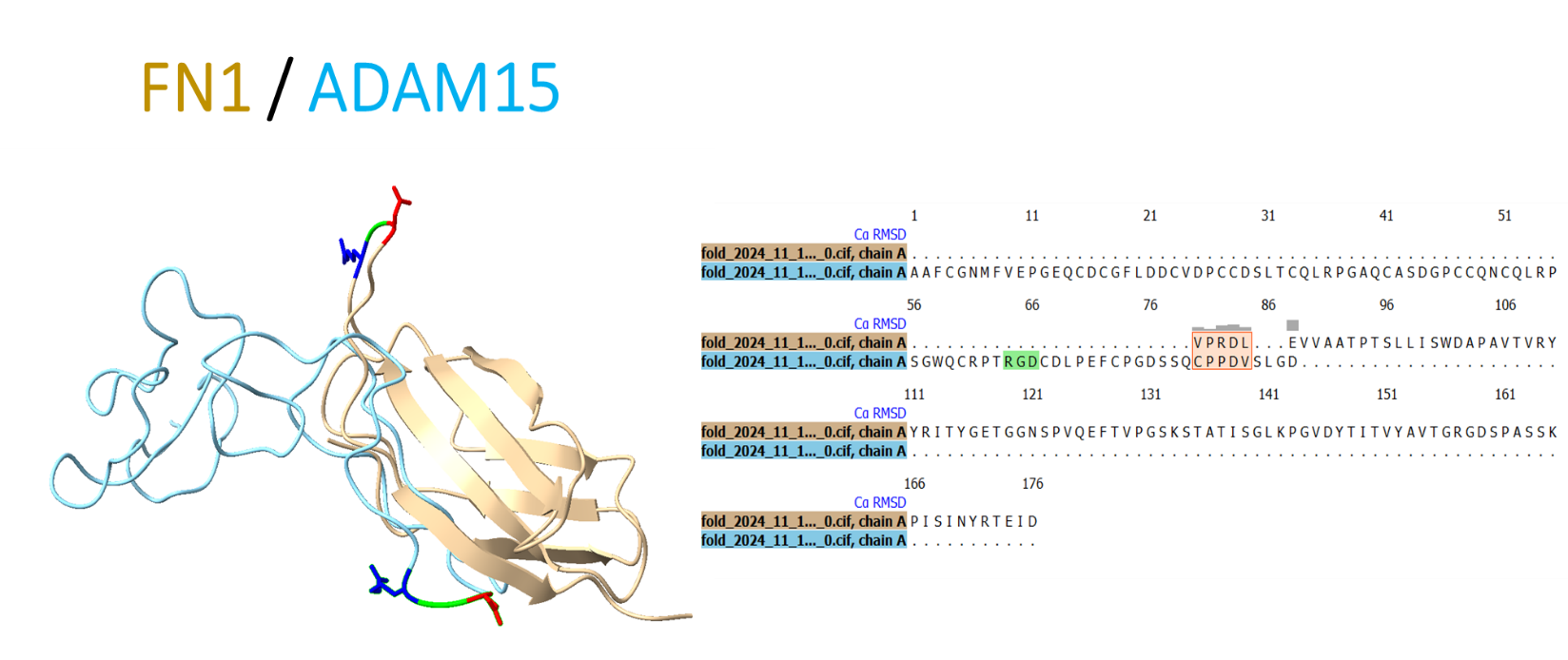

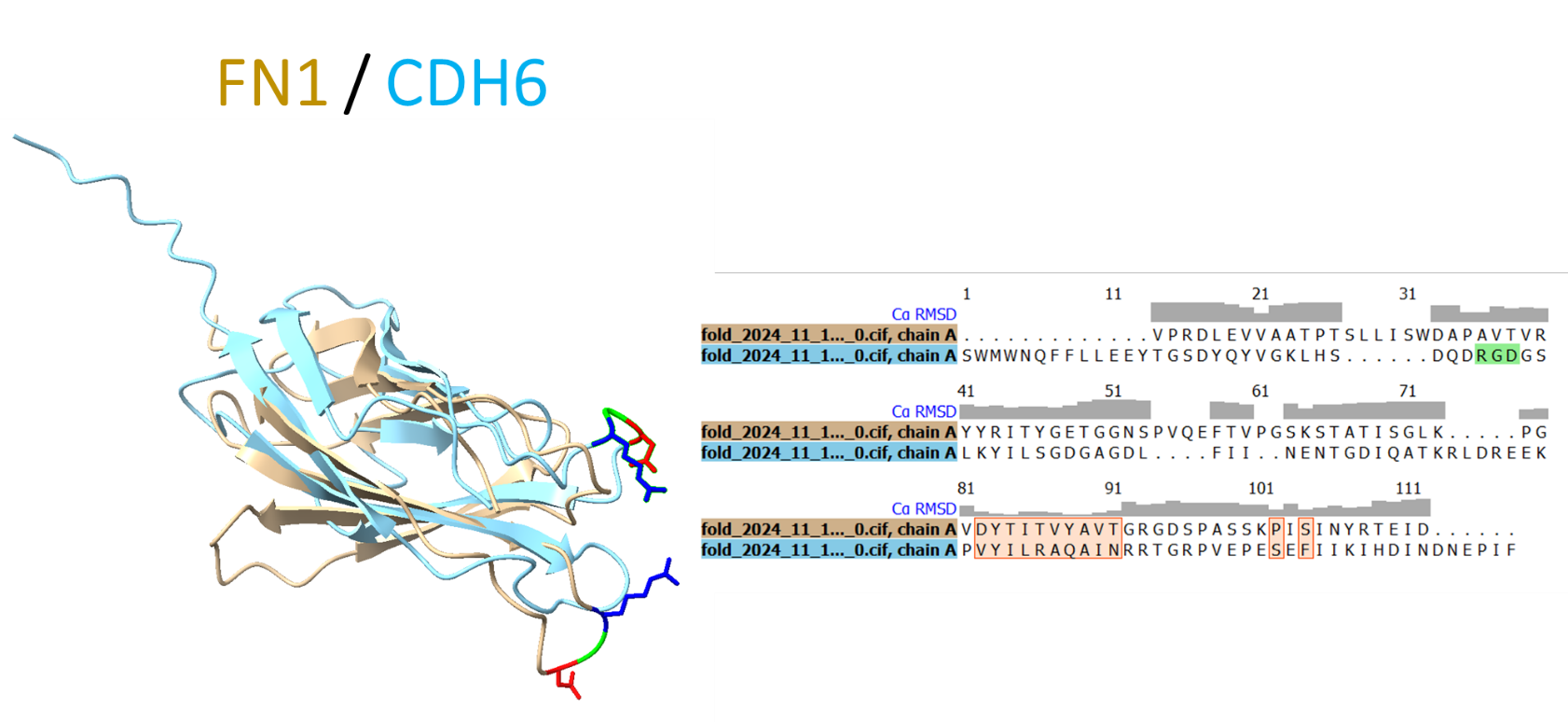

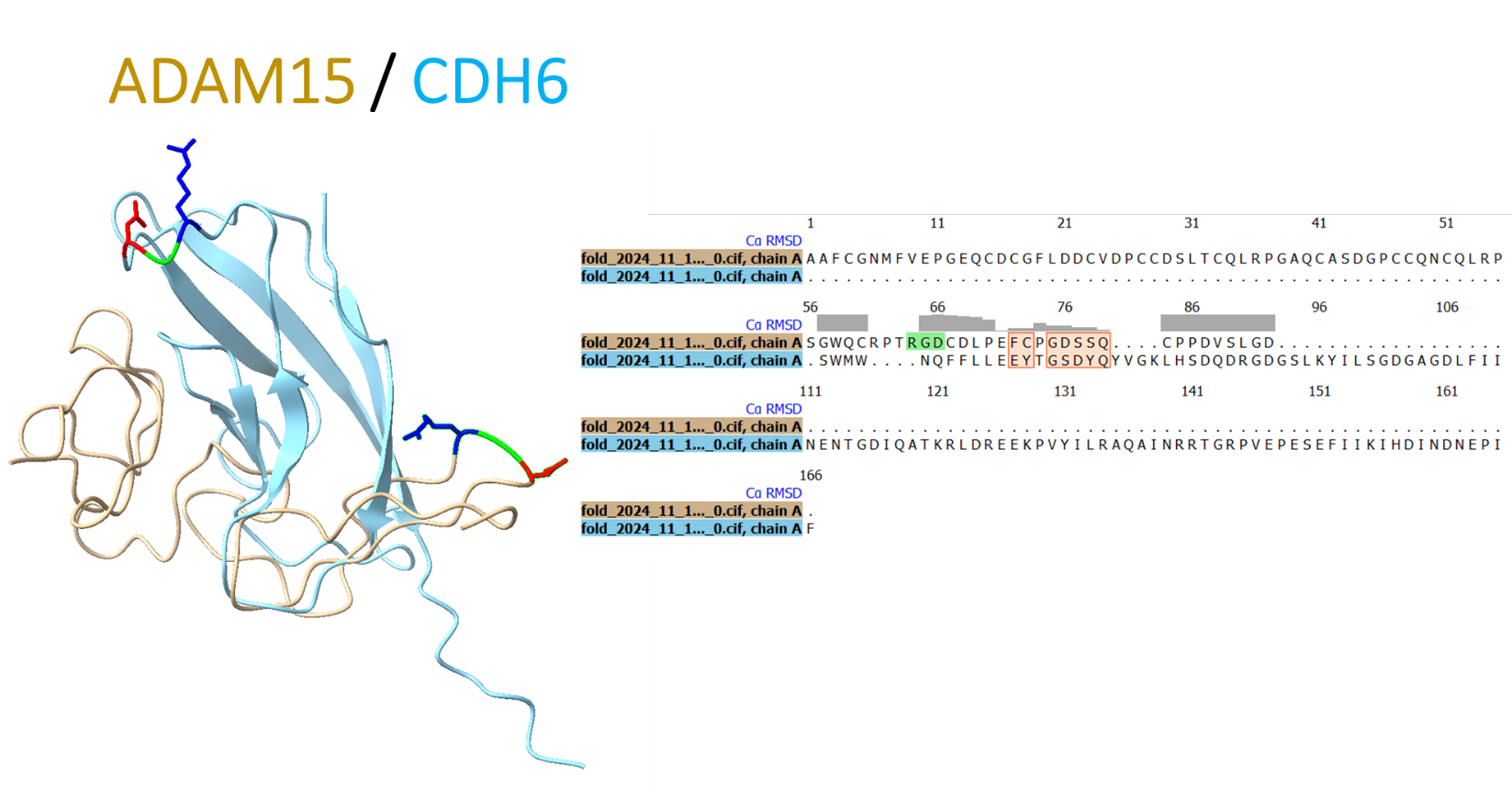
**F)**


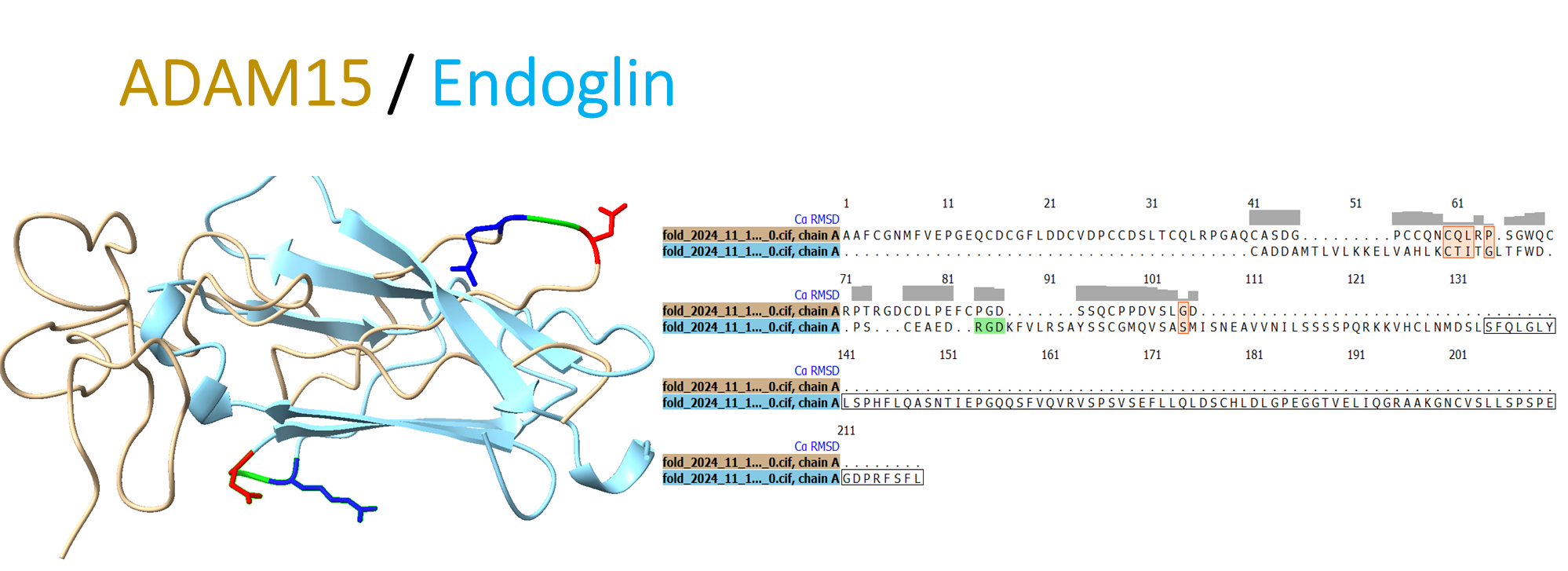


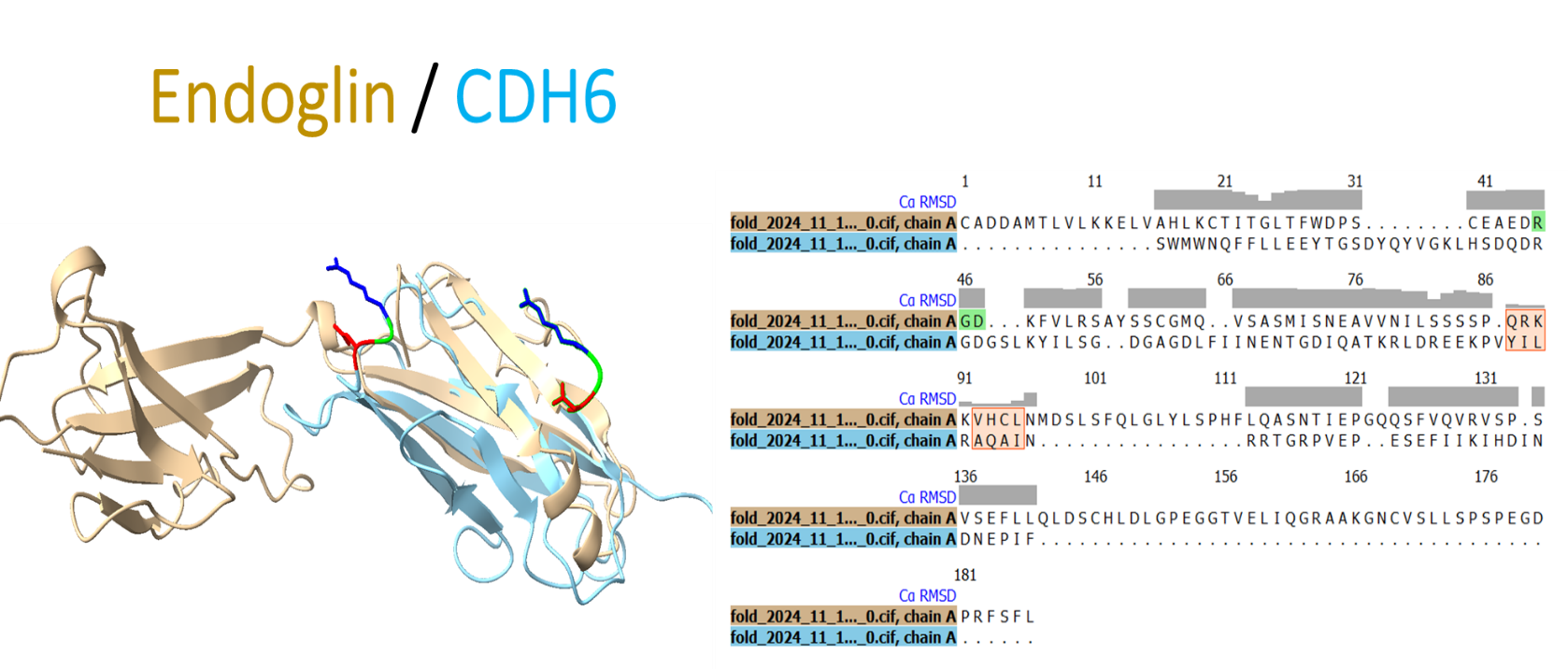
**G)**

**Supplementary Figure 3.** **Pair-wise comparisons of the 3D structures and RGD-containing sequences of integrin counter-receptors and FN1.** Each panel of this figure (**A, B, C, D, E, F, G**) includes, on the left, the pair-wise superimposed 3D structures of the RGD-containing integrin counter-receptors discussed in this review (as well as FN1) and, on the right, an alignment of the sequences surrounding the RGD motifs (stretching approximately 100 residues) of the respective domains (see Supplementary Figure 1) in the RGD counter-receptors and in FN1.

The position of the RGD motifs in 3D structures is highlighted by the following color code: **Arg-Gly-Asp**.
